# Obtention and Characterisation of Antioxidant-Rich Peptides from Defatted Grape Seed Meal Using Different Enzymes

**DOI:** 10.3390/foods14071248

**Published:** 2025-04-03

**Authors:** María del Rosario Rodríguez-Muñoz, Ana Belén Mora-Garrido, Francisco J. Heredia, María Jesús Cejudo-Bastante, María Lourdes González-Miret

**Affiliations:** Food Colour & Quality Laboratory, Facultad de Farmacia, Universidad de Sevilla, 41012 Sevilla, Spain; mrodriguez48@us.es (M.d.R.R.-M.); amgarrido@us.es (A.B.M.-G.); heredia@us.es (F.J.H.); miret@us.es (M.L.G.-M.)

**Keywords:** grape seed peptide hydrolysates, enzymes, warm climate, molecular weight distribution, antioxidant peptides

## Abstract

Defatted grape seed meal (DGSM) is a residue obtained from grape pomace and is an important source of protein. The aim of this study was to select peptides with optimal antioxidant and colour properties, obtained using enzymes of different origins and proteolytic character, for application in winemaking. For this purpose, the assay was performed using novo-ProD (NP), alcalase (AL), novozym (NZ), pepsin (PE), flavourzyme (FZ), and papain (PA) enzymes. The peptide percentage, peptide yield, molecular size of the peptide fractions, total amino acid, peptide content, antioxidant activity, and CIELAB colour coordinates of the hydrolysates were determined. The peptide hydrolysates obtained using PE showed the significantly (*p* < 0.05) highest percentages of peptides (93%), amino acid content (188 mg aa/g hydrolysate), and lightness (L*, 70.3). On the other hand, NP peptide hydrolysates displayed the significantly (*p* < 0.05) highest antioxidant activity (154 µmol TE/g hydrolysate) and peptide yield (39%). Regarding molecular weight (MW), PE led to hydrolysates with a lower proportion of low-MW peptides (MW < 1 kDa). In conclusion, the peptide hydrolysates obtained by NP and PE exhibited the greatest chemical characteristics for further application, both separately and combined in targeted hydrolysis, as colour stabilisers and antioxidant capacity enhancers in warm climate winemaking.

## 1. Introduction

The food industry is growing fast due to globalisation and population growth. This generates a large amount of waste, 158–298 kg/year/capita in the EU alone [1], with an important environmental impact since the removal process produces emissions and/or the accumulation of microorganisms, parasites, and pests [2]. Furthermore, there is a negative economic impact because the residue wasted worldwide represents an economic loss of USD 990 million [2]. For this reason, the food industry is focusing on obtaining new value-added products from waste, which, in turn, generates the development of a circular economy.

Within the food industry, the wine sector is one of the most important in terms of economic, cultural, and environmental frameworks in Spain and Europe. It is estimated that 25% of the total weight of grapes is disposed of as waste during the wine production process [3], which is classified into pomace, lees, and wastewater (around 62%, 14% and 12%, respectively) [4]. Due to its varied composition of bioactive compounds and the amount generated (around 7 t/year worldwide), grape pomace is of great interest. It constitutes around 10–20% of the weight of the grape mass and consists of seeds, stems, and skins [5]. In addition, grape pomace can be used in many different fields, such as food, pharmaceuticals, and cosmetics, because it has antioxidant and anti-inflammatory effects due to its high polyphenol content [6]. Moreover, after successive extractions to obtain different compounds that the grape pomace industry further sells, the seeds are separated from the pomace [7], dried, and the oil is extracted, thereby generating defatted grape seed meal (DGSM) as a residue, which is a rich protein source that can be further exploited [8].

Harvests of plants in a warm climate, i.e., grapevine, can be affected by climatic variables such as temperature and sunlight exposure [9]. Due to global warming, temperatures are increasingly warmer, which may cause the inadequate phenolic maturation of grapes, leading to a mismatch between the phenolic and technological (sugars) maturity of the grapes at the time of harvesting [10]. Among other effects, temperatures over 30 °C cause a decrease in the phenolic content [10], especially anthocyanins and copigments that participate in colour stabilisation phenomena such as copigmentation, produced by the union of anthocyanins with other wine components such as flavonols [11]. Due to the imbalance in phenolic content between pigments and copigments in wines from warm climates, it is difficult to maintain a stable colour during the storage period [12]. With the purpose of solving this problem, different strategies have been implemented to maintain the phenolic compound profile of these wines over time. One method involves the addition of peptide hydrolysates to wine during the stabilisation phase, which produces a peptide–phenolic complex responsible for colour preservation [13].

The peptide fraction must be soluble in the medium to produce the colour-stabilising complex. Enzymatic hydrolysis is an option for generating peptides with good solubility; it also has advantages over chemical hydrolysis, including better selectivity, moderate reaction conditions, reduced food allergenicity, and the capacity to not degrade compounds that have already been hydrolysed [14,15]. Cejudo-Bastante et al. [8] optimised a technique for obtaining peptide hydrolysates from defatted grape seed meal as a source of peptide using the enzyme alcalase, with positive results in terms of wine colour stabilisation [13]. Several factors can influence the properties of peptide hydrolysates, such as the substrate type, but one of the key factors is the type of protease used [14,16]. Thus, the use of certain proteases, like flavourzyme, can result in a higher proportion of medium- and low-MW peptides [17], which are more soluble [14]. Additionally, the amino acid composition of the peptides may be enriched in glutamine or histidine, potentially contributing to improved colour stabilisation [18] and increased antioxidant activity [19]. Therefore, it is essential to conduct studies using different enzymes to produce a peptide hydrolysate for use in winemaking that is not only soluble in wine but also exhibits high antioxidant and colour-stabilising properties.

This work mainly focuses on determining how the proteolytic character and the origin of the enzyme used affect the properties of the peptide hydrolysate, with the purpose of selecting optimal peptides for use as colour stabilisers in warm climate winemaking. Thus, an in-depth chemical characterisation based on peptide content, peptide yield, molecular weight (MW) distribution, amino acid content, peptide profile, antioxidant activity and the colour (CIELAB parameters) of the peptides was considered. This is the first time such a study has been conducted, and it could represent an important step towards innovative future applications in red wine colour stabilisation.

## 2. Materials and Methods

### 2.1. Chemical and Reagents

The endoproteases alcalase, novozym, and novo-ProD, and the endo- and exoprotease flavourzymes were provided by Novozymes (Copenhagen, Denmark). The endoprotease pepsin and the endo- and exoprotease papain were supplied by Sigma-Aldrich (St. Louis, MO, USA) and Biocon (Barcelona, Spain), respectively. Trichloroacetic acid (TCA) was supplied by VWR Chemicals (Radnor, PA, USA), and azocasein was purchased from Sigma Aldrich (St. Louis, MO, USA). The standards were cytochrome C (Panreac, Barcelona, Spain), aprotinin (Sigma-Aldrich, Madrid, Spain), vitamin B12 and triglycine (Alfa Aesar, Haverhill, MA, USA), and glycine (Thermo Fisher Scientific, Waltham, MA, USA).

### 2.2. Samples

The raw material, defatted grape seed meal (DGSM), a residue derived from the processing of the grape pomace industry, was supplied by Alvinesa Natural Ingredients S.A (Daimiel, Ciudad Real, Spain). Briefly, the grape pomace was washed in diffusion bands to extract components of interest (alcohol, tartaric salts, phenols, etc.) before being destemmed and dried. The grape seeds were then separated from the dried grape pomace by densiometric tables and were subsequently ground and granulated to proceed with the seed oil extraction using hexane, generating DGSM as a residue [8]. In the laboratory, the DGSM was ground to increase the surface area to achieve better protein extraction and was finally stored in darkness at room temperature.

### 2.3. Measurement of Protease Activity

The protease activity was measured according to Beynon and Bond [20]. Briefly, a reaction mixture of 0.1 g of azocasein, 0.2 mL of ethanol, and 4.8 mL of phosphate buffer (0.1 M at pH 7) was prepared and heated in a water bath (40 °C) until the components were completely dissolved. To achieve absorbance values within the range of 0.3–0.5, the enzyme solutions were prepared using distilled water: 1:4 dilution for NP, AL, and NZ; 1:8 dilution for FZ; 0.5% (*w*/*v*) for PE; and 0.05% (*w*/*v*) for PA. Subsequently, 100 μL of each enzyme solution was mixed with 100 μL of the reaction mixture and then incubated for 30 min at 40 °C in a water bath. After stopping the reaction with 5% (*w*/*v*) TCA and centrifuging the enzyme solutions (10,000× *g*, 4 °C, 4 min), the supernatants were collected. The azo dye released due to the hydrolysis of azocasein with proteases was measured at 440 nm in an Agilent 8453 UV-vis spectrophotometer (Agilent Technologies, Palo Alto, CA, USA) to calculate the protease activity for each enzyme. The samples were measured in triplicate, and the results were expressed as U/mL.

### 2.4. Peptide Hydrolysates from DGSM

Protein extraction was conducted according to the methodology described by Cejudo-Bastante et al. [8]. A mixture of 700 g of DGSM and 3.5 L of distilled water was prepared in a Bio Console ADI 1025 Bioreactor (Applikon Biotechnology, Delft, Netherlands). The mixture underwent constant agitation (180 rpm) at pH 10 using ammonia (NH_3_) as a basic medium for 3 h at 25 °C. The resultant protein concentrate obtained was centrifuged (14,880× *g*, 4 °C, 20 min) to separate the proteins from the non-protein precipitate. The supernatant was then collected and concentrated approximately to 1.5 L in a rotary evaporator (1 h, 80 °C) to prevent the formation of salts due to the presence of NH_3_, and this was adjusted to pH 8. Finally, the endogenous enzymes in the protein concentrate were deactivated in a water bath at 80 °C for 5 min.

Thereafter, the enzymatic hydrolysis of the protein concentrate was carried out in a bioreactor under optimised hydrolysis conditions for each enzyme, as detailed in Table 1. According to Cejudo-Bastante et al. [8], 0.6% (*v*/*v*) of each protease was added and reacted at the optimal hydrolysis time. The obtained crude hydrolysate was individually placed in a rotary evaporator (80 °C) until the volume was approximately reduced by half to reduce the pH for the subsequent step. Then, the pH of the crude hydrolysates was adjusted to 3.5 (the usual pH of wine) using 32% HCl. In the case of pepsin, the pH was 2.5; thus, it had to be increased up to 3.5 using NH_3_ to provoke the precipitation of the non-hydrolysed peptides. Finally, the supernatant (soluble peptides) was separated from the precipitate (insoluble peptides), which was discarded by centrifugation (15,000× *g*, 4 °C, 20 min), and the soluble fraction (peptide hydrolysate) was lyophilised. This procedure was performed in triplicate for each enzyme.

### 2.5. Peptide Content and Peptide Yield of Hydrolysates

The total nitrogen content of the peptide hydrolysates was determined using the standard Kjeldahl method [27] in an Automatic Kjeldahl Distiller system (J.P. Selecta, Barcelona, Spain). The percentage of the peptide content in the hydrolysates was calculated based on the nitrogen concentration measured using the following formula:

% N = [0.014 × (V) × (N)/(W)] × 100(1)

where % N is the nitrogen percentage; V is the volume (mL) of HCl; N is the normality of HCl; and W is the weight (g) of the sample. Factor 5.75 was considered for the conversion of total nitrogen content to peptide content (%) [8].

The peptide yield of the hydrolysates (peptide content (%) × weight (g) of the hydrolysate)/(weight (g) of the starting meal) was also determined.

### 2.6. Molecular Weight by Size-Exclusion Chromatography (SEC)

The molecular weight was determined according to the method described by Bautista et al. [28]. In total, 0.22 g of the peptide hydrolysate was dissolved in 10 mL milli-Q water. After shaking (2500 rpm, 10 min) and centrifugation (3220× *g*, 4 °C, 5 min), the supernatant was filtered through a 0.45 µm filter. An Agilent 1100 chromatography system equipped with a quaternary pump, an automatic injector, a UV-vis diode array detector and software B.04.03 version (Agilent Technologies, Palo Alto, CA, USA) was used for the analysis, using a SuperdexTM 30 Increase 10/300 GL column (optimum separation range 0.1–7 kDa). For the separation of peptide fractions, a flow rate of 0.5 mL/min of 50 mM Na_2_HPO_4_ solution (pH 7.5) was used in isocratic mode at 25 °C. According to the manufacturer’s indications, different standards were used to cover the range 100–7000 Da: cytochrome C, 12,500 Da; aprotinin, 6500 Da; vitamin B12, 1355 Da; triglycine, 189 Da; and glycine, 75 Da. The peptide fractions were monitored at 215 and 280 nm. The analysis was performed in triplicate for each peptide hydrolysate.

### 2.7. Amino Acid Analysis

To determine the individual and total content and profile of amino acids in the peptide hydrolysates, ion exchange chromatography and post-column derivatisation using ninhydrin were used [8]. A total of 5 mg of each sample was mixed with 1 mL of 6 M HCl and hydrolysed in a heating block at 110 °C for 24 h. After centrifugation (11,357× *g*, 4 °C, 15 min), supernatants were collected, and pH was adjusted to 2.0 using 6 M and 1 M NaOH. In total, 400 μL of each sample was mixed with 100 μL of the internal standard norleucine at 50 μM. Finally, the samples were analysed using a Bio 30+ Amino Acid Analyser system (Biochrom Ltd., Cambridge, UK), equipped with a high-pressure PEEK cation exchange column with Ultropac 8 cation exchange resin and a UV-vis detector. The detection wavelengths were 440 nm for proline and 570 nm for the other amino acids. The amino acid content of each sample was quantified in triplicate, and the results were expressed in mg/g of peptide hydrolysate.

### 2.8. Peptide Identification

Samples were desalted and concentrated using OMIX Pipette tips C18 (Agilent Technologies, Palo Alto, CA, USA). The desalted peptide hydrolysates were dried, resuspended in 0.1% formic acid (10 mL) and analysed using RP-LC-MS/MS in an EASY-nLC II system coupled to an ion trap LTQ-Orbitrap-Velos-Pro hybrid mass spectrometer (Thermo Scientific, Waltham, MA, USA). The peptides were concentrated by reverse-phase chromatography (0.1 mm × 20 mm C18 RP Precolumn, Thermo Scientific) and separated using a 0.075 mm × 250 mm C18 RP column with a flow of 0.3 μL/min. Then, a 180 min dual gradient with the following profile was used to elute the peptides: 5–25% solvent B for 135 min, 25–40% solvent B for 45 min, 40–100% solvent B for 2 min, and 100% solvent B for 18 min (solvent A: 0.1% formic acid in water, solvent B: 0.1% formic acid, 80% acetonitrile in water). Electrospray ionisation (ESI) was performed using a Nano-bore emitter made of stainless steel with an inner diameter of 30 μm (Proxeon, Odense, Denmark) interfaced at a spray voltage of 2.1 kV with an S-Lens efficiency of 60%. The Orbitrap resolution was set to 30,000. Survey scans were conducted within the range of 400–1600 atomic mass units (amu) (1 µscan), followed by 20 sequential data-dependent MS/MS scans (Top 20). The isolation width for MS/MS was set at 2 u (in mass-to-charge ratio units), with a normalised collision energy of 35%, and dynamic exclusion was implemented for 60 s intervals. Charge-state screening was activated to discard unassigned and singly charged protonated ions. The results were then matched with the UniProt database and Peaks X Pro software (version X Pro) [8].

### 2.9. Antioxidant Activity

#### 2.9.1. DPPH Assay

The method described by Soler-Rivas et al. [29] was applied to evaluate the antioxidant activity of the peptides. In total, 0.02 g of each hydrolysate was dissolved in 10 mL of PBS. Then, to formulate a calibration curve, 300 µL of 108 µM DPPH methanolic solution was added to 30 µL of the sample, standard Trolox or 80 % (*v*/*v*) blank of methanol, and finally, the mixture was diluted with 570 µL of 80% (*v*/*v*) methanol. After 30 min in the dark, absorbance was measured at 515 nm in an Agilent 8453 UV-vis spectrophotometer (Agilent Technologies, Palo Alto, CA, USA). The analysis was performed in triplicate, and the results were expressed as Trolox equivalents (TE)/g peptide hydrolysate.

#### 2.9.2. ABTS Free Radical Scavenging Assay

The antioxidant activity was determined using the ABTS method, as described by Re et al. [30]. First, 7 mM ABTS•+ stock solution was prepared using persulfate 2.45 mM as the oxidising agent and stored in the dark at room temperature for 12–16 h. Subsequently, the working reagent of ABTS and PBS (1 mL of ABTS and 79 mL of PBS) was prepared, adjusting its absorbance to 0.7 ± 0.02 by measuring the absorbance at 734 nm with an Agilent 8453 spectrophotometer (Agilent Technologies, Palo Alto, CA, USA). Subsequently, a pre-dilution test was performed on the samples, using dilutions of 1/10, 1/20 and 1/40 for each sample in triplicate. Finally, 2 mL of the working reagent was added to 50 μL of each dilution, and the absorbance at 734 nm of each sample was measured. The results were expressed as Trolox equivalents (TE)/g samples.

### 2.10. Colorimetric Analysis

A CM-5^®^ spectrophotometer (Konica Minolta, Tokyo, Japan) was used to measure the colour of the peptide hydrolysates by diffuse reflectance, similar to other authors [31]. The CIELAB colorimetric parameters (L*, a*, b*, C*_ab_, and h_ab_) were obtained following the recommendations of the Commission Internationale de L’Eclairage [32] using the CIE 1964 10° Standard Observer and the Standard Illuminant D65 for the calculation. Measurements were performed in triplicate for each peptide hydrolysate sample (obtained with each enzyme). From the colour data, considering the Euclidean distance between two points in the three-dimensional CIELAB colour space, the colour differences (ΔE*_ab_) between pairs of samples were calculated using the following CIE 1976 colour difference formula: ΔE*_ab_ = [(ΔL*)^2^ + (Δa*)^2^ + (Δb*)^2^]^1/2^. Furthermore, the relative contributions of lightness (% ΔL), chroma (% ΔC), and hue (% ΔH), which make a given colour difference (ΔE*_ab_) expressed as percentages, were calculated as follows:

% Δ*L* = [(ΔL*)^2^/(ΔE*_ab_)^2^] × 100

% Δ*C* = [(ΔC*_ab_)^2^/(ΔE*_ab_)^2^] × 100

% Δ*H* = [(ΔH)^2^ /(ΔE*_ab_)^2^] × 100

Δ*H* is deduced mathematically from Δ*H* = [(ΔE*_ab_)^2^ − (ΔL)^2^ − (ΔC)^2^]^1/2^.

### 2.11. Statistical Analysis

Statistica v.8.0 software was used to perform the statistical analysis of the data [33]. A univariate analysis of variance (ANOVA) using a general linear model programme was used to establish whether the means of the sample data significantly differed from each other. Principal component analysis (PCA) was also conducted to identify the main contributors to variance.

## 3. Results and Discussion

### 3.1. Protease Activity

The amount of enzyme required for the hydrolysis process was calculated and adjusted as a function of its corresponding proteolytic activity to ensure that the parameters measured in the hydrolysates did not depend on this factor. The proteolytic activities of all the enzymes were determined (Table 2). The enzyme FZ was found to provide significantly (*p* < 0.05) higher protease activity, whereas those with significantly (*p* < 0.05) lower activity were PE and PA. Finally, considering the enzyme AL as a reference, enzyme volumes leading to the same proteolytic activity were adjusted and added.

### 3.2. Peptide Percentage and Yield of the Peptide Hydrolysates

The peptide content of the hydrolysates ranged from 61% to 94% (Table 3). These results are comparable to those of other peptide hydrolysates derived from different oil seeds [34]. However, it was 2.39% when related to DGSMs. As shown in Table 4, the origin of the enzymes was an influential factor in the peptide content. In this regard, the significantly (*p* < 0.05) higher % peptide was attributed to the hydrolysates obtained by animal enzymes (PE). In contrast, the hydrolysates obtained by the microbial AL displayed the significantly (*p* < 0.05) lowest values. The specificity of alcalase for internal bond cleavages [35] could be related to the lower peptide percentage of the hydrolysates obtained by AL, as the polypeptides obtained could remain insoluble in the extraction medium due to their size.

Concerning the peptide yield, both the origin of the enzymes proteolytic character were significantly (*p* < 0.05) influenced (Table 4). By far, vegetal enzymes (PA) and the endo-exo proteases (PA and FZ) generated significantly (*p* < 0.05) lower amounts of peptide hydrolysates (Table 3) and were the less efficient in terms of the peptide extracted. According to Ahmahd Nadzri et al. [36], papain cleaves peptide bonds predominantly at the C-terminal, causing the formation of a considerable quantity of amino acids compared to that of peptides. As a result, those formed peptides could have high molecular weights, making their solubility diminish and, consequently, lead to a low yield of peptide hydrolysate. On the other hand, the peptide hydrolysates by NP and PE showed the significantly (*p* < 0.05) highest peptide yield (Table 3).

Based on the results, and bearing in mind their subsequent use in winemaking, NP and PE could be the optimum enzymes for conferring the highest peptide content and peptide yield of the derived peptide hydrolysates.

### 3.3. Molecular Weight Distribution

Table 3 shows the percentages of peptide fractions for each peptide hydrolysate divided into three MW ranges: larger than 5 kDa, between 5 and 1 kDa, and less than 1 kDa. In general, the greatest proportion corresponded to the MW < 1 kDa fraction for all samples, showing that all the hydrolyses were properly developed. Similar results have been reported, such as by Meinlschmidt et al. [37], who investigated how to decrease the allergenicity of soy proteins by enzymatic hydrolysis using different enzymes (alcalase, flavourzyme, papain, colorase 2TS, and pepsin).

Both the origin of the enzymes and their proteolytic character influenced the percentage of the low-MW fraction achieved after enzymatic hydrolysis, according to ANOVA analysis (Table 4). The FZ-derived peptides displayed a significantly (*p* < 0.05) higher percentage of this fraction, whereas the lowest percentage was ascribed to those obtained by PE. This could be supported by the fact that FZ and PA exhibited exopeptidase activity, which favours the formation of single amino acids rather than longer polypeptides [35]. Regarding the 1–5 kDa fraction, the proteolytic character was the most important factor regarding the sample’s differentiation, with endoprotease-derived peptides displaying a higher percentage of this fraction (NP, AL, NZ, and PE). However, the origin exerted a more remarkable effect (*p* < 0.05) on the MW > 5 kDa peptide fraction, with the lowest percentages expressed in microbial-derived (NP, AL, FZ, and NZ) peptides.

Based on the results, FZ appeared to be the most suitable enzyme for potentially using peptide hydrolysates in winemaking due to its effectiveness in producing low-MW peptide fractions. However, the other microbial enzymes (NP, AL, and NZ) could also be considered, though they would yield peptides with medium MW peptides.

### 3.4. Amino Acid Content

Table 5 summarises the composition of soluble amino acid at pH 2 of the peptide hydrolysates obtained with the different enzymes, showing the individual and total amino acids (TAAs) and their classification into aromatic (AAA: His, Phe, and Tyr), sulphur (SAA: Met and Cys), and hydrophobic (HAA: Ala, Val, Leu, Ile, Met, and Phe) amino acids.

In accordance with Cejudo-Bastante et al. [8], it was observed that, regardless of the enzyme used, the most abundant individual amino acid in all peptides was Glu, followed by Gly, which constituted 31% and 7%, respectively. On the other hand, the minor amino acids present in all the peptides were Cys (constituted approximately 0.4%) and Met (around 0.8%), in agreement with the results achieved by Mora-Garrido et al. [13] in grape seed peptide hydrolysates with alcalase.

Notably, the hydrophobic amino acid family constituted the largest proportion of the total amino acid content (approximately 22%), followed by the aromatic amino acids, which accounted for approximately 7% (Table 5). The sulphur-containing amino acids were the least abundant, comprising only 1%, which is in agreement with Arrutia et al. [38], who also found that the SAA concentration of other seeds such as sunflower, soybean or rapeseed is exceptionally low with respect to other types of amino acids. Cejudo-Bastante et al. [8] obtained similar percentages of those groups of amino acids (HAA, AAA, and SAA) for grape seed meal hydrolysates with alcalase.

According to Table 4, both the origin and proteolytic character of the enzymes were factors influencing the individual and total amino acid (TAA) content, as well as the distribution among the different amino acid families (SAA, HAA, and AAA). Remarkable variations in the TAA content were observed among the peptides, with those obtained by PE (animal enzyme) having a significantly (*p* < 0.05) higher concentration, as opposed to those obtained by PA (vegetal enzyme) (Table 3), especially Asp, Val, Ile, Phe, His, Lys, and Arg. In addition, the use of endoproteases (NP, AL, NZ, and PE) led to peptide hydrolysates with a higher content of TAA. As expected, this behaviour was in concordance with the peptide yield values, as similar trends were observed.

Regarding the AAA content, significant (*p* < 0.05) differences were observed among the peptides, following a similar trend to the TAA content, i.e., PE peptides exhibited the highest amounts, especially Phe. The differences in the HAA content were also evident among the peptides. Those obtained by PE (animal origin) showed distinct HAA levels compared with those produced using microbial enzymes (NP, AL, NZ, and FZ) and the plant-derived enzyme PA, mainly Val, Ile, and Phe (Table 5). This behaviour can be attributed to two factors: (i) the raw material (DGSM) contains a high proportion of hydrophobic and aromatic amino acids [39], and (ii) pepsin preferably cleavages the bonds adjacent to both [40]. In terms of the SAA content, peptides obtained by the endoproteases (NP, AL, NZ, and PE) significantly (*p* < 0.05) differed from those of the endo-exoproteases, according to ANOVA analysis (Table 4), which suggests a relationship between proteolytic activity and SAA, especially for Cys.

According to Chamizo-González et al. [41,42,43], the amino acids Arg and Phe could establish interactions with malvidin-3-glucoside, the major anthocyanin responsible for the colour of red wines, proposing pepsin as a potential enzyme to use peptide hydrolysates in winemaking based on its amino acid content.

### 3.5. Peptide Composition

The peptide sequences of the different peptide hydrolysates determined by RP-LC-MS/MS are shown in Table S1. All hydrolysates had between 7 and 20 amino acids in the peptide sequences, with MW ranging from 703 to 2815 Da in the same framework described by Mora-Garrido et al. [13] on defatted grape seed peptide hydrolysates with alcalase. Among all the proteins from which the identified peptides were derived, only five (F6HZK2, F6HZK3, A5CL75, and F6TY5) were present in all hydrolysates. Notably, the protein F6I0M9 was the only common protein in the hydrolysates obtained by FZ and PA, suggesting that their derived peptides may be a characteristic marker of peptide hydrolysates generated by endo-exopeptidases. Peptides derived from the proteins F6HZK2 and F6HZK3 have reported a high binding affinity with malvidin-3-glucoside [18,42], which could make the achievement of colour stabilisation in red wines feasible. Furthermore, according to López-Molina et al. [18], the peptides derived from F6HZK2, F6HZK3, and A5CL75 have minimal hydrophobicity, which facilitates their interaction with malvidin-3-glucoside in a watery medium such as wine.

Therefore, it is of great importance to characterise both peptides and terminal amino acids to elucidate how their addition to red wines could improve colour stabilisation and antioxidant activity. In fact, according to Zou et al. [44], amino acids found at the C-terminal were more important in predicting the antioxidant capacity of peptides than those found at the N-terminal.

The amino acid distribution at the terminal positions of each peptide, extracted from the results in Table S1, is shown in Table 6.

Concerning the N-terminal position of the peptides, Glu and Gln (negative residues) were the predominant amino acids, except in the peptides obtained by PE, in which Val was identified. In light of this fact, malvidin-3-glucoside could be interacting with the peptide at the N-terminal, as it preferentially binds with those containing Glu amino acids in their sequence [18]. This interaction is primarily stabilised by Van der Waals forces and hydrogen bonds, which contribute to copigmentation and provide protection to the malvidin molecule, enhancing its stability [45]. On the other hand, at the C-terminal position, mainly Gln, Arg, Leu, Asp, and Phe were found. The presence of hydrophobic amino acids (Val, Phe and Leu) at the N-terminal or C-terminal is associated with the high antioxidant activity of the peptide [46].

### 3.6. Antioxidant Activity

The antioxidant activity values obtained by DPPH and ABTS for the peptides are shown in Table 7 and are separately discussed. These methods differ in their transfer mechanisms [47]. The DPPH method relies on hydrogen atom transfer (HAT, Hydrogen Atom Transfer), whereas the ABTS method involves both HAT and single electron transfer (SET, single electron transfer).

According to Table 4, proteolytic character significantly (*p* < 0.05) influenced the antioxidant activity using DPPH. In this regard, peptides obtained by endo-exoproteases exhibited the lowest values, especially those in which PA was used (Table 7). Among the rest, peptides obtained by NP had, by far, the significant (*p* < 0.05) and highest antioxidant capacity, followed by those obtained by PE.

There are some discrepancies concerning the relationship between antioxidant activity and molecular weight; some authors reported a direct relation between low-molecular-weight peptides and antioxidant capacity [17,48], contrary to the findings of Zheng et al. [49]. It seems that other determining factors could exert a more remarkable influence on the antioxidant capacity, such as the amino acid composition or the amino acid position within the peptide sequence [50,51,52]. Based on different studies [53,54], the presence of hydrophobic amino acids in the hydrolysates may be related to the antioxidant activity determined by DPPH. On the other hand, amino acids such as Arg, Asp, and Gly at the C-terminal position of the peptide sequence have been revealed as enhancers of antioxidant activity [55]. These findings could explain our results in terms of the highest percentage proportion of Arg seen at the C-terminal position in peptides obtained by NP (Table 6) and some hydrophobic amino acids, such as His, which were seen in peptides obtained by PE (Table 6), directly related to the observed antioxidant activity. Based on the results, NP appears to be the most appropriate enzyme, in terms of antioxidant activity, for applying peptide hydrolysates in winemaking, contributing positively to avoid or retard the oxidation.

Regarding ABTS, no significant differences were observed in the antioxidant activity among the peptides, as shown in Table 4 and Table 6. This finding is consistent with the lack of significant differences in Cys, Trp, His, and Tyr (Table 5) in light of their relationship with the antioxidant activity of ABTS [19].

### 3.7. Colour Properties

Table 3 shows the CIELAB colour parameters (L*, C*_ab_, and h_ab_) of the peptide hydrolysates. All samples were located in the first quadrant of the (a*b*) plane. The dark colour of all hydrolysates can be ascribed to the use of an alkaline solution to extract protein (Xu & Diosady, 2002) [56]. This causes the oxidation of phenolic compounds to quinones, which eventually bind to proteins, resulting in a decrease in lightness [56,57]. Moreover, Ahmed et al. [58] reported that the pH change during hydrolysis using alcalase caused the pigments in the lentil hydrolysate to become unstable, resulting in a colour change.

As can be observed in Table 4, the colour parameters (L*, C*_ab_, and h_ab_) of the peptide hydrolysates were mainly influenced by the origin of the enzymes. Thus, the enzymes of microbial origin, especially FZ, displayed significant (*p* < 0.05) lower L* values, producing darker hydrolysates compared with those of animal (PE) or vegetal (PA) origins (Table 3). In addition, these results are consistent with those of the h_ab_ parameter, which showed that the enzymes of animal (PE) and vegetal (PA) origins produced browner peptide hydrolysates than those of microbial origin. In terms of chroma, the peptide hydrolysates obtained by vegetal origin enzymes (PA) had less colour intensity.

Considering the colour differences and the contribution of each parameter, it is noteworthy that the lightness (Δ^2^L*) was the attribute that mainly contributed to the colour differences with respect to the total colour (83–94%). These colour differences were visually perceptible (ΔE*_ab_ > 3 CIELAB units) when comparing the peptide hydrolysates obtained by microbial enzymes (NP, AL, NZ, and FZ) with those obtained by vegetal (PA) and animal (PE), as well as between the peptide hydrolysates obtained by vegetal and animal enzyme. Therefore, as the most appropriate product for use in winemaking is one that does not alter the colour of the wine, the hydrolysates obtained by PA appeared to be suitable, as they exhibited a high lightness value and a more greyish tonality.

### 3.8. PCA

**Figure 1:**
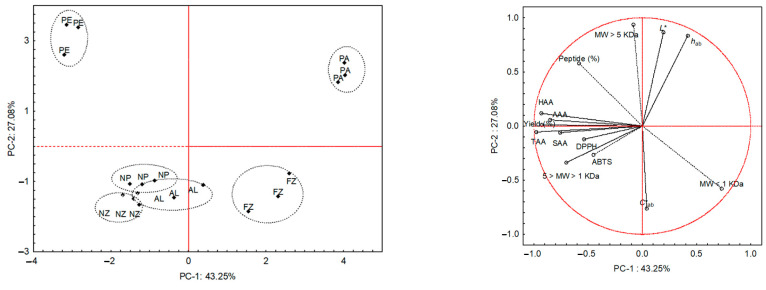
Scatterplot of samples (NP, novo-ProD; AL, alcalase; NZ, novozym; PE, pepsin; FZ, flavourzyme; PA, papain) and variables (peptide content and yield, molecular weight distribution, TAA, total amino acids; SAA, sulphur amino acids; HAA, hydrophobic amino acids; AAA, aromatic amino acids; antioxidant activity DPPH and ABTS and CIELAB parameters) plotted onto the plane defined by the first two principal components.

To determine the parameters responsible for the differences between the samples, an unsupervised pattern recognition statistical analysis (principal component analysis, PCA) was carried out (Figure 1). According to the Kaiser criterion (eigenvalues > 1), fourteen significant principal components were identified, explaining 100% of the total variance. Figure 1 shows the samples in the plane defined by the two principal components (PCs), which explain 70.33% of the total variability. The PC-1 divided the samples based on the proteolytic character of the enzymes used to obtain the peptide hydrolysates. Thus, all the hydrolysates obtained by endoproteolytic enzymes were located in the negative axis, distinguishing from those obtained with endo-exoproteolytic enzymes (FZ and PA) by their higher intermediate and high molecular weights (1 kDa < MW < 5 kDa and MW > 5 kDa) and colorimetric parameters (L*, C*_ab_ and h_ab_). Moreover, PC-2 separated the samples according to the origin of the enzymes used to obtain the peptide hydrolysates, locating those obtained by microbial enzymes (NP, AL, NZ, and FZ) on the negative axis, mainly due to antioxidant activity, chroma (C*_ab_), and low and intermediate molecular weight (MW < 1 kDa and 1 kDa < MW < 5 kDa).

## 4. Conclusions

This study aimed to determine the optimal oenological peptide hydrolysates for subsequent additions to red wines to improve qualities such as colour stabilisation and further oxidations. According to the results, the chemical characteristics of the peptide hydrolysates are influenced by enzymatic factors. Specifically, the influences of origin on the CIELAB colour parameters produce microbial enzymes with darker hydrolysates than the vegetal or animal enzymes. On the other hand, the proteolytic character mainly impact the antioxidant activity, providing the hydrolysates obtained by endopeptidases with the highest values. Based on all the studied parameters, the peptide hydrolysates obtained by NP displayed the highest antioxidant activity and peptide yield, while those obtained by PE exhibited the greatest values of peptide content and lightness. Therefore, NP and PE enzymes, obtained either individually or in combination by targeted hydrolysis, could have the potential to produce peptides with favourable chemical properties, which could enhance antioxidant capacity and stabilise colour in warm-climate winemaking. This study brings new insights for future protease applications for elaborating on high-quality red wines produced in warm climates. Nevertheless, as this is the first study on obtaining and characterising peptide hydrolysates from an oenological source (grape seed residue) using different enzymes in such a detailed way, further research is needed to prove their effectiveness in wines as colour stabilisers.

## Figures and Tables

**Table 1:** The origin, proteolytic character, and optimal hydrolysis conditions of the different enzymes.

Name	Origin of Enzyme	Proteolytic Character	pH	T (°C)	Time (h)	Bibliography
NP	Microbial	Endoproteolytic	9	60	2	[21,22]
AL	Microbial	Endoproteolytic	8.5	55	1	[13]
NZ	Microbial	Endoproteolytic	9	50	2	[23,24]
PE	Animal	Endoproteolytic	2	33	3.5	[25]
FZ	Microbial	Endo-/exoproteolytic	7	50	2	[21,26]
PA	Vegetal	Endo-/exoproteolytic	7	50	3.5	[22,25]

NP, novo-ProD; AL, alcalase; NZ, novozym; PE, pepsin; FZ, flavourzyme; PA, papain.

**Table 2:** Mean values and standard deviations of protease activity (U/mL) for the different enzymes.

	Protease Activity (U/mL)
NP	703.84 ± 64.81 b
AL	807.53 ± 5.03 b
NZ	786.8 ± 73.79 b
PE	311.7 ± 11.99 c
FZ	1104.19 ± 40.5 a
PA	332.56 ± 11.04 c

NP, novo-ProD; AL, alcalase; NZ, novozym; PE, pepsin; FZ, flavourzyme; PA, papain. Different letters in the same column indicate significant (*p* < 0.05) differences according to Tukey’s test.

**Table 3:** Mean and standard deviation of percentages of peptide content, peptide yield, molecular weight (MW) distribution, and the CIELAB colour parameters (L*, C*_ab_, h_ab_) of the peptide hydrolysates obtained by the different enzymes.

	Peptide Content (%)	Peptide Yield (%)	Molecular Size Fractions (%)			Colour Parameters (CIELAB Units)		
			>5 kDa	5 > MW > 1 kDa	<1 kDa	L*	C*_ab_	h_ab_
NP	76.61 ± 1.62 b	39.18 ± 0.83 a	20.07 ± 0.11 d	38.20 ± 0.71 a	41.73 ± 0.67 bc	54.51 ± 2.35 b	39.79 ± 2.78 b	64.82 ± 0.57 cd
AL	65.89 ± 4.24 d	30.52 ± 1.97 b	22.05 ± 0.25 cd	36.00 ± 0.50 a	41.95 ± 0.27 bc	53.98 ± 4.22 b	41.56 ± 1.11 b	64.32 ± 0.83 de
NZ	77.18 ± 0.59 b	31.63 ± 0.24 b	20.49 ± 1.73 d	38.48 ± 2.29 a	41.03 ± 0.66 c	52.60 ± 4.85 bc	40.23 ± 1.44 b	62.26 ± 0.18 e
PE	93.17 ± 0.34 a	38.29 ± 0.14 a	33.84 ± 0.57 a	33.23 ± 0.66 b	32.92 ± 1.23 d	70.29 ± 5.42 a	33.42 ± 0.27 bc	69.31 ± 1.07 ab
FZ	75.66 ± 0.76 bc	20.83 ± 0.21 c	23.10 ± 0.83 c	25.98 ± 0.91 c	50.93 ± 1.74 a	43.46 ± 2.72 c	49.99 ± 6.56 a	6.93 ± 0.61 bc
PA	71.19 ± 0.57 c	17.91 ± 0.14 d	27.89 ± 0.91 b	28.21 ± 0.61 c	43.90 ± 0.45 b	80.24 ± 1.29 a	31.18 ± 0.53 c	70.55 ± 1.62 a

NP, novo-ProD; AL, Alcalase; NZ, novozym; PE, Pepsin; FZ, flavourzyme; PA, papain. Different letters in the same column indicate significant (*p* < 0.05) differences according to Tukey’s test.

**Table 4:** F-test ANOVA performed on all the studied parameters.

	Origin of Enzyme		Proteolytic Character	
	F	*p*	F	*p*
Peptide content	25.11	0.00 *	1.21	0.27
Peptide Yield	9.33	0.00 *	76.73	0.00 *
MW > 5 kDa	111.58	0.00 *	0.28	0.60
5 > MW > 1 kDa	2.31	0.13	66.71	0.00 *
MW < 1 kDa	10.78	0.00 *	16.08	0.00 *
TAA	28.27	0.00 *	16.19	0.00 *
SAA	4.42	0.03 *	22.82	0.00 *
AAA	73.14	0.00 *	8.07	0.01 *
HAA	57.89	0.00 *	17.34	0.00 *
Asp	46.34	0.00 *	12.13	0.00 *
Thr	15.33	0.00 *	15.51	0.00 *
Ser	4.24	0.03 *	7.84	0.01 *
Glu	13.47	0.00 *	114.28	0.00 *
Gly	17.53	0.00 *	58.80	0.00 *
Ala	11.69	0.00 *	22.06	0.00 *
Cys	3.13	0.07	13.02	0.00 *
Val	79.62	0.00 *	17.06	0.00 *
Met	5.20	0.02*	21.07	0.00 *
Ile	85.95	0.00 *	13.71	0.00 *
Leu	55.62	0.00 *	19.34	0.00 *
Tyr	51.74	0.00 *	9.31	0.00 *
Phe	49.31	0.00 *	2.97	0.10
His	26.05	0.00 *	21.93	0.00 *
Lys	46.29	0.00 *	15.30	0.00 *
Arg	29.61	0.00 *	18.50	0.00 *
DPPH	1.81	0.20	7.28	0.02 *
ABTS	0.93	0.42	3.72	0.07
L*	45.10	0.00 *	0.36	0.55
C*_ab_	10.85	0.00 *	0.29	0.60
h_ab_	20.09	0.00 *	7.43	0.01 *

Asterisks denote significant differences at *p* < 0.05. MW, molecular weight; AAA, aromatic amino acids; HAA, hydrophobic amino acids; SAA, sulphur-containing amino acids; TAA, total amino acids.

**Table 5:** Mean and standard derivation of the concentration (mg aa/g hydrolysate) of individual and total soluble amino acids at pH 2 of the peptide hydrolysates of the different enzymes.

	NP	AL	NZ	PE	FZ	PA
Asp	8.93 ± 2.53 b	11.18 ± 0.47 b	11.03 ± 0.28 b	16.36 ± 1.23 a	8.90 ± 0.56 b	4.79 ± 0.77 c
Thr	3.47 ± 0.24 a	3.16 ± 0.89 ab	3.52 ± 0.14 a	4.10 ± 0.80 a	2.89 ± 0.33 ab	1.77 ± 0.29 b
Ser	6.58 ± 0.94 a	7.31 ± 0.61 a	7.34 ± 0.52 a	5.10 ± 3.67 a	4.99 ± 0.48 a	3.56 ± 0.08 a
Glu	52.33 ± 5.36 a	53.18 ± 4.75 a	54.31 ± 3.89 a	54.62 ± 1.94 a	34.97 ± 2.28 b	24.95 ± 0.92 c
Gly	13.06 ± 1.16 ab	11.98 ± 0.93 ab	11.34 ± 0.03 b	13.96 ± 1.32 a	8.57 ± 0.53 c	6.14 ± 0.35 d
Ala	4.79 ± 0.99 abc	5.25 ± 1.14 ab	6.01 ± 1.39 ab	6.97 ± 0.84 a	3.66 ± 0.49 bc	2.51 ± 0.26 c
Cys	0.60 ± 0.07 bc	0.88 ± 0.07 a	0.69 ± 0.04 b	0.61 ± 0.10 bc	0.52 ± 0.04 bc	0.46 ± 0.05 c
Val	6.57 ± 0.73 bc	6.77 ± 0.57 bc	7.59 ± 0.55 b	10.27 ± 0.37 a	5.90 ± 0.44 c	3.06 ± 0.13 d
Met	1.00 ± 0.37 ab	1.64 ± 0.50 a	1.38 ± 0.31 ab	1.69 ± 0.25 a	0.65 ± 0.17 b	0.54 ± 0.14 b
Ile	4.56 ± 0.49 b	4.51 ± 0.52 b	4.94 ± 0.35 b	6.72 ± 0.33 a	4.23 ± 0.36 b	2.33 ± 0.08 c
Leu	7.11 ± 0.80 ab	6.66 ± 1.17 ab	7.35 ± 0.51 b	10.82 ± 0.48 a	5.41 ± 0.57 c	2.99 ± 0.17 d
Tyr	4.23 ± 0.49 b	4.29 ± 0.50 b	4.67 ± 0.33 ab	5.55 ± 0.22 a	4.39 ± 0.33 b	2.63 ± 0.15 c
Phe	4.70 ± 0.51 b	4.54 ± 0.68 b	4.92 ± 0.34 b	7.08 ± 0.33 a	5.73 ± 0.41 b	2.51 ± 0.12 c
His	2.52 ± 0.19 b	2.22 ± 0.23 bc	2.16 ± 0.41 bc	3.25 ± 0.12 a	1.70 ± 0.26 cd	1.27 ± 0.05 d
Lys	3.47 ± 0.25 b	3.80 ± 0.34 b	3.93 ± 0.31 b	4.95 ± 0.07 a	3.33 ± 0.46 b	2.29 ± 0.10 c
Arg	9.62 ± 0.95 b	7.54 ± 2.21 bc	9.28 ± 0.42 bc	13.27 ± 0.62 a	6.52 ± 0.35 cd	4.08 ± 0.18 d
TAA	157.37 ± 16.19 b	155.80 ± 11.78 b	163.43 ± 10.37 ab	187.97 ± 4.10 a	121.14 ± 8.39 c	87.75 ± 4.61 d
SAA	1.60 ± 0.40 abc	2.52 ± 0.57 a	2.06 ± 0.34 ab	2.31 ± 0.35 a	1.17 ± 0.14 bc	1.00 ± 0.17 c
AAA	11.45 ± 1.18 bc	11.06 ± 1.40 bc	12.23 ± 0.97 b	15.87 ± 0.67 a	11.83 ± 0.97 c	6.41 ± 0.33 d
HAA	28.72 ± 3.17 b	29.37 ± 4.52 b	33.02 ± 2.45 b	43.55 ± 1.36 a	25.59 ± 2.15 b	13.95 ± 0.62 c

NP, novo-ProD; AL, alcalase; NZ, novozym; PE, pepsin; FZ, flavourzyme; PA, papain. Asp, aspartic acid; Thr, threonine; Ser, serine; Glu, glutamic acid; Gly, glycine; Ala, alanine; Cys, cysteine; Val, valine; Met, methionine; Ile, isoleucine; Leu, leucine; Tyr, tyrosine; Phe, phenylalanine; His, histidine; Lys, lysine; Arg, arginine; Pro, proline; TAA, total amino acids; SAA, sulphur amino acids; AAA aromatic amino acids; HAA hydrophobic amino acids. Different letters in the same row indicate significant (*p* < 0.05) differences according to Tukey’s test.

**Table 6:** Distribution (%) of the amino acids at the N-terminal and C-terminal positions of the peptide hydrolysates of the different enzymes.

	N-Terminal			C-Terminal		
NP	Q (28.3%)	E (19.6%)	V (7.3%)	R (21.7%)	F (18.2%)	Q (16.67%)
AL	Q (21.7%)	V (8.5%)	S, I (8.5%)	R (19.8%)	L (17.0%)	E (13.21%)
NZ	E (22.2%)	Q (13.9%)	V (12.5%)	Q (22.2%)	R (18.1%)	F (15.28%)
PE	V (11.3%)	L (9.9%)	Q (9.6%)	L (18.9%)	F (12.3%)	Q (9.59%)
FZ	Q (24.6%)	S (10.2%)	E (10.9%)	Q (16.2%)	F (13.4%)	E (10.46%)
PA	Q (18.5%)	A (14.1%)	V (8.3%)	D (14.2%)	Q (13.9%)	R (9.86%)

NP, novo-proD; AL, alcalase; NZ, novozym; PE, pepsin; FZ, flavourzyme; PA, papain. A, alanine; R, arginine; E, glutamic acid; Q, glutamine; I, isoleucine; L, leucine: F, phenylalanine; S, serine; V, valine.

**Table 7:** Mean and standard derivation of antioxidant activity (µmol TE/g sample) using DPPH and ABTS of the peptide hydrolysates of the different enzymes.

	DPPH	ABTS
NP	153.97 ± 6.06 a	23.92 ± 1.43 a
AL	56.19 ± 2.38 c	27.19 ± 1.28 a
NZ	51.18 ± 3.87 c	30.66 ± 3.46 a
PE	81.14 ± 3.60 b	25.96 ± 3.19 a
FZ	44.33 ± 0.46 c	23.54 ± 7.52 a
PA	28.63 ± 5.97 d	22.47 ± 4.44 a

NP, novo-ProD; AL, alcalase; NZ, novozym; PE, pepsin; FZ, flavourzyme; PA, papain. Different letters in the same column indicate significant (*p* < 0.05) differences according to Tukey’s test.

## Data Availability

The original contributions presented in the study are included in the article/Supplementary Material, further inquiries can be directed to the corresponding author.

## Supplementary Materials

The following supporting information can be downloaded at https://www.mdpi.com/article/10.3390/foods14071248/s1. Table S1: Amino acid peptide sequence, mass, length and *m*/*z* of peptide hydrolysates determined by RP-LC-MS/MS.

## Abbreviations

The following abbreviations are used in this manuscript:

NP	Novo-proD enzyme
AL	Alcalase enzyme
NZ	Novozym enzyme
PE	Pepsin enzyme
FZ	Flavourzyme enzyme
PA	Papain enzyme
DGSM	Defatted grape seed meal
MW	Molecular weight
