# Supplementary material for: Obtention and Characterisation of Antioxidant-Rich Peptides from Defatted Grape Seed Meal Using Different Enzymes
Source: Foods. 2025 Apr 3;14(7):1248. doi: 10.3390/foods14071248 (PMC11988616; doi:10.3390/foods14071248)
Supplement: Supplementary file 1 [file foods-14-01248-s001.zip › Rodriguez-Muz_TableS1-Supplementary table.pdf]

Table S1. Amino acid peptide sequence, mass, length and *m/z* of the peptide hydrolysates of the different enzymes determined by RP-LC-MS/MS.

	Accession Number	Peptide sequence	Mass	Length	*m/z*
**NP**	F6HZK2	ANQLDFQPR	1088.525	9	545.2702
		DVSNEANQLDFQPR	1633.7219	14	817.8702
		EANQLDFQPR	1217.5676	10	609.7931
		EGGGSEGRGQESSGDNIF	1781.7452	18	891.8832
		EGRGQESSGDNIF	1394.6062	13	698.3108
		ESSGDNIF	849.3505	8	850.3654
		ESSGDNIFS	936.3824	9	937.3938
		ESSGDNIFSGFDAQQL	1713.7482	16	857.8866
		ESTIGAPGSS	886.4032	10	887.4143
		ESTIGAPGSSR	1060.5149	11	531.2664
		ESTIGAPGSSRS	1147.5469	12	574.782
		ESTIGAPGSSRSE	1276.5895	13	639.3027
		EVEEGDAF	876.3501	8	877.3662
		EVFDHNNEQF	1278.5153	10	640.2674
		EVFDHNNEQFQ	1406.5739	11	704.2975
		GNPQNEFQ	933.3828	8	934.3947
		GQTVANEEVQQGQVL	1598.79	15	800.4041
		IGDPWRADVY	1190.572	10	596.2958
		IREVEEGDAF	1163.5458	10	582.7831
		KIREVEEGDAF	1291.6407	11	646.8294
		LAGNPQNEF	989.4454	9	990.4628
		NEANQLDFQPR	1331.6106	11	666.8133
		NIGDPWRADVY	1304.6149	11	653.3168
		NPQNEFQ	876.3613	7	877.3727
		QALLPPRGQ	961.5345	9	962.5447
		QEGGGSEGRGQ	1043.4268	11	522.7243
		QEGGGSEGRGQESSGDNIF	1892.7772	19	947.3977

Accession Number	Peptide sequence	Mass	Length	*m/z*
	QEGQQGREQEGQ	1355.5702	12	678.7973
	QESSGDNIFSGF	1269.5149	12	635.769
	QESTIGAPGSSRSE	1404.6481	14	703.3318
	QFLGDQHQ	954.4196	8	478.2198
	QGQTVANEEVQQGQVL	1709.822	16	855.9234
	QHCQEGGGSEGRGQ	1428.5801	14	715.3052
	QLDFQPR	885.4344	7	443.7275
	QNIGDPW	811.3500	7	812.3643
	QNIGDPWR	967.4512	8	484.7356
	QQEGGGSEGR	986.4053	10	494.2139
	QQEGGGSEGRGQ	1171.4854	12	586.7531
	QQEGGGSEGRGQE	1300.5280	13	651.2762
	QQGQTVANEEVQQGQVL	1837.8806	17	919.9540
	QQQEGGGSEGR	1114.4639	11	558.2434
	QQQEGGGSEGRGQ	1299.5439	13	650.7837
	QQQQEGGGSEGRGQ	1502.6345	14	752.3218
	QTVANEEVQQGQ	1386.6376	12	694.3281
	RGQESSGDNIF	1208.5421	11	605.2789
	RQESTIGAPGSS	1188.5735	12	595.2978
	RQESTIGAPGSSR	1344.6746	13	673.3485
	STIGAPGSS	775.3712	9	776.3843
	TDENAMIN	923.3542	8	924.3630
	TIEPNGLLLPS	1153.623	11	577.8231
	TIEPNGLLLPSYVNAPQ	1825.9462	17	913.9863
	YTIEPNGLLLPS	1316.6864	12	659.3544
F6HZK3	ANQLDFQPR	1088.525	9	545.2702
	AVPVGTGHF	883.4552	9	884.4645
	DANQLDFQPR	1203.552	10	602.7843
	DANQLDFQPR	1203.552	10	602.7850
	DTSNDANQLDFQPR	1620.7015	14	811.3608

Accession Number	Peptide sequence	Mass	Length	*m/z*
	EGGGSEGRGQESSGDNIF	1781.7452	18	891.8832
	EGRGQESSGDNIF	1394.6062	13	698.3108
	ESSGDNIF	849.3505	8	850.3654
	ESSGDNIFS	936.3824	9	937.3938
	ESSGDNIFSGFDAQQL	1713.7482	16	857.8866
	ESTIAPPGSS	944.4451	10	945.4608
	ESTIAPPGSSR	1082.5356	11	1083.5483
	EVEEGDVF	904.3814	8	905.3928
	EVFDHNNEQF	1278.5153	10	640.2674
	EVFDHNNEQFQ	1406.5739	11	704.2975
	GNPQNEFQ	933.3828	8	934.3947
	IGDPWRADVY	1190.572	10	596.2958
	IREVEEGDVF	1191.5771	10	596.8002
	IYNNGDRQLIV	1306.6405	11	654.326
	KIREVEEGDVF	1319.6721	11	660.8451
	LAGNPQNEF	989.4454	9	990.4628
	NDANQLDFQPR	1317.5948	11	659.8063
	NIGDPWRADVY	1304.6149	11	653.3168
	NPQNEFQ	876.3613	7	877.3727
	QAVLPPR	762.4388	7	763.4491
	QAVLPPRG	819.4603	8	820.4717
	QAVLPPRGQ	947.5189	9	948.5303
	QEGGGSEGRGQ	1043.4268	11	522.7243
	QEGGGSEGRGQESSGDNIF	1892.7772	19	947.3977
	QESSGDNIFSGF	1269.5149	12	635.7690
	QHCQEGGGSEGRGQ	1428.5801	14	715.3052
	QLDFQPR	885.4344	7	443.7275
	QNIGDPW	811.3500	7	812.3643
	QNIGDPWR	967.4512	8	484.7356
	QQEGGGSEGR	986.4053	10	494.2139

Accession Number	Peptide sequence	Mass	Length	*m/z*
	QQEGGGSEGRGQ	1171.4854	12	586.7531
	QQEGGGSEGRGQE	1300.5280	13	651.2762
	QQQEGGGSEGR	1114.4639	11	558.2434
	QQQEGGGSEGRGQ	1299.5439	13	650.7837
	QQQQEGGGSEGRGQ	1502.6345	14	752.3218
	RGQESSGDNIF	1208.5421	11	605.2789
	SNDANQLDFQPR	1405.6110	12	703.8208
	STIAPPGSS	815.4025	9	816.4171
	TSNDANQLDFQPR	1505.6746	13	753.8469
	VLDTSNDAN	948.4036	9	949.4167
	VLDTSNDANQL	1189.5463	11	595.7817
	VLDTSNDANQLDFQPR	1832.854	16	917.4354
	YVNAPQLM	950.4531	8	951.4648
D7U302	ANQLDFQPR	1088.525	9	545.2702
	DANQLDFQPR	1203.552	10	602.7843
	EVQEGDVF	922.3920	8	462.2057
	IREVQEGDVF	1191.5771	10	596.8002
	NDANQLDFQPR	1317.5948	11	659.8063
	QLDFQPR	885.4344	7	443.7275
	QLDFQPR	902.4610	7	452.2415
	SNDANQLDFQPR	1405.6110	12	703.8208
A5C7L5	ERVVPVNPA	961.5345	9	962.5457
	FVDGGSNPKAPII	1312.7139	13	657.3574
	FVDGGSNPKAPIIL	1426.7820	14	714.4031
	GLNAANPISGE	1042.4930	11	1043.5015
	NSMVQPRPV	1001.4600	9	501.7405
	VDGGSNPKAPII	1166.6295	12	584.3237
	VDGGSNPKAPIIL	1261.7030	13	631.8617
Q9M4H7	AAPPTPEPV	877.4545	9	878.4647
	AAPPTPEPVA	948.4916	10	949.5022

	Accession Number	Peptide sequence	Mass	Length	*m/z*
		AEAPADQDETKEVV	1500.6943	14	751.3527
		AEAPADQDETKEVVE	1629.7369	15	815.8781
		AEAPADQDETKEVVEQ	1757.7955	16	879.9081
		PAEEPAPKPEPAPA	1399.6982	14	700.8600
	F6GTY5	EGGGEERQESEGGEHE	1696.6560	16	849.3424
		ENKKQPVEPTEPY	1539.7568	13	770.8895
		KKQPVEPTEPY	1314.6819	11	658.3502
		KQPVEPTEPY	1186.5869	10	594.3016
		QEGGPIIY	858.4123	8	859.4251
	C5DB50	VIVDENGKPVGPIVD	1550.8191	15	776.4181
		VIVDENGKPVGPIVDGD	1722.8676	17	862.4424
	F6GY46	KDKPVDDAVPY	1245.624	11	623.8199
		KPVDDAVPY	1002.5022	9	1003.5138
		VSGYAAETTE	1008.4400	10	505.2318
	F6H3T7	IPPNIEDDHSPLPG	1499.7256	14	750.8732
		VAPDWGVLPM	1099.5372	10	1100.5500
	F6HT88	YGSGGGGGGGGSGGGY	1259.4802	17	630.7432
	F6H813	GSQGGGGGGGGGSGNGK	1246.5286	17	624.2632
	F6GTY8	VYKIGGIGTVP	1102.6387	11	552.3292
	F6HEP7	GFGGGAGVGGGGGLGG	1132.5261	16	567.2711
**AL**	F6HZK3	ANQLDFQPR	1279.6057	11	640.8148
		AVPVGTGHFIY	1834.822	16	918.4185
		DANQLDFQPR	1173.5665	10	587.7930
		DSGFEYVAIK	1088.525	9	545.2715
		DTSNDANQLDFQPR	1622.6696	14	812.3419
		ESSGDNIFSGF	876.3613	7	877.3727
		ESSGDNIFSGFDAQ	883.4552	9	884.4645
		FIYNNGDRQL	889.3430	8	890.3505
		FVQGRGLQGIM	762.4388	7	763.4491

Accession Number	Peptide sequence	Mass	Length	*m/z*
	GDNIFSGFDAQ	1406.5739	11	704.2975
	GQNVFNEEVQQGQVL	1713.7482	16	857.8866
	IKARDSGFE	1208.5421	11	605.2789
	IKARDSGFEY	1317.5948	11	659.8063
	LRAMPLQVISSA	1317.5948	11	659.8071
	NDANQLDFQPR	1318.5790	11	660.2997
	NQLDFQPR	1088.5250	9	545.2702
	QAVLPPRGQ	1191.5771	10	596.8002
	QEGGGSEGRGQE	1203.5520	10	602.7843
	QESSGDNIFSGF	1405.6110	12	703.8208
	QGQNVFNEEVQQGQVL	885.4344	7	443.7275
	QNVFNEEVQQGQVL	922.3920	8	462.2057
	QQEGGGSEGR	902.4610	7	452.2415
	QQEGGGSEGRGQ	1203.5520	10	602.7850
	QQEGGGSEGRGQE	1318.5790	11	660.2981
	QQGQNVFNEEVQQGQVL	1089.5090	9	545.7637
	QQQEGGGSEGR	1088.5250	9	545.2715
	QQQGSEGQ	1166.6295	12	584.3237
	QQQQEGGGSEGR	1261.7030	13	631.8617
	RADVYTPR	1261.7030	13	631.8618
	SNDANQLDFQPR	1426.7820	14	714.4031
	SSGDNIFSGF	1279.7135	13	640.8662
	STIAPPGSSRS	1001.4600	9	501.7405
	STIAPPGSSRSE	1312.7139	13	657.3574
	SVLDTSNDANQL	961.5345	9	962.5457
	VLDTSNDANQL	1280.6975	13	641.3591
	VLDTSNDANQLDFQPR	1042.4930	11	1043.5015
	VNAPQLM	1313.6979	13	657.8574
	VVQQQGQNVFNEE	1629.7369	15	815.8781
	YFVQGRGLQGIM	1500.6943	14	751.3527

Accession Number	Peptide sequence	Mass	Length	*m/z*
	YVNAPQLM	877.4545	9	878.4647
F6HZK2	ANQLDFQPR	1757.7955	16	879.9081
	DSGFEYVAIK	1399.6982	14	700.8600
	DVSNEANQL	948.4916	10	949.5022
	DVSNEANQLDFQPR	1314.6819	11	658.3502
	ESSGDNIFSGF	1315.6659	11	658.8428
	ESSGDNIFSGFDAQ	1696.6560	16	849.3424
	ESTIGAPGSS	858.4123	8	859.4251
	ESTIGAPGSSR	1539.7568	13	770.8895
	GDNIFSGFDAQ	1186.5869	10	594.3016
	GQTVANEEVQQGQVL	1550.8191	15	776.4181
	IKARDSGFE	1722.8676	17	862.4424
	IKARDSGFEY	1245.624	11	623.8199
	IYNNGNRQLVVVS	1002.5022	9	1003.5138
	NEANQLDFQPR	1008.4400	10	505.2318
	NQLDFQPR	1499.7256	14	750.8732
	QEGGGSEGRGQE	1099.5372	10	1100.5500
	QESSGDNIFSGF	1259.4802	17	630.7432
	QKPSNRIQ	1246.5286	17	624.2632
	QQEGGGSEGR	1102.6387	11	552.3292
	QQEGGGSEGRGQ	1102.6387	11	552.3292
	QQEGGGSEGRGQE	1102.6387	11	552.3292
	QQGQTVANEEVQQGQVL	1132.5261	16	567.2711
	QQQEGGGSEGR	1279.6057	11	640.8148
	QQQGSEGQ			
	QQQQEGGGSEGR	1831.8700	16	916.9479
	QVVQQQGQTVANEE	1619.7175	14	810.8710
	RADVYTPR	1171.4854	12	586.7552
	RQESTIGAPGSS	1798.8485	16	900.4368
	RQESTIGAPGSSRSE	1620.7015	14	811.3664

Accession Number	Peptide sequence	Mass	Length	*m/z*
	SSGDNIFSGF	1114.4639	11	558.2452
	STIGAPGSS	1172.4694	12	587.2476
	STIGAPGSSR	1383.6969	12	692.8597
	TAQKPSNRIQSEAGVTE	1687.8165	15	844.9218
	TIEPNGLLLPS	1621.6855	14	811.856
	TVANEEVQQGQVL	1300.7173	12	651.3699
	YTIEPNGLLLPS	1943.9337	17	972.9852
A5C7L5	ANSMVQPRPG	986.4053	10	494.2141
	ERVVPVNPA	1317.5948	11	659.8079
	ERVVPVNPAL	1472.6055	14	737.3149
	FVDGGSNPKAPIIL	1242.5225	12	622.2737
	IPSTDGSSL	1832.854	16	917.4387
	NAANPISGE	1833.838	16	917.9402
	RVVPVNPA	1630.795	14	816.4084
	SIPSTDGSSL	1220.6335	11	611.3264
	TSPLNHGVL	1620.7015	14	811.3619
	VDGGSNPKAP	1832.854	16	917.4387
	VDGGSNPKAPII	1187.5782	12	594.7999
	VDGGSNPKAPIIL	1188.5623	11	595.2909
	VVPVNPAL	1688.8005	15	845.4143
Q9M4H7	AAPPTPEPV	1127.5498	10	564.7854
	AAPPTPEPVA	1709.7985	15	855.9102
	AEAPADQDETKE	1709.7985	15	855.9102
F6H721	GAGAGGGFGGGSGGGAG	1169.4989	11	1170.5149
	GGKGGGGGFGGGAGGGAGGG	1517.7111	13	759.8677
	GKGGGGGFGGGAGGGA	787.3898	7	788.4008
A5AEI7	IRQQAEQQQGGQGD	1058.5356	11	530.2775
A8QK93	INVDQLPGENTLGVS	1088.5250	9	545.2721
C5DB50	FIVDENGKPVGPIVD	1159.6025	11	580.8124
F6HJ38	AAAAAAATGGGGGGGGGKG	950.4531	8	951.4672

	Accession Number	Peptide sequence	Mass	Length	*m/z*
	D7U0V7	IPPNIEDDHSPLPG	951.4371	8	952.4487
	F6GTY5	KKQPVEPTEPY	1275.5942	12	638.8090
	F6HXP6	VAPIPAVPGS	1189.5463	11	1190.5642
	F6GY46	KPVDDAVPY	1926.9071	17	964.4697
	F6HTR0	GGGGGGGGGGGGGGGAASGY	1622.6696	14	812.3461
	A5ASF5	HPHQPQPQQPS	947.5189	9	948.5319
	F6HVJ1	PPVTEPPVE	843.3359	8	844.3547
**NZ**	F6HZK2	AIKTDENAMIN	1234.5863	11	618.2999
		DVSNEANQLDFQPR	1633.7219	14	817.8744
		DVSNEANQLDFQPR	1631.7539	14	816.8911
		ESSGDNIF	849.3505	8	850.3667
		ESSGDNIFSGF	1140.4723	11	1141.4927
		ESSGDNIFSGFDAQ	1472.6055	14	737.3168
		ESTIGAPGSSRSE	1276.5895	13	639.3058
		EVEEGDAF	876.35010	8	877.3651
		EVEEGDAFAVPTGFGH	1660.7368	16	831.3834
		FYLAGNPQNEFQQQ	1682.7688	14	842.3998
		GQTVANEEVQQGQVL	1598.7900	15	800.4070
		NEANQLDFQPR	1331.6106	11	666.8172
		QESSGDNIFSGF	1269.5149	12	635.7695
		QESTIGAPGSSRSE	1404.6481	14	703.3370
		QKPSNRIQ	952.5090	8	477.2672
		QQEGGGSEGR	986.4053	10	494.2149
		QQEGGGSEGRGQ	1171.4854	12	586.7559
		RIQVVQQQGQTVANE	1696.8856	15	849.4443
		STIGAPGSSRSE	1147.5469	12	574.7856
		TDENAMINTLAGNLSL	1691.8036	16	846.9156
		VEGGLQALLPPRGQQE	1690.9001	16	846.4647
		VFDHNNEQF	1149.4727	9	575.7471

Accession Number	Peptide sequence	Mass	Length	*m/z*
	VVQQQGQTVANEE	1428.6844	13	715.3546
	YLAGNPQNEFQQQ	1535.7004	13	768.8639
F6HZK3	DANQLDFQPR	1203.552	10	602.7878
	DTSNDANQLDFQPR	1619.7175	14	810.8730
	ESSGDNIF	849.3505	8	850.3667
	ESSGDNIFSGF	1140.4723	11	1141.4927
	ESSGDNIFSGFDAQ	1472.6055	14	737.3168
	ESTIAPPGSS	926.4345	10	927.4495
	EVEEGDVFAVPVGTGH	1640.7682	16	821.3977
	FYLAGNPQNEFQQQ	1682.7688	14	842.3998
	IQVVQQQGQNVFNEE	1758.8536	15	880.4451
	QESSGDNIFSGF	1269.5149	12	635.7695
	QQEGGGSEGR	986.4053	10	494.2149
	QQEGGGSEGRGQ	1171.4854	12	586.7559
	VFDHNNEQF	1149.4727	9	575.7471
	VLDTSNDANQLDFQPR	1831.8700	16	916.9476
	YLAGNPQNEFQQQ	1535.7004	13	768.8639
A5C7L5	ERVVPVNPA	961.5345	9	962.5498
	FVDGGSNPKAPIIL	1426.7820	14	714.4039
	LHPGIDVSHPL	1183.6349	11	592.8289
	VDGGSNPKAPII	1166.6295	12	584.3264
	VDGGSNPKAPIIL	1261.7003	13	631.8648
Q9M4H7	AAPPTPEPVA	948.4916	10	949.5067
	AEAPADQDETKE	1302.5575	12	652.2925
D7U302	DANQLDFQPR	1203.5520	10	602.7878
	FIYNNGNNRI	1238.6044	10	620.3141
F6H3T7	IPPNIEDDHSPLPG	1499.7256	14	750.8762
F6GTY5	KKQPVEPTEPY	1314.6819	11	658.3535
F6HZK2	AGNPQNEFQ	1004.4199	9	1005.4297
	ANQLDFQPR	1088.5250	9	545.2719

	Accession Number	Peptide sequence	Mass	Length	*m/z*
		DVSNEANQLDFQ	1406.5950	12	704.2958
		DVSNEANQLDFQPR	1633.7219	14	817.8701
		ESTIGAPGSSRSE	1276.5895	13	639.3025
		EVEEGDAF	916.3426	8	917.3521
		EVFDHNNEQFQ	1407.5579	11	704.7878
		FVQGRGLQ	904.4766	8	453.2470
		GRGQESSGDNI	1119.4792	11	560.7471
		HCQQEGGGSEGRGQ	1428.5801	14	715.3021
		ISGCPETF	884.3586	8	443.1794
		NEANQLDFQPR	1331.6106	11	666.8145
		QCHQEGGGSEGRGQ	1428.5801	14	715.3021
		QLDFQPR	885.4344	7	886.4479
	A5C7L5	ERVVPVNPA	961.5345	9	962.5453
		VDGGSNPKAPII	1288.6064	12	645.3041
		VDGGSNPKAPIIL	1261.7030	13	631.8591
	F6GTY5	KKQPVEPTEPY	1314.6819	11	658.3474
	F6HNA7	GGGGGGGGGGGGGGGGGG	1044.3969	18	523.202
	F6I111	GGGGGGGGGGCY	854.2977	12	428.1494
	F6GWM3	GGGGGGGCYN	798.2603	10	400.1325
	A5AEI7	IVQRQQGQGQ	1142.5680	10	572.2948
**PE**	F6HZK2	AEAFNVDVQL	1105.5292	10	1106.5444
		AFAVPTGF	808.4119	8	809.4288
		AFAVPTGFG	865.4333	9	866.4476
		AFAVPTGFGH	1002.4923	10	1003.5065
		AFAVPTGFGHY	1165.5557	11	1166.5731
		AFAVPTGFGHYI	1278.6396	12	640.3317
		AFAVPTGFGHYIY	1441.703	13	721.8647
		AFAVPTGFGHYIYNNG	1727.7943	16	864.9069
		AFAVPTGFGHYIYNNGN	1842.8213	17	922.4292

Accession Number	Peptide sequence	Mass	Length	*m/z*
	AGVTEVFDH	973.4505	9	487.7368
	AGVTEVFDHNNE	1330.579	12	666.3012
	AGVTEVFDHNNEQ	1458.6375	13	730.3305
	ALIKARDSGF	1076.5978	10	539.3097
	ALIKARDSGFEY	1368.7037	12	685.3630
	ALLPPRGQQE	1107.6036	10	554.8133
	ALLPPRGQQERGEQ	1577.8274	14	789.9258
	ALLPPRGQQERGEQQ	1705.8860	15	853.9559
	ALLPPRGQQERGEQQQ	1833.9445	16	917.9856
	ALVLPYYNVNA	1235.6550	11	618.8391
	AMINTLAGNL	1016.5325	10	509.2768
	AMINTLAGNLSL	1216.6486	12	609.3359
	AMPVQVIASA	985.5266	10	986.5384
	ANQLDFQPR	1088.5250	9	545.2720
	AVPTGFGH	784.3868	8	785.4008
	AVPTGFGHY	947.4501	9	948.4645
	AVPTGFGHYI	1060.5342	10	531.2778
	AVPTGFGHYIY	1223.5974	11	612.8113
	AVPTGFGHYIYNN	1451.6833	13	726.8546
	AVPTGFGHYIYNNG	1508.7048	14	755.3647
	AVPTGFGHYIYNNGN	1622.7477	15	812.3867
	AVPTGFGHYIYNNGNRQ	1907.8915	17	954.9583
	AVVRYTIEPNGLLL	1599.8984	14	800.9559
	AYQASNNEAKQL	1335.6418	12	668.8324
	AYQASNNEAKQLKH	1600.7958	14	801.4105
	AYQASNNEAKQLKHN	1714.8386	15	858.4325
	AYQASNNEAKQLKHNR	1870.9398	16	936.4841
	DAFAVPTGF	923.4388	9	924.4513
	DAFAVPTGFGH	1117.5192	11	559.7701
	DAFAVPTGFGHY	1280.5825	12	641.3024

Accession Number	Peptide sequence	Mass	Length	*m/z*
	DAQQLAEAF	1013.443	9	507.7322
	DAQQLAEAF	991.4611	9	496.7419
	DAQQLAEAFN	1105.5039	10	1106.519
	DNIFSGF	798.3548	7	799.3723
	DNIFSGFD	913.3817	8	914.3954
	DNIFSGFDAQQL	1353.6201	12	677.8248
	DVSNEANQ	875.3621	8	438.6935
	DVSNEANQLD	1103.473	10	552.7463
	DVSNEANQLDF	1250.5415	11	626.2815
	DVSNEANQLDFQPR	1631.7539	14	816.8895
	DVSNEANQLDFQPRR	1788.839	15	895.4329
	DVSNEANQLDFQPRRF	1935.9075	16	646.3137
	EETICSLRL	1062.538	9	532.2799
	EEVQQGQVLVIPQNF	1726.8889	15	864.4572
	EGGGSEGRGQESSGD	1389.5392	15	695.783
	EGGGSEGRGQESSGDN	1503.5822	16	752.8126
	EGGGSEGRGQESSGDNIF	1781.7452	18	891.8856
	EGGGSEGRGQESSGDNIFSG	1925.7987	20	963.9147
	EGRGQESSGDNIF	1394.6062	13	698.3151
	EGRGQESSGDNIFSGF	1667.7175	16	834.8716
	ENAMINTLAGNL	1275.6129	12	638.819
	ENAMINTLAGNLSL	1475.729	14	738.8749
	ESSGDNIFSGF	1158.4829	11	580.251
	EVFDHNNEQ	1112.4523	9	1113.4596
	EVQQGQVL	899.4713	8	450.7473
	EVQQGQVLVIPQNF	1598.8304	14	800.4277
	EYAMINTLAGNLSL	1508.7545	14	755.3824
	EYVAIKT	822.4487	7	823.4616
	EYVAIKTD	937.4756	8	938.4907
	EYVAIKTDE	1066.5182	9	534.2721

Accession Number	Peptide sequence	Mass	Length	*m/z*
	EYVAIKTDENA	1251.5983	11	1252.6099
	FAVPTGF	737.3748	7	738.3895
	FAVPTGFG	794.3962	8	795.4135
	FAVPTGFGH	931.4552	9	932.47
	FAVPTGFGHY	1094.5186	10	1095.5361
	FAVPTGFGHYI	1207.6025	11	604.8152
	FAVPTGFGHYIY	1370.6659	12	686.3463
	FAVPTGFGHYIYN	1484.7089	13	743.3661
	FAVPTGFGHYIYNN	1600.7197	14	801.3749
	FAVPTGFGHYIYNNG	1655.7732	15	828.9003
	FAVPTGFGHYIYNNGN	1769.8162	16	885.9197
	FAVPTGFGHYIYNNGNR	1926.9012	17	964.4622
	FAVPTGYGHY	1110.5134	10	556.2672
	FDHNNEQF	1049.4203	8	525.7213
	FFAVPTGFGH	1078.5236	10	540.2729
	FFAVPTGFGHYIY	1517.7343	13	759.8824
	FLGDQHQ	843.3875	7	844.4007
	FLGDQHQKI	1084.5665	9	1085.5824
	FLGDQHQKIR	1240.6676	10	621.3444
	FQESQEGQQGR	1292.5745	11	647.2965
	FQQQQQQQEQQQG	1759.7874	14	880.9069
	FQQQQQQQEQQQGSE	1975.8619	16	988.943
	FVQGRGLQ	903.4926	8	452.7579
	FVQGRGLQG	960.5141	9	481.2688
	FVQGRGLQGI	1073.5981	10	537.8103
	FVQGRGLQGIL	1186.6823	11	594.3542
	FVQGRGLQGILI	1300.7502	12	651.3864
	FYLAGNPQN	1022.4821	9	512.2514
	FYLAGNPQNE	1151.5247	10	1152.5403
	FYLAGNPQNEF	1298.5931	11	650.3088

Accession Number	Peptide sequence	Mass	Length	*m/z*
	FYLAGNPQNEFQ	1426.6517	12	714.3373
	FYLAGNPQNEFQQ	1554.7102	13	778.381
	FYLAGNPQNEFQQQ	1682.7688	14	842.3978
	FYLAGNPQNEFQQQQ	1810.8274	15	906.4254
	GGSEGRGQESSGDNIF	1595.6812	16	798.8534
	GGSEGRGQESSGDNIFSGF	1886.803	19	944.4139
	GHRSSVTGYDL	1190.568	11	596.2947
	GHYIYNNG	936.4089	8	937.4238
	GHYIYNNGNR	1207.537	10	604.7792
	GHYIYNNGNRQ	1335.5956	11	668.8094
	GHYIYNNGNRQL	1447.6957	12	724.8604
	GHYIYNNGNRQLV	1546.764	13	774.3896
	GHYIYNNGNRQLVV	1646.8165	14	824.421
	GLLLPSYVNAPQL	1383.7761	13	692.9012
	GLLLPSYVNAPQLL	1496.8602	14	749.4435
	GNIVRVEGGL	1012.5665	10	507.2941
	GSEGRGQESSGDNIFSGF	1829.7816	18	915.9028
	GVTEVFDH	902.4134	8	452.2171
	GVTEVFDHNN	1130.4993	10	566.2609
	GVTEVFDHNNE	1259.5419	11	630.7814
	GVTEVFDHNNEQ	1387.6003	12	694.8123
	GYEETICSLRLKQNIGD	1903.9639	17	952.993
	HNRQESTIGAPGSS	1439.6753	14	720.848
	HNRQESTIGAPGSSR	1595.7764	15	532.9348
	HNRQESTIGAPGSSRSE	1811.8511	17	906.94
	HRSSVTGYDL	1133.5465	10	567.784
	HRSSVTGYDLPVL	1442.7517	13	722.3871
	IASAYQASNN	1037.4778	10	519.749
	IASAYQASNNE	1166.5204	11	584.265
	IASAYQASNNEAKQ	1493.7109	14	747.8655

Accession Number	Peptide sequence	Mass	Length	*m/z*
	IASAYQASNNEAKQL	1606.795	15	804.4094
	IFSGFDAQQ	1011.4661	9	506.7437
	IFSGFDAQQL	1125.5342	10	563.777
	IGAPGSSRSE	959.4672	10	960.481
	IGDPWRAD	928.4402	8	465.2303
	IGDPWRADV	1027.5087	9	514.7665
	IGDPWRADVY	1190.572	10	596.2979
	IKARDSGF	892.4766	8	447.2506
	IKARDSGFE	1021.5192	9	511.771
	IKARDSGFEY	1184.5825	10	593.3036
	IKTDENAM	920.4273	8	461.2247
	IKTDENAMIN	1163.5492	10	582.7857
	IKTDENAMINT	1264.5969	11	633.3105
	IKTDENAMINTL	1377.681	12	689.8528
	IKTDENAMINTLAGNL	1732.8666	16	867.4442
	IKTDENAMINTLAGNLSL	1932.9827	18	967.5048
	INTLAGNL	814.4548	8	408.2385
	INTLAGNLS	901.4869	9	902.501
	INTLAGNLSL	1014.5709	10	508.2972
	IQSEAGVTE	932.4451	9	933.4644
	IQSEAGVTEV	1031.5134	10	1032.5345
	IQSEAGVTEVF	1200.5638	11	601.2939
	IQSEAGVTEVFDH	1430.6677	13	716.3453
	IQSEAGVTEVFDHNN	1658.7537	15	830.3896
	IQSEAGVTEVFDHNNE	1788.7802	16	895.402
	IQSEAGVTEVFDHNNEQ	1915.8547	17	958.9445
	IQVVQQQGQTVA	1297.699	12	1298.7107
	IQVVQQQGQTVAN	1411.7419	13	706.8825
	IQVVQQQGQTVANE	1541.7686	14	771.8959
	IQVVQQQGQTVANEE	1669.8271	15	835.9253

Accession Number	Peptide sequence	Mass	Length	*m/z*
	ISGCPETFQSF	1214.5277	11	1215.5531
	IVRVEGGL	841.5021	8	421.7634
	IVRVEGGLQ	969.5607	9	485.7919
	IVRVEGGLQA	1040.5978	10	521.3095
	IYNNGNRQL	1090.552	9	546.2886
	IYNNGNRQLV	1190.6044	10	596.313
	IYNNGNRQLVV	1289.6727	11	645.8476
	KLQGQNDRRGNIVR	1652.9182	14	551.9848
	KQNIGDPW	956.4716	8	479.2458
	KQNIGDPWR	1112.5726	9	557.2992
	KQNIGDPWRA	1183.6097	10	592.8159
	KQNIGDPWRAD	1298.6367	11	433.8893
	KQNIGDPWRADV	1379.6946	12	690.8586
	KQNIGDPWRADVF	1544.7736	13	773.3997
	LAEAFNVDVQ	1105.5292	10	1106.5437
	LAGNPQNE	841.3929	8	842.4092
	LAGNPQNEF	988.4614	9	495.2422
	LDFQPRR	930.5035	7	466.263
	LDVSNEANQ	988.4462	9	495.2334
	LDVSNEANQL	1101.5302	10	551.7768
	LDVSNEANQLDF	1363.6255	12	682.8243
	LDVSNEANQLDFQPR	1744.838	15	873.4342
	LDVSNEANQLDFQPRR	1900.9391	16	951.483
	LFAVPTGFGHYIY	1483.75	13	742.8849
	LGDQHQKIRE	1222.6418	10	612.3301
	LIKARDSGF	1005.5607	9	503.7909
	LIKARDSGFE	1134.6033	10	568.3119
	LIKARDSGFEY	1297.6666	11	649.8446
	LINTLAGNLSL	1127.655	11	564.8393
	LIRKLQGQND	1183.6672	10	592.8446

Accession Number	Peptide sequence	Mass	Length	*m/z*
	LIRKLQGQNDR	1339.7684	11	447.6006
	LLLPSYVNA	988.5593	9	495.2905
	LLLPSYVNAPQ	1213.6707	11	607.8467
	LLLPSYVNAPQL	1326.7546	12	664.3914
	LLLPSYVNAPQLL	1439.8387	13	720.9326
	LLLPSYVNAPQLLY	1602.9021	14	802.4636
	LLPSYVNAPQL	1213.6707	11	607.8468
	LLPSYVNAPQLL	1326.7546	12	664.389
	LLVLPYYNVNA	1277.7019	11	1278.7168
	LMINTLAGNL	1058.5795	10	530.3006
	LPSYVNAPQ	987.5025	9	494.7619
	LPSYVNAPQLL	1213.6707	11	1214.6869
	LPSYVNAPQLLY	1376.734	12	1377.7458
	LQGQNDRRGNIVR	1524.8232	13	763.424
	LRLKQNIGDPW	1338.7407	11	670.3824
	LRLKQNIGDPWR	1494.8419	12	499.291
	LRLKQNIGDPWRAD	1680.9059	14	561.312
	LVIPQNF	829.4698	7	415.7468
	LVKLSAHKGRLYQ	1511.8936	13	504.9754
	LVLPYYNV	979.5378	8	490.7794
	LVLPYYNVN	1093.5808	9	547.8024
	LVLPYYNVNA	1164.6179	10	583.3223
	LVLPYYNVNAN	1278.6608	11	640.3416
	LVLPYYNVNANS	1365.6929	12	683.8587
	LVLPYYNVNANSV	1464.7612	13	733.3932
	LVLPYYNVNANSVI	1577.8453	14	789.9366
	LVLPYYNVNANSVIY	1741.8927	15	871.9639
	LYFVQGR	881.4759	7	441.7494
	LYFVQGRGL	1051.5814	9	526.8008
	LYFVQGRGLQ	1179.64	10	590.8309

Accession Number	Peptide sequence	Mass	Length	*m/z*
	LYFVQGRGLQG	1236.6615	11	619.3448
	LYFVQGRGLQGI	1349.7455	12	675.8853
	LYFVQGRGLQGIL	1462.8296	13	732.4279
	LYFVQGRGLQGILY	1625.8929	14	813.9598
	LYLAGNPQNEF	1264.6088	11	633.3174
	LYLAGNPQNEFQ	1392.6674	12	697.3459
	LYLAGNPQNEFQQQQ	1776.843	15	889.4347
	MINTLAGN	832.4113	8	833.4265
	MINTLAGNL	961.4903	9	481.7558
	MINTLAGNLS	1014.5168	10	508.2687
	MINTLAGNLSL	1161.6063	11	581.8168
	MRAMPVQ	831.4095	7	416.7167
	MRAMPVQV	946.4728	8	474.2493
	MRAMPVQVIASA	1272.6682	12	637.3452
	NAMINTLAGN	1017.4913	10	509.7565
	NAMINTLAGNL	1130.5754	11	566.2993
	NAMINTLAGNLS	1217.6074	12	609.8158
	NAMINTLAGNLSL	1330.6915	13	666.3569
	NEEVQQGQV	1029.4727	9	1030.4875
	NEEVQQGQVL	1142.5568	10	572.2913
	NIFSGFD	798.3548	7	799.3682
	NIFSGFDA	869.3919	8	870.4048
	NIFSGFDAQ	997.4505	9	998.4671
	NIFSGFDAQQ	1126.493	10	1127.5128
	NIFSGFDAQQL	1238.5931	11	1239.6064
	NIFSGFDAQQLAE	1438.6729	13	720.3492
	NIVRVEGGL	955.545	9	478.7834
	NIVRVEGGLQ	1083.6036	10	542.8127
	NNGNRQLVV	1012.5414	9	1013.5547
	NRIQSEAGVTE	1202.5891	11	602.3058

Accession Number	Peptide sequence	Mass	Length	*m/z*
	NRQESTIGAPGSSR	1458.7175	14	730.3705
	NRQESTIGAPGSSRS	1545.7495	15	773.8862
	NRQESTIGAPGSSRSE	1675.7761	16	838.8997
	NTLAGNLSL	901.4869	9	902.4999
	NVDVQLIR	955.545	8	478.7834
	NVNANSVIY	992.4927	9	993.5066
	PSNRIQSEAGVTEV	1485.7423	14	743.8863
	PSYVNAPQL	987.5025	9	988.522
	PSYVNAPQLL	1100.5865	10	1101.6082
	PSYVNAPQLLY	1263.6499	11	632.8397
	PTGFGHY	777.3445	7	778.3561
	PTGFGHYI	890.4286	8	891.4438
	PYYNVNA	839.3813	7	840.3976
	PYYNVNANS	1040.4563	9	1041.4706
	PYYNVNANSV	1139.5247	10	1140.5389
	PYYNVNANSVI	1252.6088	11	627.3155
	PYYNVNANSVIY	1415.6721	12	708.8494
	QALLPPRGQQE	1218.6356	11	1219.6482
	QALLPPRGQQER	1374.7367	12	688.3823
	QALLPPRGQQERG	1431.7582	13	716.8925
	QALLPPRGQQERGE	1560.8008	14	781.4133
	QALLPPRGQQERGEQ	1688.8594	15	845.4421
	QALLPPRGQQERGEQQ	1816.918	16	909.4733
	QALLPPRGQQERGEQQQ	1944.9766	17	973.5038
	QASNNEAKQLKHNR	1619.8127	14	810.9188
	QEGGGSEGRGQES	1259.5013	13	630.7628
	QEGGGSEGRGQESSG	1403.5549	15	702.7905
	QEGGGSEGRGQESSGD	1518.5818	16	760.3035
	QEGGGSEGRGQESSGDN	1632.6248	17	817.3242
	QEGGGSEGRGQESSGDNIF	1892.7772	19	947.4014

Accession Number	Peptide sequence	Mass	Length	*m/z*
	QESQEGQQGR	1128.4796	10	565.2535
	QESQEGQQGRE	1257.5221	11	629.7723
	QESQEGQQGREQ	1385.5807	12	693.8022
	QESQEGQQGREQEGQ	1716.7299	15	859.3817
	QESQEGQQGREQEGQQ	1844.7885	16	923.4106
	QESQEGQQGREQEGQQG	1884.7834	17	943.4019
	QESSGDNIF	978.393	9	979.4058
	QESSGDNIFSGF	1269.5149	12	635.7712
	QESSGDNIFSGFDAQQ	1710.7122	16	856.3657
	QESSGDNIFSGFDAQQL	1824.7802	17	913.4102
	QESTIGAPGSS	1033.4563	11	1034.4688
	QESTIGAPGSSR	1188.5735	12	595.2979
	QESTIGAPGSSRS	1275.6055	13	638.8152
	QESTIGAPGSSRSE	1404.6481	14	703.338
	QFLGDQHQKIR	1351.6997	11	676.8624
	QFLGDQHQKIRE	1480.7422	12	741.3829
	QGQQFLGDQHQK	1395.6531	12	698.8384
	QGQQFLGDQHQKI	1508.7372	13	755.3804
	QGQQFLGDQHQKIR	1664.8383	14	833.4307
	QGQQFLGDQHQKIRE	1793.8809	15	897.9538
	QGQQGQQFLGDQHQK	1708.7917	15	855.4089
	QGQQGQQFLGDQHQKI	1821.8757	16	911.9509
	QKLVKLSAHKGR	1346.8146	12	449.9483
	QKLVKLSAHKGRL	1459.8987	13	487.6434
	QQERGEQQQD	1227.5116	10	614.7698
	QQFLGDQHQK	1210.5731	10	606.2974
	QQFLGDQHQKI	1323.6571	11	662.8403
	QQFLGDQHQKIR	1479.7582	12	494.263
	QQFLGDQHQKIRE	1608.8008	13	537.2789
	QQGQQFLGDQHQK	1523.7117	13	762.8677

Accession Number	Peptide sequence	Mass	Length	*m/z*
	QQGQQFLGDQHQKI	1636.7958	14	819.4092
	QQGQQFLGDQHQKIR	1792.8969	15	897.4618
	QQGQQFLGDQHQKIRE	1921.9395	16	961.984
	QQGQQGQQFLGDQHQK	1836.8503	16	919.4395
	QQGQQGQQFLGDQHQKI	1949.9343	17	975.9802
	QQPAQGQQFLGDQHQK	1837.8707	16	919.9305
	QQQEGGGSEGRGQE	1428.5865	14	715.3049
	QQQEGGGSEGRGQESSGDN	1888.7419	19	945.3696
	QQQQEQQQGSEGQQQ	1757.7565	15	879.896
	QSFQESQEGQQGR	1507.6652	13	754.8408
	QSFQESQEGQQGREQE	1893.8088	16	947.9175
	QVLVIPQNF	1056.5968	9	529.3093
	QVVQQQGQTVANE	1427.7004	13	714.8633
	QVVQQQGQTVANEE	1556.743	14	779.3854
	RAMPVQVIASA	1141.6277	11	1142.644
	RFYLAGNPQ	1064.5403	9	533.2808
	RFYLAGNPQN	1178.5833	10	590.3024
	RFYLAGNPQNE	1307.6259	11	654.8275
	RFYLAGNPQNEF	1454.6942	12	728.358
	RFYLAGNPQNEFQ	1583.7368	13	792.8866
	RFYLAGNPQNEFQQQ	1839.854	15	920.9435
	RGQESSGDNIF	1208.5421	11	1209.5574
	RGQESSGDNIFSGF	1499.6641	14	750.8441
	RIQSEAGV	858.4559	8	430.2385
	RIQSEAGVTE	1088.5461	10	545.2859
	RIQSEAGVTEV	1187.6146	11	1188.6307
	RIQSEAGVTEVF	1334.683	12	668.3531
	RIQVVQQ	869.5083	7	435.7661
	RIQVVQQQ	997.5669	8	499.7942
	RIQVVQQQG	1054.5884	9	528.3048

**Accession Number**	**Peptide sequence**	**Mass**	**Length**	***m/z***
	RIQVVQQQGQ	1183.6309	10	592.8268
	RIQVVQQQGQT	1283.6946	11	642.8594
	RIQVVQQQGQTVA	1453.8	13	727.9119
	RIQVVQQQGQTVAN	1567.843	14	784.9357
	RIQVVQQQGQTVANE	1696.8856	15	849.4601
	RIQVVQQQGQTVANEE	1825.9282	16	913.9781
	RIQVVQQQGQTVANEL	1809.9697	16	905.9979
	RLKQNIGDPWRAD	1567.8219	13	523.6182
	RLKQNIGDPWRADV	1666.8903	14	556.6412
	RQESTIGAPGSSR	1344.6746	13	673.3486
	RQESTIGAPGSSRSE	1560.7491	15	781.3835
	RSSVTGYDLPVL	1305.6929	12	653.8583
	RYTIEPNGLL	1174.6346	10	588.3286
	RYTIEPNGLLL	1288.7026	11	645.3636
	SARIQVVQQQGQTVANE	1854.9547	17	928.4927
	SAYQASNNEAKQL	1422.6738	13	712.3494
	SAYQASNNEAKQLK	1550.7688	14	776.3948
	SAYQASNNEAKQLKH	1687.8278	15	844.9266
	SAYQASNNEAKQLKHN	1801.8707	16	901.948
	SAYQASNNEAKQLKHNR	1957.9718	17	979.9988
	SEAGVTEV	790.3708	8	791.3845
	SEAGVTEVFDHNNEQ	1674.7122	15	838.3695
	SEGQQQQQEGGGSEGRG	1718.7091	17	860.3733
	SEGQQQQQEGGGSEGRGQ	1845.7837	18	923.9127
	SEGQQQQQEGGGSEGRGQE	1974.8263	19	988.4324
	SEGRGQESSGDNI	1334.5698	13	668.2959
	SEGRGQESSGDNIF	1482.6222	14	742.3239
	SEGRGQESSGDNIFSG	1625.6917	16	813.858
	SEGRGQESSGDNIFSGF	1772.7601	17	887.3926
	SGDNIFSGFDAQQL	1497.6736	14	749.8507

Accession Number	Peptide sequence	Mass	Length	*m/z*
	SGFDAQQL	864.3977	8	865.4114
	SGFDAQQLA	935.4348	9	936.448
	SLMRAMPVQ	1047.5205	9	524.7708
	SLMRAMPVQV	1130.594	10	566.3074
	SLMRAMPVQVIASA	1488.7793	14	745.4017
	SSGDNIFSGF	1030.4243	10	1031.4281
	SSVTGYD	727.3024	7	728.3135
	SSVTGYDL	840.3865	8	841.3987
	STIGAPGSS	775.3712	9	776.3828
	STIGAPGSSRS	1018.5043	11	510.2631
	STIGAPGSSRSE	1147.5469	12	574.787
	SVLDVSNEANQL	1287.6306	12	644.8263
	SYVNAPQL	890.4498	8	891.4619
	SYVNAPQLL	1003.5338	9	1004.5486
	TAQKPSNRIQSE	1358.679	12	680.3508
	TAQKPSNRIQSEA	1428.7321	13	715.3782
	TAQKPSNRIQSEAG	1485.7535	14	743.8882
	TAQKPSNRIQSEAGV	1584.8219	15	793.4242
	TAQKPSNRIQSEAGVT	1685.8696	16	843.9478
	TAQKPSNRIQSEAGVTE	1815.8962	17	908.9625
	TAQKPSNRIQSEAGVTEV	1913.9806	18	958.0052
	TDENAMINTLAGNL	1491.6875	14	746.8574
	TDENAMINTLAGNLSL	1691.8036	16	846.9139
	TEVFDHNNE	1103.4519	9	552.7366
	TIEPNGLL	855.4702	8	428.7454
	TIEPNGLLL	968.5542	9	485.2895
	TIEPNGLLLPSY	1337.6843	12	669.8549
	TIEPNGLLLPSYVN	1528.8137	14	765.4114
	TIEPNGLLLPSYVNA	1599.8508	15	800.9384
	TIEPNGLLLPSYVNAPQL	1939.0302	18	970.5327

Accession Number	Peptide sequence	Mass	Length	*m/z*
	TIGAPGSSRS	931.4723	10	466.7475
	TIGAPGSSRSE	1060.5149	11	531.267
	VAIKTDEN	888.4553	8	445.2396
	VAIKTDENA	959.4924	9	960.5043
	VAIKTDENAM	1106.5277	10	554.2761
	VAIKTDENAMI	1219.6118	11	610.8176
	VAIKTDENAMIN	1333.6548	12	667.8376
	VAIKTDENAMINT	1434.7024	13	718.3626
	VAIKTDENAMINTL	1548.7705	14	775.3973
	VAIKTDENAMINTLAGNL	1902.972	18	952.5
	VEEGDAF	765.3181	7	766.3317
	VEEGDAFAVPTGF	1337.6139	13	669.8188
	VEEGDAFAVPTGFGHY	1694.7576	16	848.3917
	VEEGDAFAVPTGFGHYIY	1970.905	18	986.4706
	VFDHNNEQ	1001.4203	8	501.7231
	VFDHNNEQF	1148.4886	9	1149.5026
	VIASAYQASNN	1136.5461	11	569.2828
	VIASAYQASNNEAK	1464.7208	14	733.3725
	VIASAYQASNNEAKQL	1706.8475	16	854.4331
	VKLSAHKGRL	1107.6876	10	554.8541
	VKLSAHKGRLY	1270.751	11	636.3856
	VKLSAHKGRLYQG	1455.8309	13	728.9268
	VKLSAHKGRLYQGA	1526.868	14	764.4452
	VKLSAHKGRLYQGAL	1639.9521	15	820.9898
	VLDVSNEANQ	1087.5145	10	544.7671
	VLDVSNEANQL	1200.5986	11	601.3121
	VLDVSNEANQLD	1315.6255	12	658.8259
	VLDVSNEANQLDFQPR	1843.9064	16	922.9663
	VLPYYNV	866.4538	7	867.4714
	VLPYYNVNA	1051.5338	9	526.779

Accession Number	Peptide sequence	Mass	Length	*m/z*
	VLPYYNVNANS	1252.6088	11	627.3163
	VLPYYNVNANSVI	1464.7612	13	733.3932
	VLVIPQNF	928.5382	8	465.2792
	VNAPQLLY	916.5018	8	459.2615
	VPTGFGHY	876.413	8	439.2169
	VPTGFGHYIY	1152.5603	10	577.29
	VQGRGLQGIL	1040.5978	10	521.3079
	VQGRGLQGILI	1152.6979	11	577.3604
	VQQGQVLVIPQNF	1468.8038	13	735.4142
	VQQQGQTVANE	1200.5735	11	601.2974
	VRVEGGLQ	856.4766	8	857.4908
	VRYTIEPNG	1047.5349	9	524.7792
	VRYTIEPNGL	1160.6189	10	581.3219
	VRYTIEPNGLL	1273.703	11	637.866
	VRYTIEPNGLLL	1386.7871	12	694.4067
	VRYTIEPNGLLLPS	1570.8718	14	786.446
	VRYTIEPNGLLLPSY	1733.9352	15	867.9816
	VSNEANQLDFQPR	1516.7269	13	759.3756
	VSNEANQLDFQPRRF	1819.8965	15	910.9594
	VSVLDVSNEANQL	1386.6991	13	1387.7146
	VTEVFDHNN	1073.4778	9	537.7493
	VTEVFDHNNE	1202.5204	10	602.2723
	VTEVFDHNNEQ	1330.579	11	666.3029
	VTEVFDHNNEQF	1477.6473	12	739.8337
	VVQQQGQTVA	1056.5564	10	1057.5712
	VVQQQGQTVAN	1170.5992	11	586.3102
	VVQQQGQTVANE	1299.6418	12	650.8347
	VVQQQGQTVANEE	1429.6685	13	715.8456
	VVRYTIEPNGLLL	1629.8977	13	815.9615
	VYTPRGGHRSSVTG	1472.7484	14	737.3853

Accession Number	Peptide sequence	Mass	Length	*m/z*
	VYTPRGGHRSSVTGY	1635.8118	15	818.9189
	VYTPRGGHRSSVTGYD	1750.8386	16	584.6255
	VYTPRGGHRSSVTGYDL	1845.9121	17	923.9677
	YAIRGSARIQ	1133.6305	10	567.8259
	YEETICSLR	1078.5294	9	540.2754
	YEETICSLRL	1191.6135	10	596.8203
	YEETICSLRLKQNIGD	1846.9425	16	924.4846
	YFVQGRGL	938.4974	8	470.2601
	YFVQGRGLQ	1066.5559	9	534.2892
	YFVQGRGLQG	1123.5774	10	562.8019
	YFVQGRGLQGI	1236.6615	11	619.3427
	YFVQGRGLQGIL	1350.7295	12	676.3768
	YFVQGRGLQGILI	1462.8296	13	732.4279
	YIYNNGNR	1012.4727	8	507.2472
	YIYNNGNRQ	1140.5312	9	571.2794
	YIYNNGNRQL	1253.6152	10	627.8219
	YIYNNGNRQLV	1353.6677	11	677.8441
	YIYNNGNRQLVV	1452.7361	12	727.3824
	YIYNNGNRQLVVV	1551.8046	13	776.917
	YLAGNPQ	761.3708	7	762.3851
	YLAGNPQN	875.4137	8	438.7178
	YLAGNPQNE	1004.4563	9	503.2408
	YLAGNPQNEF	1151.5247	10	576.776
	YLAGNPQNEFQ	1279.5833	11	640.8041
	YLAGNPQNEFQQ	1407.6418	12	704.8329
	YLAGNPQNEFQQQ	1535.7004	13	768.8635
	YLAGNPQNEFQQQQ	1663.759	14	832.8923
	YLAGNPQNEFQQQQQ	1792.8016	15	897.4148
	YLAGNPQNEFQQQQQQ	1920.8602	16	961.4449
	YQASNNEAKQL	1264.6047	11	633.3166

Accession Number	Peptide sequence	Mass	Length	*m/z*
	YQASNNEAKQLK	1392.6997	12	697.36
	YQASNNEAKQLKH	1529.7587	13	765.8909
	YQASNNEAKQLKHN	1643.8015	14	822.913
	YQASNNEAKQLKHNR	1799.9027	15	900.9635
	YQASNNEAKQLKHNRQ	1927.9612	16	643.6639
	YTIEPNG	792.3654	7	793.379
	YTIEPNGL	906.4334	8	907.447
	YTIEPNGLL	1018.5335	9	510.2776
	YTIEPNGLLLPS	1315.7024	12	658.8619
	YTIEPNGLLLPSY	1479.7496	13	740.8865
	YTIEPNGLLLPSYVNA	1762.9141	16	882.4707
	YTPRGGHRSSVTGY	1536.7433	14	769.3839
	YTPRGGHRSSVTGYD	1651.7703	15	826.896
	YVAIKTDEN	1051.5186	9	1052.5347
	YVAIKTDENA	1122.5557	10	1123.571
	YVAIKTDENAM	1269.5911	11	635.8074
	YVAIKTDENAMINTL	1711.8339	15	856.9318
	YVNAPQL	803.4177	7	804.4307
	YVNAPQLL	916.5018	8	917.5161
F6HZK3	AEAFNVDVQL	1105.5292	10	1106.5444
	AGVTEVFDH	973.4505	9	487.7368
	AGVTEVFDHNNE	1330.579	12	666.3012
	AGVTEVFDHNNEQ	1458.6375	13	730.3305
	ALIKARDSGF	1076.5978	10	539.3097
	ALIKARDSGFEY	1368.7037	12	685.363
	AMINTLAGNL	1016.5325	10	509.2768
	AMINTLAGNLSL	1216.6486	12	609.3359
	AMPLQVISSA	1031.5321	10	1032.5441
	AMVLPYYNVNA	1253.6115	11	1254.6216
	AMVLPYYNVNAHSIL	1761.876	15	881.9668

Accession Number	Peptide sequence	Mass	Length	*m/z*
	ANQLDFQPR	1088.525	9	545.272
	ARIQVVQQQGQNV	1466.7954	13	734.4092
	ARIQVVQQQGQNVF	1613.8638	14	807.9438
	AVLPPRGQQE	1093.588	10	547.8068
	AVLPPRGQQER	1249.6891	11	625.8555
	AVLPPRGQQERG	1306.7106	12	654.3655
	AVLPPRGQQERGE	1435.7532	13	718.8876
	AVLPPRGQQERGEQ	1563.8116	14	782.9183
	AVLPPRGQQERGEQQ	1691.8702	15	846.9482
	AVLPPRGQQERGEQQQ	1819.9288	16	910.977
	AVLPPRGQQERGEQQQD	1934.9558	17	968.4918
	AVPVGTGHF	883.4552	9	884.4666
	AVPVGTGHFI	996.5392	10	997.5544
	AVPVGTGHFIY	1159.6025	11	580.8131
	AVPVGTGHFIYNN	1387.6885	13	694.8564
	AVPVGTGHFIYNNG	1444.7098	14	723.3694
	AVPVGTGHFIYNNGD	1560.7208	15	781.3727
	AVPVGTGHFIYNNGDR	1716.8219	16	859.4235
	AVPVGTGHFIYNNGDRQ	1844.8805	17	923.4546
	AVPVGTGHFIYNNGDRQL	1957.9646	18	979.9982
	AYQVSNNQAR	1149.5526	10	575.7871
	DAQQLAEAF	1013.443	9	507.7322
	DAQQLAEAFN	1105.5039	10	1106.519
	DNIFSGF	798.3548	7	799.3723
	DNIFSGFD	913.3817	8	914.3954
	DNIFSGFDAQQL	1353.6201	12	677.8248
	DTSNDANQL	976.4098	9	489.2165
	DTSNDANQLDF	1238.5051	11	1239.5184
	DTSNDANQLDFQPR	1619.7175	14	810.871
	DTSNDANQLDFQPRR	1775.8186	15	888.9221

Accession Number	Peptide sequence	Mass	Length	*m/z*
	DTSNDANQLDFQPRRF	1922.8871	16	962.4615
	DVFAVPVGTGH	1097.5505	11	549.7855
	DVFAVPVGTGHF	1244.6189	12	623.3214
	EETICSLRL	1062.538	9	532.2799
	EGGGSEGRGQESSGD	1389.5392	15	695.783
	EGGGSEGRGQESSGDN	1503.5822	16	752.8126
	EGGGSEGRGQESSGDNIF	1763.7346	18	882.8801
	EGGGSEGRGQESSGDNIFSG	1907.7881	20	954.9073
	EGRGQESSGDNIF	1394.6062	13	698.3151
	EGRGQESSGDNIFSGF	1667.7175	16	834.8716
	ENAMINTLAGNL	1275.6129	12	638.819
	ENAMINTLAGNLSL	1475.729	14	738.8749
	ESSGDNIFSGF	1158.4829	11	580.251
	EVFDHNNEQ	1112.4523	9	1113.4596
	EVQQGQVL	899.4713	8	450.7473
	EYAMINTLAGNLSL	1508.7545	14	755.3824
	EYVAIKT	822.4487	7	823.4616
	EYVAIKTHE	1088.5502	9	545.2881
	EYVAIKTHEN	1202.5931	10	602.3085
	EYVAIKTHENA	1273.6302	11	637.8271
	EYVAIKTHENAM	1404.6708	12	703.3478
	FAVPVGTG	746.3962	8	747.409
	FAVPVGTGH	883.4552	9	884.4689
	FAVPVGTGHF	1030.5236	10	1031.5403
	FAVPVGTGHFIY	1306.671	12	654.3458
	FDHNNEQF	1049.4203	8	525.7213
	FIYNNGDR	997.4617	8	499.7417
	FIYNNGDRQ	1126.5043	9	564.2634
	FIYNNGDRQL	1238.6044	10	620.3149
	FIYNNGDRQLIV	1451.7408	12	726.8842

**Accession Number**	**Peptide sequence**	**Mass**	**Length**	***m/z***
	FIYNNGDRQLIVV	1550.8092	13	776.4181
	FLRAMPLQVISSA	1431.7908	13	716.9043
	FQQQQQQQQG	1246.5691	10	624.2963
	FQQQQQQQQGS	1333.6011	11	667.8124
	FQQQQQQQQGSE	1462.6437	12	732.3341
	FQQQQQQQQGSEG	1519.6652	13	760.8453
	FQQQQQQQQGSEGQ	1647.7238	14	824.8732
	FQQQQQQQQGSEGQQ	1775.7822	15	888.9038
	FQQQQQQQQGSEGQQQ	1903.8408	16	952.9302
	FVQGRGLQ	903.4926	8	452.7579
	FVQGRGLQG	960.5141	9	481.2688
	FVQGRGLQGI	1073.5981	10	537.8103
	FVQGRGLQGIM	1220.6335	11	611.3308
	FVQGRGLQGIMI	1333.7177	12	667.871
	FYFVQGRGLQGIM	1514.7704	13	758.3969
	FYLAGNPQN	1022.4821	9	512.2514
	FYLAGNPQNE	1151.5247	10	1152.5403
	FYLAGNPQNEF	1298.5931	11	650.3088
	FYLAGNPQNEFQ	1426.6517	12	714.3373
	FYLAGNPQNEFQQ	1554.7102	13	778.381
	FYLAGNPQNEFQQQ	1682.7688	14	842.3978
	FYLAGNPQNEFQQQQ	1810.8274	15	906.4254
	GGSEGRGQESSGDNIF	1595.6812	16	798.8534
	GGSEGRGQESSGDNIFSGF	1886.803	19	944.4139
	GHRSSVTGYDL	1190.568	11	596.2947
	GLLLPSYVNAPQL	1383.7761	13	692.9012
	GNIVRVEGGL	1012.5665	10	507.2941
	GSEGRGQESSGDNIFSGF	1829.7816	18	915.9028
	GVTEVFDH	902.4134	8	452.2171
	GVTEVFDHNN	1130.4993	10	566.2609

Accession Number	Peptide sequence	Mass	Length	*m/z*
	GVTEVFDHNNE	1259.5419	11	630.7814
	GVTEVFDHNNEQ	1387.6003	12	694.8123
	GYEETICSLRLKQNIGD	1903.9639	17	952.993
	HNRQESTIAPPGSSR	1635.8077	15	546.2793
	HQGAMVLPYYNVNA	1591.7452	14	796.8839
	HQGRLHQGAMVL	1361.6986	12	681.8605
	HRSSVTGYDL	1133.5465	10	567.784
	HRSSVTGYDLPIL	1456.7673	13	729.3937
	IFSGFDAQQ	1011.4661	9	506.7437
	IFSGFDAQQL	1125.5342	10	563.777
	IGDPWRAD	928.4402	8	465.2303
	IGDPWRADV	1027.5087	9	514.7665
	IGDPWRADVY	1190.572	10	596.2979
	IKARDSGF	920.4716	8	461.2466
	IKARDSGFE	1021.5192	9	511.771
	IKARDSGFEY	1184.5825	10	593.3036
	IMITGCPETF	1126.5039	10	1127.515
	IMITGCPETFQSF	1488.6628	13	745.3441
	INTLAGNL	814.4548	8	408.2385
	INTLAGNLS	901.4869	9	902.501
	INTLAGNLSL	1014.5709	10	508.2972
	INTLAGNLSLL	1127.655	11	564.8386
	IQSEAGVTE	932.4451	9	933.4644
	IQSEAGVTEV	1031.5134	10	1032.5345
	IQSEAGVTEVF	1200.5638	11	601.2939
	IQSEAGVTEVFDH	1430.6677	13	716.3453
	IQSEAGVTEVFDHNN	1659.7377	15	830.8792
	IQSEAGVTEVFDHNNE	1787.7961	16	894.9113
	IQSEAGVTEVFDHNNEQ	1915.8547	17	958.9445
	IQVVQQQGQNV	1239.6571	11	620.84

Accession Number	Peptide sequence	Mass	Length	*m/z*
	IQVVQQQGQNVF	1386.7256	12	694.3765
	IQVVQQQGQNVFN	1500.7684	13	751.3963
	IQVVQQQGQNVFNE	1629.811	14	815.9197
	ITGCPETF	866.3844	8	867.3981
	IVRVEGGL	841.5021	8	421.7634
	IVRVEGGLQ	969.5607	9	485.7919
	IVRVEGGLQA	1040.5978	10	521.3095
	IVVSVLDTSNDANQL	1586.8152	15	794.4196
	IYNNGDRQL	1091.5359	9	546.7799
	IYNNGDRQLI	1204.62	10	603.3214
	IYNNGDRQLIV	1304.6725	11	653.3468
	IYNNGDRQLIVV	1403.7408	12	702.882
	KLQGQNDRRGNIVR	1652.9182	14	551.9848
	KQNIGDPW	956.4716	8	479.2458
	KQNIGDPWR	1112.5726	9	557.2992
	KQNIGDPWRA	1183.6097	10	592.8159
	KQNIGDPWRAD	1298.6367	11	433.8893
	KQNIGDPWRADV	1379.6946	12	690.8586
	KQNIGDPWRADVF	1544.7736	13	773.3997
	LAEAFNVDVQ	1105.5292	10	1106.5437
	LAGNPQNE	841.3929	8	842.4092
	LAGNPQNEF	988.4614	9	495.2422
	LDFQPRR	930.5035	7	466.263
	LDTSNDANQ	976.4098	9	489.2148
	LDTSNDANQL	1089.4938	10	545.7571
	LDTSNDANQLDF	1351.5891	12	676.8065
	LDTSNDANQLDFQPR	1733.7856	15	867.9016
	LIIPQNF	843.4854	7	422.7536
	LIKARDSGF	1005.5607	9	503.7909
	LIKARDSGFE	1134.6033	10	568.3119

Accession Number	Peptide sequence	Mass	Length	*m/z*
	LIKARDSGFEY	1297.6666	11	649.8446
	LINTLAGNLSL	1127.655	11	564.8393
	LIRKLQGQND	1183.6672	10	592.8446
	LIRKLQGQNDR	1339.7684	11	447.6006
	LLLPSYVNA	988.5593	9	495.2905
	LLLPSYVNAPQ	1213.6707	11	607.8467
	LLLPSYVNAPQL	1326.7546	12	664.3914
	LLLPSYVNAPQLM	1473.7902	13	737.9084
	LLLPSYVNAPQLMY	1636.8534	14	819.44
	LLPSYVNAPQL	1213.6707	11	607.8468
	LLRAMPLQVISSA	1413.8014	13	707.9117
	LMINTLAGNL	1058.5795	10	530.3006
	LPSYVNAPQ	987.5025	9	494.7619
	LPSYVNAPQLM	1231.6271	11	1232.6399
	LPSYVNAPQLMY	1410.6853	12	706.3546
	LPYYNVNAHSIL	1402.7245	12	702.3735
	LQGQNDRRGNIVR	1524.8232	13	763.424
	LRAMPLQVIS	1126.6532	10	564.3399
	LRAMPLQVISS	1213.6853	11	607.856
	LRAMPLQVISSA	1284.7224	12	643.3748
	LRAMPLQVISSAY	1463.7806	13	732.9028
	LRLKQNIGDPW	1338.7407	11	670.3824
	LRLKQNIGDPWR	1494.8419	12	499.291
	LRLKQNIGDPWRAD	1680.9059	14	561.312
	LSAHQGRLHQGA	1273.6639	12	637.8456
	LSAHQGRLHQGA	1274.6479	12	638.3358
	LSAHQGRLHQGAM	1404.7045	13	703.3636
	LSAHQGRLHQGAMV	1503.7728	14	752.8988
	LSAHQGRLHQGAMVL	1632.8518	15	817.439
	LYLAGNPQNEF	1264.6088	11	633.3174

Accession Number	Peptide sequence	Mass	Length	*m/z*
	LYLAGNPQNEFQ	1392.6674	12	697.3459
	LYLAGNPQNEFQQQQ	1776.843	15	889.4347
	MINTLAGN	832.4113	8	833.4265
	MINTLAGNL	945.4954	9	946.5159
	MINTLAGNLS	1014.5168	10	508.2687
	MINTLAGNLSL	1161.6063	11	581.8168
	MPLQVISSA	944.5001	9	945.5134
	MVLPYYN	898.4258	7	899.4396
	MVLPYYNV	997.4943	8	998.5076
	MVLPYYNVN	1111.5372	9	556.7802
	MVLPYYNVNA	1182.5743	10	592.3002
	MVLPYYNVNAHSIL	1632.8334	14	817.4301
	MYFVQGRGL	1069.5378	9	535.7791
	MYFVQGRGLQ	1197.5964	10	599.8083
	MYFVQGRGLQG	1254.6179	11	628.3231
	MYFVQGRGLQGIM	1530.7323	13	766.3807
	NAMINTLAGN	1017.4913	10	509.7565
	NAMINTLAGNL	1130.5754	11	566.2993
	NAMINTLAGNLS	1217.6074	12	609.8158
	NAMINTLAGNLSL	1330.6915	13	666.3569
	NAMINTLAGNLSLL	1459.7705	14	730.8984
	NAQRPSNRIQSEAGVTE	1855.9136	17	928.971
	NEEVQQGQV	1029.4727	9	1030.4875
	NEEVQQGQVL	1142.5568	10	572.2913
	NEEVQQGQVLIIPQNF	1876.9294	16	939.4764
	NIFSGFD	798.3548	7	799.3682
	NIFSGFDA	869.3919	8	870.4048
	NIFSGFDAQ	997.4505	9	998.4671
	NIFSGFDAQQ	1125.509	10	1126.5225
	NIFSGFDAQQL	1238.5931	11	1239.6064

Accession Number	Peptide sequence	Mass	Length	*m/z*
	NIFSGFDAQQLAE	1438.6729	13	720.3492
	NIVRVEGGL	955.545	9	478.7834
	NIVRVEGGLQ	1083.6036	10	542.8127
	NRIQSEAGVTE	1202.5891	11	602.3058
	NRQESTIAPPGSS	1342.6477	13	672.3363
	NRQESTIAPPGSSRS	1585.7808	15	793.9012
	NRQESTIAPPGSSRSE	1714.8234	16	858.4242
	NRQESTIAPPGSSRSEY	1877.8867	17	939.9572
	NTLAGNLSL	901.4869	9	902.4999
	NVDVQLIR	955.545	8	478.7834
	NVNAHSIL	866.461	8	867.4749
	PRGLLLPSY	1014.5862	9	508.3027
	PSNRIQSEAGVTEV	1485.7423	14	743.8863
	PSYVNAPQL	987.5025	9	988.522
	PSYVNAPQLM	1118.543	10	1119.5601
	PSYVNAPQLMY	1281.6063	11	641.8182
	PSYVNAPQLMYF	1444.6697	12	723.3464
	PVGTGHF	713.3497	7	714.3633
	PVGTGHFIYNNGDRQ	1673.791	15	837.91
	PVGTGHFIYNNGDRQL	1787.859	16	894.9412
	PYYNVNA	839.3813	7	840.3976
	PYYNVNAHSI	1176.5564	10	589.2888
	PYYNVNAHSIL	1289.6404	11	645.8344
	QAVLPPRGQQER	1360.7211	12	681.3721
	QAVLPPRGQQERGE	1546.7852	14	774.4047
	QAVLPPRGQQERGEQ	1674.8438	15	838.4341
	QEGGGSEGRGQES	1259.5013	13	630.7628
	QEGGGSEGRGQESSG	1403.5549	15	702.7905
	QEGGGSEGRGQESSGD	1518.5818	16	760.3035
	QEGGGSEGRGQESSGDN	1632.6248	17	817.3242

Accession Number	Peptide sequence	Mass	Length	*m/z*
	QEGGGSEGRGQESSGDNIF	1892.7772	19	947.4014
	QESQQGQEQQEQG	1502.6233	13	752.3294
	QESQQGQEQQEQGQQ	1758.7405	15	880.3866
	QESQQGQEQQEQGQQG	1815.762	16	908.8986
	QESSGDNIF	978.393	9	979.4058
	QESSGDNIFSGF	1269.5149	12	635.7712
	QESSGDNIFSGFDAQQ	1710.7122	16	856.3657
	QESSGDNIFSGFDAQQL	1824.7802	17	913.4102
	QESTIAPPGSS	1072.5037	11	1073.5217
	QESTIAPPGSSR	1229.5887	12	615.8052
	QESTIAPPGSSRS	1315.6367	13	658.8332
	QESTIAPPGSSRSE	1444.6793	14	723.3557
	QESTIAPPGSSRSEY	1607.7427	15	804.8889
	QGQQFRGDQHQK	1438.6702	12	720.3466
	QGQQGQQFRGDQHQK	1751.8087	15	876.9178
	QGQQGQQGQQFRGDQH	1808.7938	16	905.4147
	QQERGEQQQD	1227.5116	10	614.7698
	QQGQQFRGDQH	1310.5752	11	656.2982
	QQGQQFRGDQHQK	1566.7288	13	784.3765
	QQGQQFRGDQHQKIR	1835.9139	15	612.9822
	QQGQQGQQFRGDQH	1623.7139	14	812.8687
	QQGQQGQQFRGDQHQ	1751.7723	15	876.8983
	QQGQQGQQFRGDQHQK	1879.8673	16	940.9471
	QQQEGGGSEGRGQE	1428.5865	14	715.3049
	QQQEGGGSEGRGQESSGDN	1888.7419	19	945.3696
	QQQQQQQQGSEGQQ	1629.6979	14	815.8642
	QQQQQQQQGSEGQQQ	1756.7725	15	879.4035
	QQQQQQQQGSEGQQQQ	1885.8151	16	943.9244
	QQQQQQQQGSEGQQQQ	1885.8151	16	943.9244
	QVSNNQARQLKHNR	1674.8662	14	559.3

Accession Number	Peptide sequence	Mass	Length	*m/z*
	QVVQQQGQNVF	1256.615	11	1257.6292
	QVVQQQGQNVF	1273.6415	11	1274.6543
	QVVQQQGQNVFNE	1516.7271	13	759.3755
	RAMPLQVISS	1116.5961	10	559.3107
	RAMPLQVISSA	1171.6383	11	586.8329
	RAMPLQVISSAY	1350.6965	12	676.3602
	RFYLAGNPQ	1064.5403	9	533.2808
	RFYLAGNPQN	1178.5833	10	590.3024
	RFYLAGNPQNE	1307.6259	11	654.8275
	RFYLAGNPQNEF	1454.6942	12	728.358
	RFYLAGNPQNEFQ	1583.7368	13	792.8866
	RFYLAGNPQNEFQQQ	1839.854	15	920.9435
	RGQESSGDNIF	1208.5421	11	1209.5574
	RGQESSGDNIFSGF	1499.6641	14	750.8441
	RIQSEAGV	858.4559	8	430.2385
	RIQSEAGVTE	1088.5461	10	545.2859
	RIQSEAGVTEV	1187.6146	11	1188.6307
	RIQSEAGVTEVF	1334.683	12	668.3531
	RIQVVQQ	869.5083	7	435.7661
	RIQVVQQQ	997.5669	8	499.7942
	RIQVVQQQG	1054.5884	9	528.3048
	RIQVVQQQGQ	1182.6469	10	592.3344
	RIQVVQQQGQN	1297.6738	11	649.8432
	RIQVVQQQGQNV	1395.7583	12	698.8909
	RIQVVQQQGQNVF	1542.8267	13	772.4287
	RIQVVQQQGQNVFN	1656.8696	14	829.4468
	RIQVVQQQGQNVFNE	1785.9121	15	893.9722
	RIQVVQQQGQNVFNEE	1914.9547	16	958.49
	RIQVVQQQGQNVFY	1705.89	14	853.958
	RLKQNIGDPWRAD	1567.8219	13	523.6182

Accession Number	Peptide sequence	Mass	Length	*m/z*
	RLKQNIGDPWRADV	1666.8903	14	556.6412
	RQESTIAPPGSSR	1384.7058	13	693.3655
	RQESTIAPPGSSRS	1471.7379	14	736.8813
	RQESTIAPPGSSRSE	1600.7805	15	801.4021
	RQESTIAPPGSSRSEY	1763.8438	16	882.9366
	SAHQGRLHQGAMV	1390.6888	13	696.3563
	SAHQGRLHQGAMVL	1519.7678	14	760.896
	SAYQVSNN	881.3879	8	882.4016
	SAYQVSNNQARQL	1477.7273	13	739.8759
	SEAGVTEV	790.3708	8	791.3845
	SEAGVTEVFDHNNEQ	1674.7122	15	838.3695
	SEGQQQQQEGGGSEGRG	1718.7091	17	860.3733
	SEGQQQQQEGGGSEGRGQ	1845.7837	18	923.9127
	SEGQQQQQEGGGSEGRGQE	1974.8263	19	988.4324
	SEGRGQESSGDNI	1334.5698	13	668.2959
	SEGRGQESSGDNIF	1481.6382	14	741.8313
	SEGRGQESSGDNIFSG	1625.6917	16	813.858
	SEGRGQESSGDNIFSGF	1772.7601	17	887.3926
	SGDNIFSGFDAQQL	1497.6736	14	749.8507
	SGFDAQQL	864.3977	8	865.4114
	SGFDAQQLA	935.4348	9	936.448
	SLLRAMPLQVISSA	1500.8334	14	751.4294
	SSGDNIFSGF	1030.4243	10	1031.4281
	SSVTGYD	727.3024	7	728.3135
	SSVTGYDL	840.3865	8	841.3987
	STIAPPGSS	815.4025	9	816.4166
	STIAPPGSSRS	1058.5356	11	530.2787
	STIAPPGSSRSE	1187.5782	12	594.8013
	STIAPPGSSRSEY	1350.6415	13	676.3328
	SVLDTSND	849.3716	8	850.3867

Accession Number	Peptide sequence	Mass	Length	*m/z*
	SVLDTSNDAN	1034.4517	10	1035.4771
	SVLDTSNDANQ	1163.4943	11	1164.5078
	SVLDTSNDANQL	1275.5942	12	638.8121
	SVLDTSNDANQLD	1390.6212	13	696.3234
	SVLDTSNDANQLDF	1537.6896	14	769.8567
	SVLDTSNDANQLDFQPR	1919.8861	17	960.9601
	SVTGYDLPIL	1076.5753	10	539.299
	SYVNAPQL	890.4498	8	891.4619
	SYVNAPQLMY	1200.5485	10	1201.5735
	TEVFDHNNE	1103.4519	9	552.7366
	TIAPPGSSRS	971.5036	10	972.5159
	TIAPPGSSRSE	1100.5461	11	1101.5598
	TIAPPGSSRSEY	1263.6095	12	632.8164
	TIEPRGLLL	1010.6124	9	506.3152
	TIEPRGLLLPSY	1357.7605	12	679.8959
	TIEPRGLLLPSYVNA	1641.9089	15	821.9662
	TIEPRGLLLPSYVNAPQL	1980.1044	18	991.0656
	TSNDANQLDFQPR	1504.6906	13	753.3527
	VAIKTHENAM	1128.5597	10	565.2897
	VEEGDVFAVPVGTGHF	1658.7939	16	830.4083
	VFAVPVGTGH	982.5236	10	492.2726
	VFAVPVGTGHF	1129.592	11	1130.6073
	VFAVPVGTGHFIY	1405.7394	13	703.882
	VFAVPVGTH	845.4647	9	846.476
	VFDHNNEQ	1001.4203	8	501.7231
	VLDTSNDANQ	1075.4781	10	538.7494
	VLDTSNDANQLDF	1450.6576	13	726.3393
	VLDTSNDANQLDFQPR	1831.87	16	916.9501
	VLIIPQNF	942.5538	8	943.5683
	VLPYYNV	866.4538	7	867.4714

Accession Number	Peptide sequence	Mass	Length	*m/z*
	VLPYYNVNA	1051.5338	9	526.779
	VNAPQLM	771.3949	7	772.4106
	VNAPQLMY	934.4583	8	468.2403
	VPVGTGHF	812.4181	8	813.4321
	VPVGTGHFIY	1088.5654	10	545.2935
	VPVGTGHFIYNNGD	1489.6837	14	745.8518
	VPVGTGHFIYNNGDRQL	1885.9435	17	629.6577
	VQGRGLQGIM	1073.5652	10	537.7957
	VQGRGLQGIMI	1186.6492	11	594.3358
	VQQQGQNVF	1046.5145	9	524.2671
	VQQQGQNVFNE	1289.6	11	645.8112
	VRVEGGLQ	856.4766	8	857.4908
	VRYTIEPR	1032.5715	8	517.2961
	VRYTIEPRGL	1202.6771	10	602.3519
	VRYTIEPRGLL	1315.7612	11	658.8962
	VRYTIEPRGLLL	1428.8452	12	715.4342
	VSVLDTSNDANQL	1374.6627	13	688.344
	VTEVFDHNN	1073.4778	9	537.7493
	VTEVFDHNNE	1202.5204	10	602.2723
	VTEVFDHNNEQ	1330.579	11	666.3029
	VTEVFDHNNEQF	1477.6473	12	739.8337
	VVQQQGQNV	998.5145	9	500.2697
	VVQQQGQNVF	1145.5829	10	573.8042
	VVQQQGQNVFN	1259.6259	11	630.8247
	VVQQQGQNVFNE	1389.6525	12	695.8376
	VVQQQGQNVFNEE	1517.7111	13	759.8689
	VVRLSAHQGRLHQGA	1627.9019	15	814.9625
	VYTPRGGHRSSVTG	1472.7484	14	737.3853
	VYTPRGGHRSSVTGY	1635.8118	15	818.9189
	VYTPRGGHRSSVTGYD	1750.8386	16	584.6255

Accession Number	Peptide sequence	Mass	Length	*m/z*
	VYTPRGGHRSSVTGYDL	1845.9121	17	923.9677
	YDLPILR	888.5068	7	445.264
	YEETICSLR	1078.5294	9	540.2754
	YEETICSLRL	1225.6012	10	613.8121
	YEETICSLRLKQNIGD	1846.9425	16	924.4846
	YFVQGRGL	938.4974	8	470.2601
	YFVQGRGLQ	1066.5559	9	534.2892
	YFVQGRGLQG	1123.5774	10	562.8019
	YFVQGRGLQGI	1236.6615	11	619.3427
	YFVQGRGLQGIM	1367.7019	12	684.8658
	YFVQGRGLQGIMI	1496.7809	13	749.4041
	YLAGNPQ	761.3708	7	762.3851
	YLAGNPQN	875.4137	8	438.7178
	YLAGNPQNE	1004.4563	9	503.2408
	YLAGNPQNEF	1151.5247	10	576.776
	YLAGNPQNEFQ	1279.5833	11	640.8041
	YLAGNPQNEFQQ	1407.6418	12	704.8329
	YLAGNPQNEFQQQ	1535.7004	13	768.8635
	YLAGNPQNEFQQQQ	1663.759	14	832.8923
	YLAGNPQNEFQQQQQ	1792.8016	15	897.4148
	YLAGNPQNEFQQQQQ	1813.7996	15	907.9164
	YLAGNPQNEFQQQQQQ	1920.8602	16	961.4449
	YNVNAHSIL	1029.5243	9	515.7727
	YQVSNNQ	851.3773	7	426.6982
	YQVSNNQA	922.4144	8	462.218
	YQVSNNQAR	1079.4995	9	540.7601
	YQVSNNQARQ	1206.5741	10	604.2984
	YQVSNNQARQL	1319.6582	11	660.8409
	YQVSNNQARQLK	1447.7532	12	724.8879
	YQVSNNQARQLKHN	1698.855	14	850.4402

Accession Number	Peptide sequence	Mass	Length	*m/z*
	YQVSNNQARQLKHNR	1854.9561	15	928.4905
	YTIEPRG	834.4235	7	418.2242
	YTIEPRGL	947.5076	8	474.7661
	YTIEPRGLL	1060.5917	9	531.3085
	YTIEPRGLLL	1173.6758	10	587.85
	YTIEPRGLLLPS	1357.7605	12	679.8917
	YTIEPRGLLLPSY	1520.8239	13	761.4246
	YTPRGGHRSSVTGY	1536.7433	14	769.3839
	YTPRGGHRSSVTGYD	1651.7703	15	826.896
	YVAIKTHE	959.5076	8	480.7648
	YVAIKTHEN	1073.5505	9	1074.5645
	YVAIKTHENA	1144.5876	10	573.3066
	YVAIKTHENAM	1291.623	11	646.8224
	YVAIKTHENAMINTL	1733.8658	15	867.9475
	YVNAPQL	803.4177	7	804.4307
	YVNAPQLMY	1113.5165	9	1114.5391
F6HI56	AFSIPAREVDEVF	1478.7405	13	740.3817
	DEFQPFYGPAGENPQSF	1928.8217	17	965.4224
	DQHYTASL	933.4192	8	934.4337
	EESGMFPFPFGST	1447.5966	13	724.8115
	EESGMFPFPFGSTES	1663.6711	15	832.8485
	EESGMFPFPFGSTESK	1791.7661	16	896.8979
	EFQPFYGPAGENPQ	1561.6837	14	781.8585
	EFQPFYGPAGENPQSF	1813.7947	16	907.9092
	EQGRQQEQGQEQ	1425.6233	12	713.8262
	ESLAGDKNIVNA	1229.6251	12	615.824
	ESLAGDKNIVNAL	1342.7092	13	672.3652
	FPFPFGST	898.4225	8	899.4382
	FPFPFGSTES	1114.4971	10	1115.5095
	FQPFYGPAGENPQSF	1684.7521	15	843.39

Accession Number	Peptide sequence	Mass	Length	*m/z*
	FQQLQDMD	1039.4281	8	1040.4418
	FSIPAREVD	1032.5239	9	517.272
	FSIPAREVDE	1161.5665	10	581.7942
	FSIPAREVDEVF	1407.7034	12	704.8633
	FVLPAHL	795.4643	7	796.4761
	FVLPAHLDA	981.5283	9	982.5443
	FVLPAHLDAE	1110.5709	10	556.2963
	FVLPAHLDAEA	1181.608	11	591.8151
	FVVPAGHPL	935.5228	9	468.7726
	FVVPAGHPLIV	1147.6753	11	574.849
	FVVPAGHPLIVV	1246.7438	12	624.3827
	FVVPAGHPLIVVA	1317.7809	13	659.9016
	FVVPAGHPLIVVAGN	1488.8452	15	745.4355
	FVVPAGHPLIVVAGNN	1602.8882	16	802.4562
	FVVPAGHPLIVVAGNNRN	1873.0322	18	625.3557
	GVIVKASEQQIQALSQ	1697.9312	16	849.9802
	IAKLLQPVA	951.6117	9	476.8163
	IAKLLQPVAL	1064.6957	10	533.3586
	IRAGTTIY	893.4971	8	447.7591
	ITKGSMEGPFF	1228.5798	11	615.3031
	IVVAGNNRNLE	1197.6466	11	599.8335
	LAFSIPAR	873.5072	8	437.7655
	LAFSIPARE	1002.5498	9	502.286
	LAFSIPAREVDEVF	1591.8246	14	796.9254
	LAGDKNIVNAL	1126.6346	11	564.3279
	LEKEAKELA	1029.5706	9	515.7963
	LFVVPAGHPL	1048.6069	10	525.3124
	LFVVPAGHPLIV	1260.7594	12	631.3909
	LSKEPSISN	973.508	9	487.7641
	LSKEPSISNR	1129.6091	10	565.8145

Accession Number	Peptide sequence	Mass	Length	*m/z*
	LSKEPSISNRYG	1349.6938	12	675.8595
	NKGVIVKASEQQIQA	1611.8944	15	806.959
	PAGENPQSF	945.4192	9	946.4319
	PDEFQPFY	1041.4443	8	1042.4585
	PDEFQPFYGPAGENPQ	1791.7739	16	896.8965
	PDEFQPFYGPAGENPQS	1878.806	17	940.4154
	PYVFQDQHYTASL	1567.7307	13	784.8772
	QGRQQEQGQEQ	1297.5647	11	649.7951
	QGRQQEQGQEQE	1426.6073	12	714.3165
	QGRQQEQGQEQEQ	1554.6659	13	778.3462
	QQGQGQSTGEQR	1285.5647	12	643.7971
	QQNKGVIVKASE	1299.7146	12	650.8676
	QQQQSASPHYQR	1439.6542	12	720.838
	QQQSASPHYQR	1311.5956	11	656.8099
	QRLSSPLKRGM	1254.6866	11	419.2399
	QRLSSPLKRGML	1367.7708	12	456.9348
	REESGMFPFPFGSTE	1732.7402	15	867.3828
	RESLAGDKNIVNAL	1498.8103	14	750.4167
	SIPAREVDEVF	1260.635	11	631.3279
	SLAGDKNIVNA	1100.5825	11	551.303
	SNITKGSMEGP	1136.502	11	569.2623
	SNITKGSMEGPFF	1414.6438	13	708.3318
	TESKRVFNL	1092.5928	9	547.3062
	VASGRGTLSL	959.54	10	480.7824
	VFAKQNEW	1020.5029	8	511.2618
	VIVKASEQQIQA	1312.735	12	657.378
	VVKGEGYM	881.4316	8	882.4437
	VVKGEGYME	1026.4691	9	1027.4819
	VVPAGHPLIVVAGN	1341.7769	14	671.9
	VVVKGEGY	849.4596	8	425.7424

Accession Number	Peptide sequence	Mass	Length	*m/z*
	VVYVASGRGTLS	1207.656	12	604.8384
	VVYVASGRGTLSL	1320.7401	13	661.3821
	VYVASGRGTLSL	1221.6718	12	611.8467
	WFFPGPRG	962.4762	8	482.2483
	WFFPGPRGG	1019.4977	9	510.7593
	WFFPGPRGGH	1156.5566	10	579.2895
	WFFPGPRGGHA	1227.5938	11	614.8081
	YGPAGENPQSF	1165.5039	11	1166.5267
	YVASGRGTLSL	1122.6033	11	562.3121
A5C7L5	AANPISGE	757.3606	8	758.3726
	AANPISGETA	929.4454	10	930.4594
	AANPISGETAF	1076.5138	11	539.2683
	AANPISGETAFG	1133.5353	12	567.7769
	AANPISGETAFGE	1262.5779	13	1263.5916
	AANPISGETAFGEL	1375.6619	14	688.8424
	ALCLTSPLNHGVL	1336.7173	13	669.3702
	AQDVLSIPSTDGSSL	1510.7126	15	756.3669
	AQDVLSIPSTDGSSLGQL	1808.8768	18	905.4531
	AQKGFPPAVQ	1041.5607	10	521.7916
	AQKGFPPAVQG	1098.5822	11	550.3011
	AQKGFPPAVQGVVG	1353.7405	14	677.8815
	AQKGFPPAVQGVVGL	1466.8245	15	734.4233
	AQKGFPPAVQGVVGLG	1523.8459	16	762.938
	AQKGFPPAVQGVVGLGHTS	1848.9846	19	617.3397
	ARSKLGFSSSL	1151.6299	11	576.8259
	ATKMTATQIGPEVA	1432.7231	14	717.3671
	DATKMTATQIGPEVANID	1889.9041	18	945.9659
	DLARSKLGF	1005.5607	9	503.7914
	DLARSKLGFSSSL	1379.7408	13	690.8827
	DLARSKLGFSSSLL	1492.825	14	747.4254

Accession Number	Peptide sequence	Mass	Length	*m/z*
	DVLSIPSTDGSSLGQL	1609.7811	16	805.9044
	EAPYRLHPGID	1248.6251	11	625.3254
	FLGEAPYRLHPGID	1583.8096	14	792.9109
	FVDGGSNPKAPIIL	1426.782	14	714.4022
	FVGSAQTS	795.3763	8	796.3881
	FVGSAQTSA	866.4134	9	867.426
	GEAPYRLHPGID	1323.6571	12	662.8395
	GVVGLGHTS	825.4344	9	413.7275
	GVVGLGHTSIA	1009.5556	11	505.7886
	GVVGLGHTSIAL	1122.6396	12	562.3304
	GVVGLGHTSIALPTQL	1561.8828	16	781.9543
	IPSTDGSSLGQL	1173.5876	12	1174.6057
	IQKRTPLKPVPLVL	1601.0392	14	801.5316
	KRTPLKPVPLVL	1359.8965	12	680.9606
	LARSKLGFSSSL	1264.7139	12	633.3671
	LGHTSIALPTQL	1249.703	12	625.8628
	LGSTPLSISREGEY	1507.7518	14	754.8864
	LSIPSTDGSSLGQL	1373.7039	14	687.8639
	LTNDSVSSRPNAL	1373.6786	13	687.8484
	LTSPLNHGV	936.5029	9	469.2626
	LTSPLNHGVL	1049.5869	10	525.8043
	LTSPLNHGVLF	1196.6553	11	599.3392
	LVLHNRNNVW	1264.6676	10	633.3445
	LVSKNEATNL	1087.5873	10	544.8057
	PISGETAFG	877.4181	9	878.4307
	PISGETAFGE	1006.4607	10	1007.4721
	PISGETAFGEL	1119.5448	11	1120.562
	PKAPIILGS	894.5538	9	448.2876
	QLASHFGF	888.413	8	889.4288
	RVVPVNPAL	963.5865	9	482.8047

Accession Number	Peptide sequence	Mass	Length	*m/z*
	RVVPVNPALL	1076.6705	10	539.3464
	SIPSTDGSSLGQL	1260.6198	13	631.3204
	STPLSISREGEY	1337.6462	12	669.8344
	TATQIGPEVANID	1327.662	13	664.843
	VDGGSNPKAPII	1166.6295	12	584.3257
	VLSIPSTDGSSL	1174.6082	12	588.3148
	VLSIPSTDGSSLGQ	1359.6881	14	1360.6987
	VLSIPSTDGSSLGQL	1472.7722	15	737.4012
	VLSIPSTDGSSLGQLV	1571.8406	16	786.9315
	VQPIAPFGL	940.5381	9	941.5515
	VSHPLGSTP	893.4607	9	447.7406
	VSHPLGSTPL	1006.5447	10	504.2832
	VSHPLGSTPLSIS	1293.6929	13	647.8578
	VSHPLGSTPLSISR	1449.7939	14	484.2762
	VSHPLGSTPLSISRE	1578.8365	15	790.4316
	VSHPLGSTPLSISREG	1635.858	16	818.9402
	VSHPLGSTPLSISREGE	1764.9006	17	883.4636
	VSHPLGSTPLSISREGEY	1927.9639	18	964.9961
	VSKNEATNL	974.5032	9	488.2624
	VVGLGHTSIA	952.5342	10	477.2778
	VVGLGHTSIAL	1065.6182	11	533.8197
	VVGLGHTSIALPTQL	1504.8613	15	753.4426
A5AEI7	GQGQGQEQGQGQGQGQRE	1855.8157	18	928.9235
	IAQKLPER	953.5658	8	477.7937
	IAQKLPERCGSGQACQSM	1871.8982	18	936.9639
	IRQQAEQQQGGQ	1370.6538	12	686.3411
	IRQQAEQQQGGQG	1427.6753	13	714.8514
	IRQQAEQQQGGQGD	1541.7183	14	771.8772
	IVQRQQGQGQGQ	1327.6481	12	664.8386
	IVQRQQGQGQGQG	1383.6854	13	692.8578

Accession Number	Peptide sequence	Mass	Length	*m/z*
	IVQRQQGQGQGQGQ	1511.744	14	756.888
	IVQRQQGQGQGQGQG	1567.7815	15	784.907
	IVQRQQGQGQGQGQGQ	1697.8081	16	849.9178
	IVQRQQGQGQGQGQGQG	1752.8616	17	877.4483
	IVQRQQGQGQGQGQGQGQ	1880.9202	18	941.478
	IVQRQQGQGQGQGQGQGQG	1937.9415	19	969.9893
	LRQCCQALQNM	1272.6067	11	637.317
	MQIAQKLPE	1056.5637	9	529.2921
	PERCGSGQACQSMQ	1479.5779	14	740.7883
	QAEQQQGGQGD	1127.4479	11	564.7345
	QGQGQGQGQGQGQR	1395.6239	14	698.8276
	QGQGQGQGQGQGQRE	1524.6665	15	763.3467
	QGQGQGQGQGQGQREQQQ	1908.8423	18	955.436
	QGQGQGQGQGQRE	1339.5864	13	670.8073
	QGQGQGQGQGQREQ	1467.645	14	734.8378
	QGQGQGQGQGQREQQ	1595.7036	15	798.8655
	QGQGQGQGQGQREQQQ	1723.7622	16	862.8949
	QGQGQGQGQGQREQQQE	1852.8048	17	927.415
	QGQGQGQGQRE	1154.5065	11	578.2672
	QGQGQGQGQREQ	1282.5651	12	642.2977
	QGQGQGQGQREQQ	1410.6237	13	706.3267
	QGQGQGQGQREQQQE	1667.7247	15	834.875
	QGQGQGQGQREQQQEM	1798.7653	16	900.3932
	QGQGQGQREQQ	1225.5436	11	613.7855
	QGQGQGQREQQQ	1353.6022	12	677.8156
	QGQGQGQREQQQE	1482.6448	13	742.3343
	QGQGQGQREQQQEM	1613.6852	14	807.8534
	QGQGQGQWQGQGQGQRE	1855.8309	17	619.6187
	QGQGQREQQQE	1297.5647	11	649.793
	QGQGQREQQQEM	1428.6052	12	715.314

Accession Number	Peptide sequence	Mass	Length	*m/z*
	QIAQKLPER	1064.5978	9	533.3105
	QQAEQQQGGQGD	1255.5065	12	628.7638
	QQGQGQGQGQGQGQGQGQ	1754.7681	18	878.3989
	QQGQGQGQGQGQGQGQGQG	1811.7896	19	906.9125
	QQIQGQQF	975.4774	8	976.4912
	QQQQEQQW	1084.4574	8	543.2389
	REQQQEMMQIA	1422.6232	11	712.3215
	RQQIQGQQF	1131.5785	9	566.799
	RYIRQQAEQQQGGQGD	1860.8827	16	931.453
	YIRQQAEQQQGGQG	1589.7546	14	795.8903
	YIRQQAEQQQGGQGD	1705.7655	15	853.8951
	YIRQQAEQQQGGQGD	1704.7815	15	853.4028
	YIRQQAEQQQGGQGD	1705.7655	15	853.8972
D7U302	AGRVIVVPQNF	1198.6823	11	600.3523
	ALMMKAGDSGFEF	1434.616	13	718.3208
	ANQLDFQPR	1088.525	9	545.272
	ANQLDFQPR	1087.541	9	544.7811
	AVFPQRGQEEQGSEQQE	1946.8606	17	974.4443
	DISNDANQLDFQPR	1632.7379	14	817.3792
	DISNDANQLDFQPRR	1787.855	15	894.9401
	DISNDANQLDFQPRRF	1934.9235	16	968.4791
	FIQGRGLQGIM	1234.6492	11	618.3356
	FIYNNGNNRLIL	1450.7568	12	726.3901
	GNIVRVEGGL	1012.5665	10	507.2941
	HQFSGDQHQKIR	1479.733	12	740.8901
	HQFSGDQHQKIRE	1608.7756	13	805.4122
	IDISNDANQL	1101.5302	10	551.7761
	IDISNDANQLDFQPR	1745.822	15	873.9292
	IGFNAERL	919.4763	8	460.749
	IQGRGLQGIM	1071.5859	10	536.8036

Accession Number	Peptide sequence	Mass	Length	*m/z*
	IQSEAGVTE	932.4451	9	933.4644
	ISGCPETFQSF	1214.5277	11	1215.5531
	IVRVEGGL	841.5021	8	421.7634
	IVRVEGGLQ	969.5607	9	485.7919
	IVRVEGGLQA	1040.5978	10	521.3095
	IYNNGNNRL	1076.5363	9	539.2786
	LDFQPRR	930.5035	7	466.263
	LIRKLQGQND	1183.6672	10	592.8446
	LSAQEPSNRIQSEAGVTE	1914.9282	18	958.4815
	MEASIAPGRF	1093.5226	10	547.7716
	MINTLAGDLSL	1146.5955	11	574.3082
	MKAGDSGFEF	1087.4644	10	544.7432
	MPVKAIASA	914.4895	9	458.2549
	NIVRVEGGL	955.545	9	478.7834
	NIVRVEGGLQ	1083.6036	10	542.8127
	NRIQSEAGVTE	1202.5891	11	602.3058
	PQRGQEEQGSEQQED	1743.7295	15	872.8809
	PRGLLLPSY	1014.5862	9	508.3027
	RIQSEAGV	858.4559	8	430.2385
	RIQSEAGVTE	1088.5461	10	545.2859
	RVIVVPQNF	1070.6237	9	536.3224
	SAQEPSNRIQSEAG	1472.6855	14	737.3501
	SAQEPSNRIQSEAGVTE	1801.8442	17	901.9361
	VAIKTDEN	888.4553	8	445.2396
	VAIKTDENGMINT	1420.6868	13	711.3546
	VAIKTDENGMINTL	1517.7759	14	759.901
	VAIKTDENGMINTL	1533.7709	14	767.8972
	VAIKTDENGMINTLAGD	1776.8563	17	889.4377
	VIVVPQNF	914.5225	8	915.536
	VRVEGGLQ	856.4766	8	857.4908

Accession Number	Peptide sequence	Mass	Length	*m/z*
	VVSTGVGHF	901.4658	9	902.4788
	VVSTGVGHFIY	1177.6132	11	589.8174
	VYTPLGGRIGGITS	1389.7616	14	695.8931
	VYTPLGGRIGGITSF	1536.83	15	769.4271
	YFIQGRGLQGIM	1397.7125	12	699.8674
	YFIQGRGLQGIM	1381.7177	12	691.871
	YLAGSPQNEF	1124.5138	10	563.2695
F6GTY5	AVILPSADVSPPD	1279.6659	13	640.8424
	EEIMTRQEGGPIIY	1650.7922	14	826.4076
	FLGGGTHPTSIL	1198.6346	12	600.3292
	IASRTGPFEF	1123.5662	10	562.7936
	IMTRQEGGPIIY	1392.7072	12	697.3651
	ITMEPKSLF	1064.5576	9	533.2851
	IVEKPMHIGF	1169.6267	10	585.8245
	KKQPVEPTEPYNL	1541.809	13	771.916
	KQPVEPTEPYNL	1413.714	12	707.8689
	LGGGTHPTSIL	1051.5662	11	526.7939
	SALADSGVGIY	1051.5186	11	1052.5337
	TGFAPETLST	1022.492	10	1023.5067
	TRQEGGPIIY	1132.5876	10	567.3049
	YLVNTGEGQRL	1248.6462	11	625.3335
	YSALADSGVGIY	1214.5819	12	1215.5968
	YTIPAGSA	778.3861	8	779.3972
	YTIPAGSAF	925.4545	9	926.4684
F6H566	AASALPTKCGVQ	1144.5911	12	573.3073
	AASALPTKCGVQIG	1280.7089	14	641.3661
	DAASALPTKCGVQ	1225.6302	13	613.8257
	IGIPISMT	846.4521	8	847.4664
	IGIPISMTT	931.5049	9	932.5195
	IGIPISMTTN	1061.5427	10	1062.5579

Accession Number	Peptide sequence	Mass	Length	*m/z*
	IKEDAASALPTKCGVQ	1595.8518	16	798.9382
	KLLTPTTTD	988.5441	9	495.2828
	LKLLTPTTT	986.6012	9	494.3113
	LKLLTPTTTD	1101.6282	10	551.825
	PAAPCCNGVQNLKL	1425.6982	14	713.8478
	PYLTGGGNP	874.4185	9	875.4314
	PYLTGGGNPA	945.4556	10	946.4699
	PYLTGGGNPAAPCCN	1432.5989	15	717.2997
	PYLTGGGNPAAPCCNGVQNL	1943.8744	20	972.9366
	TGGGNPAAPCCNGVQNLKL	1811.8533	19	906.9411
	VQIGIPISM	972.5314	9	973.5462
F6HAU0	AVDDVDQRIYGSIV	1548.7783	14	775.3972
	DVLLEPGSVVPLDIG	1521.829	15	761.9268
	EDVLLEPGSVVPLDIGSA	1808.9407	18	905.4882
	LEPGSVVPLDIGSASQL	1702.8754	17	852.4513
	LLEPGSVVPLDIGSASQL	1793.9774	18	898.0023
	TILPNGQIIML	1228.6737	11	615.3485
	VDQRIYGSIV	1148.6189	10	575.3199
	VLLEPGSVVPLDIGSA	1564.8712	16	783.4486
	VLLEPGSVVPLDIGSASQL	1893.0459	19	947.5378
A5ASF5	AEIQPPHHI	1082.5509	9	542.2864
	AEIQPPHHIL	1195.6349	10	598.8307
	AVKAATAATAGGSL	1187.651	14	594.8364
	FGQQHTGQQGT	1187.532	11	594.7767
	QAVKAATAATAGGSL	1315.7096	15	658.866
	RAEQFGQQHTGQQG	1570.7236	14	524.5845
	RAEQFGQQHTGQQGT	1671.7714	15	836.8975
F6HMH7	IVTGKGPLENL	1139.655	11	570.8384
	LTGEFPGDYGW	1240.54	11	1241.5498
	LTGEFPGDYGWD	1355.5669	12	1356.5796

Accession Number	Peptide sequence	Mass	Length	*m/z*
	LTGEFPGDYGWDTAGL	1697.7573	16	849.8927
	PLYPGGSFD	951.4338	9	952.4484
	PLYPGGSFDPLG	1218.592	12	1219.6023
	PLYPGGSFDPLGLAD	1539.722	15	770.8744
	PLYPGGSFDPLGLADD	1632.7671	16	817.3973
	PPSYLTGEF	1009.4756	9	1010.4883
	PPSYLTGEFPG	1163.5498	11	1164.5657
	PPSYLTGEFPGD	1278.5768	12	1279.5927
	YATNFVPGK	995.5076	9	498.7651
	YRIAGGPLGEVTD	1346.683	13	674.3541
D7T5P6	IVPRAGGDDVTIGGIE	1567.8206	16	784.9226
	IVTIVPRAGGDDVTIGGIE	1881.0208	19	941.5204
	TIVPRAGGDDVTIGGIE	1668.8682	17	835.4463
	VTIVPRAGGDDVTIGGIE	1767.9366	18	884.9815
A5ASG6	AIYPGGAFDPLGL	1289.6655	13	645.8473
	AIYPGGAFDPLGLAD	1475.7296	15	738.8782
	AIYPGGAFDPLGLADD	1590.7565	16	796.3916
	IVTGKGPIENL	1139.655	11	570.8384
	LTGEFPGDYGW	1240.54	11	1241.5498
	LTGEFPGDYGWD	1355.5669	12	1356.5796
	LTGEFPGDYGWDTAGL	1697.7573	16	849.8927
	YATNFVPGK	995.5076	9	498.7651
A5BQN6	DVSIGGAPAGRIVM	1357.7024	14	679.8633
	DVSIGGAPAGRIVME	1486.745	15	744.3839
	DVSIGGAPAGRIVMEL	1599.829	16	800.9272
F6HYK6	AASKAEGKAIGID	1271.6721	13	636.8484
	FDLGGGTF	812.3704	8	813.3829
	IFDLGGGTF	925.4545	9	926.4672
	IFDLGGGTFD	1040.4814	10	1041.4954
D7SYI1	FALQEIGHL	1026.5498	9	514.2858

Accession Number	Peptide sequence	Mass	Length	*m/z*
	FLWGSQGYGL	1126.5447	10	564.2841
	LVEFPLNL	943.5378	8	944.5511
	RIVYGGGSERNPGGF	1564.7745	15	783.4012
	YVIPYVGL	922.5164	8	923.5309
A5AG74	AAEQIGTRGTQG	1187.5895	12	594.8055
	AHQDQPQKTQL	1334.6578	11	668.3408
	ERAAEQIGTRGTQG	1454.7225	14	728.3739
	RAAEQIGTRGTQG	1343.6906	13	672.857
F6GSG7	FGEKPVTVF	1022.5436	9	512.2826
	GIRNPEEIPWGETGAE	1753.827	16	877.9255
	GIRNPEEIPWGETGAEF	1900.8955	17	951.4609
	IRNPEEIPWGETGAE	1696.8057	15	849.4182
A5C9F1	AAAVPGGGMQLGSGQSWSL	1788.8464	19	895.4327
	NVNAGTTGGRVW	1230.6105	12	616.3165
	SLNVNAGTTGGRVW	1430.7266	14	716.3751
F6HC76	PDLPYDYGAL	1122.5233	10	1123.538
	PDLPYDYGALE	1251.5659	11	1252.5815
	PLVTKGPNLVPL	1246.7649	12	624.3937
	PLVTKGPNLVPLL	1359.8489	13	680.9363
	PLVTKGPNLVPLLG	1416.8704	14	709.447
	PLVTKGPNLVPLLGID	1644.9814	16	823.5022
	VSLPDLPYDYGALE	1550.7504	14	776.3871
F6H8X2	AALPDIHVQNAGL	1317.7041	13	659.8624
	AQKIFEGEGGTY	1298.6143	12	650.3182
	LLTGTQGILGGF	1175.655	12	588.839
F6H3T7	FTDGLLPNGNF	1194.5557	11	1195.5698
	IVIHNPGVEE	1105.5768	10	553.7993
	IVIHNPGVEED	1220.6036	11	611.313
	TEVIGPHAIPEW	1347.6823	12	674.8564
F6HNP2	ANVPNSARETSA	1215.5844	12	608.8027

Accession Number	Peptide sequence	Mass	Length	*m/z*
	ANVPNSARETSAF	1362.6527	13	682.3383
	IDWKETPEAHVF	1470.7142	12	491.2487
	PFEGFPFSTTL	1241.5968	11	621.8116
	WKETPEAHVF	1242.6033	10	622.3134
A5AKD8	DMSVGGVPAGRIVM	1403.6901	14	702.8582
	FTAGNGTGGESIYGSKF	1692.7631	17	847.3955
F6GUW1	FGNPSVSDPFPR	1318.6305	12	660.3265
	IDWKETPEAHVF	1470.7142	12	491.2487
	PLFGSGEASSL	1063.5186	11	1064.5325
	QIDWKETPEAHV	1434.6779	12	718.3507
	WKETPEAHVF	1242.6033	10	622.3134
F6GXE5	KLVPGNSAGTV	1121.5482	11	561.7904
	LKLVPGNSAGTVTAF	1473.8191	15	737.9216
	YLSSPGPTRDEID	1448.6783	13	725.35
D7TH57	LSYTPFLKSPPASAF	1624.85	15	813.4313
	PSIILGNSQHVD	1278.6567	12	640.3402
	PSIILGNSQHVDY	1441.7201	13	721.8721
F6GVX0	TVIAGTAERAPT	1185.6354	12	593.8283
F6GTT2	APYQRGGKIGLF	1333.7142	12	667.871
	LGRIPSAVGYQPTL	1470.8195	14	736.42
	PAPATTF	703.3541	7	704.3647
D7T227	AAVPSGASTGIY	1092.5452	12	1093.5593
	ARAAVPSGASTGIY	1319.6833	14	660.8522
	ARAAVPSGASTGIYEA	1519.7631	16	760.892
F6HI57	FKVEEGTL	921.4807	8	461.7512
	FVVPAGHPI	935.5228	9	468.7726
	IVRIPAGITL	1051.6753	10	526.848
	LEKEAKELA	1029.5706	9	515.7963
	LFVVPAGHPI	1048.6069	10	525.3124
F6H7H1	FGEIGIGTPPQTF	1362.6819	13	682.3527

Accession Number	Peptide sequence	Mass	Length	*m/z*
	IHYGTGAISGF	1121.5505	11	561.7859
	TVIFDTGSSNL	1152.5663	11	1153.58
D7TE89	AGGGVGGGAGGAGTGLGGGAGAGHG	1735.7986	25	868.9247
	AGGGVGSGAGGGVGGGGGFGGGGGGGV	1831.8197	27	916.9128
	GGAGGAGTGLGGGAGAGHG	1337.6072	19	669.8278
	GGGGGGGVGGGSGHGGGFGA	1414.5974	20	708.3175
	GIGVGAGAGAGQGSGTGSGS	1517.707	20	759.8679
	IGIGVGAGAGAG	898.4872	12	450.254
	IGIGVGAGAGAGQ	1026.5458	13	514.2812
D7SKE9	LVGGASLKPEF	1116.6179	11	559.32
	VGGASLKPEF	1003.5338	10	502.7772
D7T674	IEAYTPGSKVTYPIAAD	1794.9039	17	898.4652
	PGLTLGDTIPNL	1209.6605	12	605.8416
F6H3T8	VESPALAPPEVHID	1472.7511	14	737.387
D7TBW7	VESPALAPPEVHIDL	1585.8351	15	793.9301
F6GY46	AAAAVGAGGF	790.3973	10	791.407
	KPVDDAVPYSDTTYSD	1771.7788	16	886.9054
F6HG44	GVRNPEEIPWAETGAD	1739.8114	16	870.9185
	GVRNPEEIPWAETGADY	1902.8748	17	952.451
F6HZB0	GGGAGGGIGGGSGGGGGIGG	1314.5912	20	658.3191
	GGGVGGGGGIGGGAGGGIG	1269.6061	19	635.8143
	GGGVGGGGGIGGGAGGGIGGGF	1529.7334	22	765.882
	GLGGGGGFGGGAGGGGGVGGGAGGGF	1806.8033	26	603.2878
F6GW31	LAFKSAITHD	1101.5818	10	551.8035
	LTGTVPAGISRL	1183.6924	12	592.8572
	RFSGPIPSSISQL	1387.746	13	694.8857
F6H684	APVGTGFS	734.3599	8	735.3696
	PGFPGKLPF	958.5276	9	480.2735
D7U4U5	IINVHNSLPEPF	1378.7245	12	690.3735
	IINVHNSLPEPFL	1492.7925	13	747.4099

Accession Number	Peptide sequence	Mass	Length	*m/z*
D7T7C6	ASINVSGGHVNPAVTF	1568.7947	16	785.4141
	INVSGGHVNPAVTF	1410.7256	14	706.3751
	SINVSGGHVNPAVTF	1497.7576	15	749.8927
F6HEX2	IVTFPAGATEGHAEGKL	1696.8784	17	849.4408
	YTTDPFGF	946.4072	8	947.42
D7SR57	VSIGGLNPGTNKEL	1397.7515	14	699.8867
	VSIGGLNPGTNKELS	1484.7834	15	743.4026
D7TWQ4	PELENQIGAKF	1244.64	11	623.3323
F6HUT2	AFLPISAPYGEN	1277.6292	12	1278.6448
	AFLPISAPYGENF	1424.6975	13	713.3607
Q9M4H7	AAPPTPEPVA	948.4916	10	949.5059
	EPAPKPEPAPA	1084.5553	11	1085.5708
	VAAAPPTPEPVAEEPKE	1730.8726	17	866.4482
Q9FS43	VVPAPGGGSIYKNTS	1445.7515	15	723.887
	YVLAHPDAY	1047.5026	9	524.7622
D7T2N7|	VLVTNPNPIPIPLVD	1599.9236	15	800.976
	VTNPNPIPIPLVD	1387.7711	13	694.8971
F6I3V5	FDLGGGTF	812.3704	8	813.3829
	QAGAPGAGAGA	826.3933	11	827.3954
D7SNA2	TQIPLSGPNAVIGRA	1492.8362	15	747.4294
F6HC82	LIVPISNNGDRGFW	1588.7886	14	795.4069
	VGSEVPYPVIF	1205.6332	11	1206.6478
D7T8C3	VSGVLGLIPETGKF	1415.8024	14	708.9144
F6HKR3	FTPGPSIIL	943.5378	9	944.5523
	FTPGPSIILGNHQSVDY	1843.9104	17	922.9678
F6HPN2	VVEEPINPSVNVNY	1571.7831	14	786.9041
A5C4C2	DVYKIGGIGTVPVGR	1529.8566	15	510.9633
F6HNI6	YKIGGIGTVPVG	1159.66	12	580.8417
D7TWV8	NTISSDLGGAPFGNVVSF	1780.8632	18	891.4484
F6HMA2	PDDIDHAVL	993.4767	9	497.7486

	Accession Number	Peptide sequence	Mass	Length	*m/z*
		WVISNGGID	960.4553	9	961.4697
	F6HGK8	PSSGALSGLRPGGVL	1366.7568	15	684.3912
	D7T757	IDNVEKQTGSL	1202.6143	11	602.3171
	F6HSN5	AILGVGSPAPIL	1106.6699	12	554.3439
		PAPTSFVPGAAY	1176.5814	12	1177.5969
	F6HBC6	RVSVPPQTGDLPLQTL	1719.9519	16	860.99
	F6HN88	VQKALQPEPINYPF	1642.8718	14	822.4484
	D7TVC2	PLVPEIAHM	1005.5317	9	503.7763
	D7SQ37	SVVRNGGLAPGGFNF	1491.7469	15	746.8882
**FZ**	F6HZK2	AALIKARDSGFEY	1439.7408	13	720.8846
		AIKTDENAMINT	1335.634	12	668.8291
		ALIKARDSGFEY	1368.7037	12	685.3658
		AMINTLAGNLSLM	1379.6788	13	690.8561
		AMPVQVIASAY	1164.5848	11	583.3063
		AMPVQVIASAYQA	1363.6805	13	682.8538
		AMPVQVIASAYQAS	1450.7126	14	726.3707
		AMPVQVIASAYQASN	1564.7555	15	783.391
		AMPVQVIASAYQASNNE	1807.8411	17	904.9357
		ANQLDFQPR	1088.525	9	545.2744
		ARDSGFEYVAIK	1354.6881	12	452.5744
		AVPTGFGHYIYNNGNRQ	1907.8915	17	954.962
		DAQQLAEAFNVD	1319.5994	12	660.8145
		DAQQLAEAFNVDVQ	1568.7083	14	785.3651
		DAQQLAEAFNVDVQ	1546.7263	14	774.3784
		DENAMINTLAGN	1278.5398	12	640.2827
		DENAMINTLAGNLSLM	1737.7914	16	869.9133
		DENAMINTLAGNLSLMR	1893.8925	17	947.9633
		DQHQKIREVEEGD	1581.7383	13	791.884
		DQHQKIREVEEGDA	1652.7754	14	827.4033

Accession Number	Peptide sequence	Mass	Length	*m/z*
	DSGFEYVAIK	1127.5498	10	564.7879
	DVSNEANQLD	1103.473	10	552.7494
	DVSNEANQLDFQP	1475.6528	13	738.8418
	DVSNEANQLDFQPR	1632.7379	14	817.384
	EAGVTEVFDHNNEQ	1587.6801	14	794.8559
	EAGVTEVFDHNNEQF	1734.7485	15	868.39
	EEVQQGQVL	1010.5032	9	506.2632
	EEVQQGQVLVIPQNF	1726.8889	15	864.4604
	EGGGSEGRGQESSGDN	1503.5822	16	752.806
	EGGGSEGRGQESSGDNI	1616.6663	17	809.3381
	EGGGSEGRGQESSGDNIF	1781.7452	18	891.8889
	EGGGSEGRGQESSGDNIFSG	1925.7987	20	963.9167
	EGQQGQQGQQGQQGQQ	1694.7357	16	848.3822
	EGRGQESSGDNIF	1376.5956	13	689.3114
	EGRGQESSGDNIFSG	1520.6492	15	761.34
	EGRGQESSGDNIFSGF	1685.728	16	843.88
	ESQEGQQGREQEGQQ	1716.7299	15	859.3835
	ESQEGQQGREQEGQQG	1773.7513	16	887.8938
	ESSGDNIF	849.3505	8	850.367
	ESSGDNIFSG	993.4039	10	994.4218
	ESSGDNIFSGF	1140.4723	11	1141.4944
	ESSGDNIFSGFDAQQ	1582.6536	15	792.3413
	ESTIGAPGSSRSE	1258.579	13	630.3035
	EVEEGDA	729.2817	7	730.296
	EVEEGDAFAVPT	1244.556	12	623.2911
	EVEEGDAFAVPTGFGH	1660.7368	16	831.3851
	EVFDHNNEQ	1112.4523	9	1113.469
	EVFDHNNEQF	1259.5206	10	1260.5374
	EYVAIKTDENAMIN	1625.7606	14	813.8978
	FDAQQLAEAFNVD	1466.6677	13	734.3489

Accession Number	Peptide sequence	Mass	Length	*m/z*
	FTQEGGGSEGRGQE	1437.6121	14	719.81
	FYLAGNPQNE	1151.5247	10	576.7746
	FYLAGNPQNEFQQQ	1682.7688	14	842.3994
	GDQHQKIREVEEGDA	1709.7969	15	855.9128
	GGGSEGRGQESSGDNIF	1652.7026	17	827.3689
	GGGSEGRGQESSGDNIFSG	1796.7561	19	899.3927
	GGGSEGRGQESSGDNIFSGF	1942.8405	20	972.447
	GGSEGRGQESSGDNIFSG	1739.7346	18	870.881
	GNIVRVEGGLQ	1140.6251	11	571.3265
	GQESSGDNIFSGF	1343.563	13	672.796
	GQTVANEEVQQG	1258.579	12	630.3021
	GQTVANEEVQQGQ	1408.6195	13	705.3228
	GQTVANEEVQQGQVL	1620.772	15	811.3989
	GRGQESSGDNIFSG	1409.6171	14	705.823
	GRGQESSGDNIFSGF	1556.6855	15	779.3555
	GSEGQQQQQEGGGSEGRG	1774.7466	18	888.3926
	GSEGRGQESSGDNIFSG	1682.7131	17	842.3722
	GSEGRGQESSGDNIFSGF	1829.7816	18	915.907
	GVTEVFDHNNEQ	1388.5845	12	695.3056
	GVTEVFDHNNEQF	1534.6688	13	768.3486
	GYEETICSLRLKQ	1504.7886	13	753.4073
	HNRQESTIGAPGSSRSE	1811.8511	17	604.9613
	ISEAGVTEVFDHNNEQF	1932.8126	17	967.4215
	IVRVEGGLQ	969.5607	9	485.7919
	KQNIGDPWRAD	1299.6207	11	650.8245
	LDVSNEANQLD	1216.5571	11	609.292
	LDVSNEANQLDF	1363.6255	12	682.8252
	LDVSNEANQLDFQP	1588.7368	14	795.3851
	LDVSNEANQLDFQPR	1744.838	15	873.4348
	LGDQHQKIREVEEGDA	1822.8809	16	912.4574

Accession Number	Peptide sequence	Mass	Length	*m/z*
	LIKARDSGFEY	1297.6666	11	649.8469
	LPPRGQQERGE	1265.6476	11	633.8379
	LPPRGQQERGEQQQ	1671.8053	14	836.9159
	LPPRGQQERGEQQQD	1780.8452	15	891.4386
	LPSYVNAPQ	1009.4844	9	505.755
	MPVQVIA	772.4153	7	773.4305
	MPVQVIAS	859.4473	8	860.4634
	MPVQVIAS	860.4313	8	861.4462
	MPVQVIASAY	1093.5477	10	1094.5627
	NEANQLDFQPR	1330.6266	11	666.3272
	NEEVQQGQVLVIPQNF	1840.9319	16	921.4926
	NIVRVEGGLQ	1083.6036	10	542.8146
	NIVRVEGGLQA	1154.6407	11	578.3337
	NMINTLAGNLSLMR	1535.78	14	768.9032
	NPQNEFQQQ	1132.4785	9	1133.4971
	NRQESTIGAPGSSR	1459.7015	14	730.8658
	NRQESTIGAPGSSRSE	1674.7921	16	838.4106
	NVNANSVIYAIRGSA	1547.8055	15	774.9161
	PPRGQQERGEQQQD	1665.7455	14	833.8866
	PRGQQERGEQQQD	1568.7291	13	785.364
	QEGGGSEGRGQES	1259.5013	13	630.762
	QEGGGSEGRGQESSG	1403.5549	15	702.7897
	QEGGGSEGRGQESSGDN	1632.6248	17	817.3328
	QEGGGSEGRGQESSGDNI	1745.7089	18	873.8704
	QEGGGSEGRGQESSGDNIF	1892.7772	19	947.4058
	QEGQQGREQEGQ	1355.5702	12	678.798
	QESQEGQQGREQEGQQ	1827.762	16	914.8975
	QESQEGQQGREQEGQQG	1884.7834	17	943.4086
	QESSGDNI	831.3246	8	832.3425
	QESSGDNIF	978.393	9	979.4103

Accession Number	Peptide sequence	Mass	Length	*m/z*
	QESSGDNIFSG	1122.4465	11	562.2366
	QESSGDNIFSGF	1269.5149	12	635.7723
	QESSGDNIFSGFD	1384.5419	13	693.2858
	QESSGDNIFSGFDAQ	1583.6376	15	792.8346
	QESSGDNIFSGFDAQQ	1711.6962	16	856.8632
	QESSGDNIFSGFDAQQLA	1894.8333	18	948.4402
	QESSGDNIFSGFDAQQLE	1894.8333	18	948.4402
	QESTIGAPGSS	1015.4458	11	1016.4626
	QESTIGAPGSSR	1189.5575	12	595.7906
	QESTIGAPGSSRS	1258.579	13	630.3027
	QESTIGAPGSSRSE	1386.6375	14	694.3324
	QFLGDQHQKI	1195.5985	10	598.8127
	QGQTVANEEVQQGQ	1514.696	14	758.3632
	QGQTVANEEVQQGQVL	1888.9296	16	945.4689
	QGSEGQQQQEGGGSEGRG	1885.7787	19	943.9091
	QHQKIREVEEGD	1449.6848	12	725.8569
	QHQKIREVEEGDA	1520.7219	13	761.377
	QHQKIREVEEGDAF	1667.7903	14	834.9109
	QKIREVEEGD	1184.5673	10	593.2969
	QKIREVEEGDA	1255.6044	11	628.8158
	QKIREVEEGDAF	1402.6729	12	702.3506
	QKIREVEEGDAFAVPT	1770.8788	16	886.4557
	QNIGDPWRA	1038.4883	9	520.257
	QNIGDPWRAD	1153.5153	10	577.7706
	QQEGGGSEGRGE	1171.4854	12	586.7544
	QQEGGGSEGRGQES	1387.5599	14	694.7925
	QQEGGGSEGRGQESSG	1531.6135	16	766.821
	QQEGGGSEGRGQESSGDNI	1873.7675	19	937.9019
	QQERGEQQQD	1227.5116	10	614.7686
	QQFLGDQHQK	1210.5731	10	606.301

Accession Number	Peptide sequence	Mass	Length	*m/z*
	QQFLGDQHQKI	1323.6571	11	662.8419
	QQGQQGQQGQQF	1343.5854	12	672.8068
	QQGQQGQQGQQGQQF	1656.724	15	829.3758
	QQGQQGQQGQQGQQGQQ	1839.8208	17	920.9302
	QQGQTVANEEVQQGQ	1643.7386	15	822.8853
	QQGQTVANEEVQQGQVL	1837.8806	17	919.9579
	QQQEGGGSEGRGQESSGDN	1888.7419	19	945.3842
	QQQGQTVANEEVQQG	1642.7546	15	822.3932
	QQQGQTVANEEVQQGQ	1753.7867	16	877.9091
	QQQGQTVANEEVQQGQVL	1965.9392	18	983.9875
	QQQQEQQQGSEGQQQ	1757.7565	15	879.8968
	QQQQQEGGGSEGRG	1427.6025	14	714.8136
	QQQQQEGGGSEGRGQE	1684.7037	16	843.3644
	QSEAGVTEVFDHNNEQ	1785.7441	16	893.8864
	QTVANEEVQQGQ	1329.6161	12	665.8214
	QTVANEEVQQGQVL	1524.7419	14	763.3857
	QVQQQGQTVANEE	1428.6844	13	715.3555
	QVVQQQGQTVANEEVQQG	1951.9236	18	976.9796
	RDSGFEYVAIK	1283.651	11	428.8948
	REVEEGDAF	1050.4618	9	526.243
	RFYLAGNPQNE	1307.6259	11	654.8267
	RFYLAGNPQNEFQQ	1710.8114	14	856.4208
	RFYLAGNPQNEFQQQ	1838.87	15	920.4498
	RGQESSGDNIFSG	1352.5956	13	677.3118
	RGQESSGDNIFSGF	1556.6855	14	779.3555
	RGQQERGEQQQ	1648.8057	11	825.411
	RQESTIGAPGSSRSE	1560.7491	15	781.3876
	SAYQASNNEAKQL	1422.6738	13	712.3503
	SAYQASNNEAKQLK	1550.7688	14	776.4006
	SEAGVTEVFDHN	1303.568	12	652.7974

Accession Number	Peptide sequence	Mass	Length	*m/z*
	SEAGVTEVFDHNN	1417.611	13	709.8202
	SEAGVTEVFDHNNE	1546.6536	14	774.3419
	SEAGVTEVFDHNNEQ	1674.7122	15	838.3732
	SEAGVTEVFDHNNEQF	1821.7805	16	911.9075
	SEGQQQQQEGGGSEGR	1660.7037	16	831.3701
	SEGQQQQQEGGGSEGRG	1718.7091	17	860.3728
	SEGQQQQQEGGGSEGRGQ	1845.7837	18	923.9113
	SEGQQQQQEGGGSEGRGQE	1974.8263	19	988.4287
	SEGRGQESSGDNIF	1481.6382	14	741.8368
	SEGRGQESSGDNIFSG	1625.6917	16	813.8618
	SEGRGQESSGDNIFSGF	1754.7495	17	878.3905
	SEGRGQESSGDNIFSGFD	1887.7871	18	944.9093
	SGFDAQQLAEAF	1282.5829	12	642.3047
	SGFEYVAIK	1012.5229	9	507.2731
	SNEANQLDFQPR	1417.6586	12	709.8452
	SNRIQSEAGVTEVF	1535.7579	14	768.8923
	SVIYAIRGSA	1035.5713	10	518.7977
	SVLDVSNEANQL	1309.6125	12	655.8196
	SVLDVSNEANQLD	1402.6576	13	702.3451
	SVLDVSNEANQLDF	1549.726	14	775.8768
	SVLDVSNEANQLDFQP	1796.8192	16	899.4253
	SVLDVSNEANQLDFQPR	1930.9385	17	966.4862
	SVTGYDLPVLQK	1318.7133	12	660.3695
	TDENAMIN	922.3702	8	462.1976
	TDENAMINT	1023.4179	9	512.7217
	TDENAMINTLAGN	1379.5875	13	690.8063
	TDENAMINTLAGNL	1492.6715	14	747.3491
	TDENAMINTLAGNLSL	1691.8036	16	846.9167
	TEVFDHNNEQF	1378.579	11	690.3032
	TGYDLPVLQK	1132.6128	10	567.3196

Accession Number	Peptide sequence	Mass	Length	*m/z*
	TIGAPGSSRSE	1060.5149	11	531.2697
	TVANEEVQQGQVL	1413.71	13	707.8689
	VAIKTDENAMIN	1333.6548	12	667.8408
	VAIKTDENAMINT	1434.7024	13	718.3662
	VEEGDAFAVPT	1155.506	11	578.7657
	VLDVSNEANQLD	1315.6255	12	658.826
	VLDVSNEANQLDFQP	1709.7872	15	855.9108
	VLDVSNEANQLDFQPR	1843.9064	16	922.9691
	VPTGFGHY	876.413	8	439.2203
	VPTGFGHYIY	1152.5603	10	577.2933
	VPTGFGHYIYN	1266.6033	11	634.3146
	VPTGFGHYIYNNGNR	1709.7798	15	855.9048
	VPTGFGHYIYNNGNRQ	1837.8384	16	919.9352
	VPTGFGHYIYNNGNRQL	1950.9224	17	651.3212
	VQQQGQTVANEEVQQGQ	1870.8657	17	936.455
	VQVIASAYQASNNEAKQ	1819.9064	17	910.9699
	VSNEANQLDFQP	1360.6259	12	681.3275
	VSNEANQLDFQPR	1516.7269	13	759.3785
	VSVLDVSNEANQ	1273.615	12	637.821
	VSVLDVSNEANQL	1386.6991	13	694.3635
	VSVLDVSNEANQLD	1501.726	14	751.879
	VSVLDVSNEANQLDFQP	1895.8877	17	948.9627
	VTEVFDHNNEQ	1330.579	11	666.3029
	VVSVLDVSNEANQLDFQP	1972.9741	18	987.5046
	VVVSVLDVSNEANQL	1584.8359	15	793.4316
	VVVSVLDVSNEANQLD	1699.8628	16	850.9465
	YEETICSLRLKQ	1447.7671	12	724.8982
	YFVQGRGLQGIL	1349.7455	12	675.8858
	YLAGNPQNEFQQQ	1535.7004	13	768.866
	YLAGNPQNEFQQQQQQ	1919.8762	16	960.9563

Accession Number	Peptide sequence	Mass	Length	*m/z*
	YTIEPNGLLLP	1250.6522	11	626.3391
F6HZK3	AALIKARDSGFEY	1439.7408	13	720.8846
	ALIKARDSGFEY	1368.7037	12	685.3658
	AMINTLAGNLSLL	1345.7275	13	673.8776
	AMPLQVISSAY	1194.5955	11	598.3112
	AMPLQVISSAYQVS	1508.7545	14	755.3932
	AMPLQVISSAYQVSN	1622.7974	15	812.415
	AMPLQVISSAYQVSNNQA	1935.936	18	968.9901
	ANQLDFQPR	1088.525	9	545.2744
	APPGSSRSEY	1049.4778	10	525.7526
	ARDSGFEYVAIK	1354.6881	12	452.5744
	AVPVGTGH	736.3868	8	737.399
	AVPVGTGHFIY	1159.6025	11	580.8144
	AVPVGTGHFIYNNGD	1559.7368	15	780.8813
	AVPVGTGHFIYNNGDRQ	1844.8805	17	923.4567
	AVPVGTGHFIYNNGDRQL	1956.9806	18	979.5055
	DANQLDFQPR	1203.552	10	602.7885
	DAQQLAEAFNVD	1319.5994	12	660.8145
	DAQQLAEAFNVDVQ	1568.7083	14	785.3651
	DQHQKIREVEEGD	1581.7383	13	791.884
	DQHQKIREVEEGDVF	1827.8751	15	914.9546
	DSGFEYVAIK	1127.5498	10	564.7879
	DTSNDANQLDFQP	1485.5984	13	743.8136
	DTSNDANQLDFQPR	1619.7175	14	810.8741
	DVFAVPVGTGH	1097.5505	11	1098.5679
	DVFAVPVGTGHF	1244.6189	12	623.3229
	DVFAVPVGTGHFIY	1520.7664	14	761.3979
	EAGVTEVFDHNNEQ	1587.6801	14	794.8559
	EAGVTEVFDHNNEQF	1734.7485	15	868.39
	EEGDVFAVPVGTGH	1412.6572	14	707.3434

Accession Number	Peptide sequence	Mass	Length	*m/z*
	EEGDVFAVPVGTGHF	1559.7256	15	780.875
	EEVQQGQVL	1010.5032	9	506.2632
	EEVQQGQVL	1028.5138	9	515.2699
	EEVQQGQVLIIPQNF	1762.8866	15	882.4595
	EEVQQGQVLIIPQNFAA	1882.9789	17	942.5056
	EGDVFAVPVGTGH	1283.6146	13	642.8199
	EGDVFAVPVGTGHFIY	1706.8303	16	854.4329
	EGGGSEGRGQESSGDN	1503.5822	16	752.806
	EGGGSEGRGQESSGDNI	1616.6663	17	809.3381
	EGGGSEGRGQESSGDNIFSG	1925.7987	20	963.9167
	EGRGQESSGDNIF	1376.5956	13	689.3114
	EGRGQESSGDNIFSG	1520.6492	15	761.34
	EGRGQESSGDNIFSGF	1685.728	16	843.88
	EQGQQGQQGQQGQQ	1509.6556	14	755.8387
	EQGQQGQQGQQGQQGQQ	1822.7943	17	912.4105
	ESSGDNIF	849.3505	8	850.367
	ESSGDNIFSG	993.4039	10	994.4218
	ESSGDNIFSGF	1140.4723	11	1141.4944
	ESSGDNIFSGFDAQQ	1582.6536	15	792.3413
	ESTIAPPGSSRSE	1298.6102	13	650.319
	ESTIAPPGSSRSEY	1479.6841	14	740.8596
	EVEEGDVF	904.3814	8	905.3981
	EVEEGDVFAVPVGTGH	1640.7682	16	821.3998
	EVEEGDVFAVPVGTGHF	1787.8365	17	894.9343
	EVFDHNNEQ	1112.4523	9	1113.469
	EVFDHNNEQF	1259.5206	10	1260.5374
	FDAQQLAEAFNVD	1466.6677	13	734.3489
	FTQEGGGSEGRGQE	1437.6121	14	719.81
	FYLAGNPQNE	1151.5247	10	576.7746
	FYLAGNPQNEFQQQ	1682.7688	14	842.3994

Accession Number	Peptide sequence	Mass	Length	*m/z*
	GDVFAVPVGTGH	1210.6346	12	606.3193
	GGGSEGRGQESSGDNIF	1652.7026	17	827.3689
	GGGSEGRGQESSGDNIFSG	1796.7561	19	899.3927
	GGGSEGRGQESSGDNIFSGF	1942.8405	20	972.447
	GGSEGRGQESSGDNIFSG	1739.7346	18	870.881
	GNIVRVEGGLQ	1140.6251	11	571.3265
	GQESSGDNIFSGF	1343.563	13	672.796
	GQNVFNEEVQQGQVL	1687.8165	15	844.9243
	GRGQESSGDNIFSG	1409.6171	14	705.823
	GRGQESSGDNIFSGF	1556.6855	15	779.3555
	GSEGQQQQQEGGGSEGRG	1774.7466	18	888.3926
	GSEGRGQESSGDNIFSG	1682.7131	17	842.3722
	GSEGRGQESSGDNIFSGF	1886.8031	18	944.4082
	GVTEVFDHNNEQ	1388.5845	12	695.3056
	GVTEVFDHNNEQF	1534.6688	13	768.3486
	GYEETICSLRLKQ	1504.7886	13	753.4073
	HNRQESTIAPPGSSRSE	1851.8823	17	618.3065
	IQVVQQQGQNVFN	1500.7684	13	751.3985
	IVRVEGGLQ	969.5607	9	485.7919
	KQNIGDPWRAD	1299.6207	11	650.8245
	LDTSNDANQLD	1204.5208	11	603.2732
	LDTSNDANQLDFQP	1576.7004	14	789.3655
	LDTSNDANQLDFQPR	1732.8016	15	867.4177
	LIKARDSGFEY	1297.6666	11	649.8469
	LLLPSYVNAPQLM	1473.7902	13	737.9102
	LPPRGQQERGE	1265.6476	11	633.8379
	LPPRGQQERGEQQQ	1665.8182	14	833.9245
	LPPRGQQERGEQQQD	1780.8452	15	891.4386
	LPSYVNAPQ	1009.4844	9	505.755
	LPSYVNAPQLM	1247.6219	11	624.8235

Accession Number	Peptide sequence	Mass	Length	*m/z*
	MPLQVIS	802.4258	7	803.4417
	MPLQVISS	890.4419	8	891.4582
	MPLQVISSA	961.479	9	962.4952
	NDANQLDFQPR	1316.6108	11	659.3192
	NIVRVEGGLQ	1083.6036	10	542.8146
	NIVRVEGGLQA	1154.6407	11	578.3337
	NPQNEFQQQ	1132.4785	9	1133.4971
	NRQESTIAPPGSSRSE	1714.8234	16	858.4272
	NRQESTIAPPGSSRSEY	1877.8867	17	939.9596
	PPRGQQERGEQQQD	1665.7455	14	833.8866
	PRGQQERGEQQQD	1568.7291	13	785.364
	PVGTGHFIYNNGDRQ	1674.775	15	559.2706
	QEGGGSEGRGQES	1259.5013	13	630.762
	QEGGGSEGRGQESSG	1403.5549	15	702.7897
	QEGGGSEGRGQESSGDN	1632.6248	17	817.3328
	QEGGGSEGRGQESSGDNI	1745.7089	18	873.8704
	QEGGGSEGRGQESSGDNIF	1892.7772	19	947.4058
	QESSGDNI	831.3246	8	832.3425
	QESSGDNIF	978.393	9	979.4103
	QESSGDNIFSG	1122.4465	11	562.2366
	QESSGDNIFSGF	1269.5149	12	635.7723
	QESSGDNIFSGFD	1384.5419	13	693.2858
	QESSGDNIFSGFDAQ	1583.6376	15	792.8346
	QESSGDNIFSGFDAQQ	1711.6962	16	856.8632
	QESSGDNIFSGFDAQQLA	1894.8333	18	948.4402
	QESTIAPPGSS	1055.4771	11	1056.4949
	QESTIAPPGSSR	1211.5782	12	1212.5979
	QESTIAPPGSSRS	1315.6367	13	658.8323
	QESTIAPPGSSRSE	1427.6528	14	714.8443
	QESTIAPPGSSRSEY	1590.7162	15	796.3752

Accession Number	Peptide sequence	Mass	Length	*m/z*
	QGQNVFNEE	1046.4305	9	1047.447
	QGQNVFNEEVQQG	1458.6376	13	730.3323
	QGQNVFNEEVQQGQ	1586.696	14	794.3643
	QGQNVFNEEVQQGQVL	1798.8485	16	900.441
	QGSEGQQQQEGGGSEGRG	1885.7787	19	943.9091
	QHQKIREVEEGD	1449.6848	12	725.8569
	QHQKIREVEEGDV	1548.7532	13	775.39
	QHQKIREVEEGDVF	1695.8217	14	848.9266
	QKIREVEEGD	1184.5673	10	593.2969
	QKIREVEEGDVF	1430.7041	12	716.3679
	QNIGDPWRA	1038.4883	9	520.257
	QNIGDPWRAD	1153.5153	10	577.7706
	QNVFNEEVQQGQ	1418.6426	12	710.3395
	QNVFNEEVQQGQVL	1613.7686	14	807.8989
	QQEGGGSEGRGQ	1171.4854	12	586.7544
	QQEGGGSEGRGQES	1387.5599	14	694.7925
	QQEGGGSEGRGQESSG	1531.6135	16	766.821
	QQEGGGSEGRGQESSGDNI	1873.7675	19	937.9019
	QQERGEQQQD	1227.5116	10	614.7686
	QQGEQGQQGQQGQQ	1510.6396	14	756.3323
	QQGQNVFNEEVQQG	1586.696	14	794.3643
	QQGQNVFNEEVQQGQ	1714.7546	15	858.39
	QQGQNVFNEEVQQGQVL	1926.9071	17	964.4703
	QQGQQGEQGQQGQQ	1510.6396	14	756.3323
	QQGQQGQQGQQF	1343.5854	12	672.8068
	QQGQQGQQGQQGQQF	1656.724	15	829.3758
	QQGQQGQQGQQGQQGQQ	1839.8208	17	920.9302
	QQQEGGGSEGRGQESSGDN	1888.7419	19	945.3842
	QQQGQNVFNEE	1302.5476	11	652.2864
	QQQGQNVFNEEVQQG	1714.7546	15	858.39

Accession Number	Peptide sequence	Mass	Length	*m/z*
	QQQGQNVFNEEVQQGQ	1842.8132	16	922.4208
	QQQQQEGGGSEGRG	1427.6025	14	714.8136
	QQQQQEGGGSEGRGQE	1684.7037	16	843.3644
	QSEAGVTEVFDHNNEQ	1785.7441	16	893.8864
	QSEAGVTEVFDHNNEQF	1932.8126	17	967.4215
	QVISSAYQVSNNQAR	1646.8013	15	824.4056
	QVISSAYQVSNNQARQL	1887.9438	17	944.9863
	QVVQQQGQNVFNEEVQQGQVL	2381.1611	21	794.7357
	RAMPLQVISSAYQVS	1664.8556	15	833.4462
	RDSGFEYVAIK	1283.651	11	428.8948
	REVEEGDVF	1060.4825	9	531.2537
	REVEEGDVFAVPVGTGH	1796.8693	17	899.4506
	RFYLAGNPQNE	1307.6259	11	654.8267
	RFYLAGNPQNEFQQ	1710.8114	14	856.4208
	RFYLAGNPQNEFQQQ	1838.87	15	920.4498
	RGLQGIMIT	1003.5485	9	502.7872
	RGQESSGDNIFSG	1352.5956	13	677.3118
	RGQESSGDNIFSGF	1556.6855	14	779.3555
	RGQQERGEQQQ	1648.8057	11	825.411
	RIQVVQQQGQNVFN	1656.8696	14	829.4502
	RQESTIAPPGSSRSE	1601.7645	15	801.8978
	SEAGVTEVFDHN	1303.568	12	652.7974
	SEAGVTEVFDHNN	1417.611	13	709.8202
	SEAGVTEVFDHNNE	1546.6536	14	774.3419
	SEAGVTEVFDHNNEQ	1674.7122	15	838.3732
	SEAGVTEVFDHNNEQF	1821.7805	16	911.9075
	SEGQQQQQEGGGSEGR	1660.7037	16	831.3701
	SEGQQQQQEGGGSEGRG	1717.7251	17	859.8813
	SEGQQQQQEGGGSEGRGQ	1845.7837	18	923.9113
	SEGQQQQQEGGGSEGRGQE	1974.8263	19	988.4287

Accession Number	Peptide sequence	Mass	Length	*m/z*
	SEGRGQESSGDNIF	1480.6542	14	741.3408
	SEGRGQESSGDNIFSG	1625.6917	16	813.8618
	SEGRGQESSGDNIFSGF	1754.7495	17	878.3905
	SEGRGQESSGDNIFSGFD	1887.7871	18	944.9093
	SGFDAQQLAEAF	1282.5829	12	642.3047
	SGFEYVAIK	1012.5229	9	507.2731
	SLLRAMP	802.4371	7	402.2284
	SNDANQLDFQPR	1405.611	12	703.8202
	SNRIQSEAGVTEVF	1535.7579	14	768.8923
	STIAPPGSSRSE	1187.5782	12	594.8028
	STIAPPGSSRSEY	1350.6415	13	676.3353
	SVLDTSNDANQL	1275.5942	12	638.8098
	SVLDTSNDANQLD	1390.6212	13	696.326
	SVLDTSNDANQLDF	1537.6896	14	769.8589
	SVLDTSNDANQLDFQP	1762.8009	16	882.4191
	SVLDTSNDANQLDFQPR	1919.8861	17	960.9595
	SYVNAPQLMY	1200.5485	10	601.287
	TEVFDHNNEQF	1378.579	11	690.3032
	THENAMINTLAGNL	1513.7195	14	757.8765
	THENAMINTLAGNLSL	1713.8356	16	857.9352
	THENAMINTLAGNLSLL	1826.9197	17	914.477
	TIAPPGSSRSE	1100.5461	11	551.286
	TSNDANQLDFQPR	1506.6587	13	754.345
	VAIKTHENAMIN	1355.6868	12	678.8571
	VAIKTHENAMINT	1456.7344	13	729.3833
	VEEGDVFAVPVGTGH	1511.7256	15	756.88
	VEEGDVFAVPVGTGHF	1640.7834	16	821.4086
	VFAVPVGTGH	982.5236	10	983.5414
	VFAVPVGTGHFIY	1405.7394	13	703.8838
	VFNEEVQQGQVL	1410.6755	12	706.3503

Accession Number	Peptide sequence	Mass	Length	*m/z*
	VLDTSNDANQLD	1303.5891	12	652.8076
	VLDTSNDANQLDFQP	1697.7509	15	849.8893
	VLDTSNDANQLDFQPR	1832.854	16	917.441
	VPVGTGDFIYNNGDRQ	1751.8114	16	876.9199
	VPVGTGHF	794.4075	8	795.4229
	VPVGTGHFI	907.4916	9	454.7573
	VPVGTGHFIY	1070.5549	10	536.2896
	VPVGTGHFIY	1088.5654	10	545.2972
	VPVGTGHFIYN	1202.6084	11	602.317
	VPVGTGHFIYNN	1318.6194	12	660.3222
	VPVGTGHFIYNNG	1454.6232	13	728.337
	VPVGTGHFIYNNGD	1471.6732	14	736.851
	VPVGTGHFIYNNGDR	1645.7848	15	823.9194
	VPVGTGHFIYNNGDRQ	1774.8274	16	888.4304
	VPVGTGHFIYNNGDRQL	1887.9115	17	944.9717
	VSVLDTSNDANQ	1261.5786	12	631.8017
	VSVLDTSNDANQLDFQP	1883.8513	17	942.9388
	VTEVFDHNNEQ	1330.579	11	666.3029
	VVQQQGQNVFNEE	1517.7111	13	759.8727
	VVQQQGQNVFNEEVQQ	1872.8966	16	937.4642
	VVQQQGQNVFNEEVQQG	1929.9181	17	965.9735
	VVSVLDTSNDANQLDFQP	1960.9377	18	981.4861
	YEETICSLRLKQ	1447.7671	12	724.8982
	YLAGNPQNEFQQQ	1535.7004	13	768.866
	YLAGNPQNEFQQQQQQ	1919.8762	16	960.9563
	YNNGDRQLIVVS	1379.6569	12	690.8425
A5AEI7	GQGQGQGQGQGQGQGQRE	1784.7786	18	893.4083
	GQGQGQGQGQGQGQGQRE	1783.7946	18	892.9167
	GQGQGQGQGQGQGQGQREQ	1911.8531	19	956.9465
	GQGQGQGQGQGQGQRE	1600.6826	16	801.3596

Accession Number	Peptide sequence	Mass	Length	*m/z*
	GQGQGQGQGQGQREQQQE	1926.8528	18	964.4462
	GQGQGQGQGQREQQQE	1741.7728	16	871.9054
	GQGQGQGQREQQQEMM	1850.7635	16	926.4011
	GQGQGQGQREQQQEMMQ	1978.8221	17	990.433
	GQGQGQREQQQEMMQ	1793.7421	15	897.8899
	GQGQGQREQQQEMMQIA	1977.8633	17	989.9499
	GQGQREQQQEMMQIA	1792.7832	15	897.4077
	IRQQAEQQQGGQG	1426.6913	13	714.3617
	IRQQAEQQQGGQGD	1542.7023	14	772.365
	LPERCGSGQA	982.4832	10	492.2534
	LPERCGSGQACQS	1300.583	13	651.3047
	QIAQKLPE	908.4967	8	455.2598
	QAEQQQGGQGD	1127.4479	11	564.7358
	QFQQCERY	1083.4443	8	1084.4578
	QGQGQGQGQGQGQGQGQRE	1894.8267	19	948.4265
	QGQGQGQGQGQGQGQRE	1709.7466	17	855.887
	QGQGQGQGQGQGQGQREQ	1869.8313	18	935.9345
	QGQGQGQGQGQGQR	1395.6239	14	698.8257
	QGQGQGQGQGQGQREQQQ	1927.8368	18	964.937
	QGQGQGQGQGQR	1210.5439	12	606.2866
	QGQGQGQGQGQRE	1339.5864	13	670.8065
	QGQGQGQGQGQREQ	1467.645	14	734.8362
	QGQGQGQGQGQREQQ	1595.7036	15	798.8651
	QGQGQGQGQGQREQQQ	1723.7622	16	862.8954
	QGQGQGQGQGQREQQQE	1869.8313	17	624.2921
	QGQGQGQGQREQ	1282.5651	12	642.2966
	QGQGQGQGQREQQQ	1538.6821	14	770.3652
	QGQGQGQGQREQQQE	1667.7247	15	834.877
	QGQGQGQGREQ	1225.5436	11	613.7866
	QGQGQGQREQQQE	1482.6448	13	742.3356

Accession Number	Peptide sequence	Mass	Length	*m/z*
	QGQGQREQQQEMMQ	1736.7206	14	869.3783
	QGQWQGQGQGQGQGQRE	1855.8309	17	928.9264
	QQAEQQQGGQGD	1274.5011	12	638.2651
	QQQEQQW	956.3988	7	479.2112
	QQQQEQQW	1084.4574	8	543.2435
	QQQQEQQWL	1197.5415	9	599.7843
	QQQQEQQWLR	1353.6426	10	677.8358
	REQQQEMMQIA	1422.6232	11	712.3233
	YIRQQAEQQQGGQGD	1705.7655	15	853.8983
F6HI56	AREVDEVFAK	1162.5981	10	582.3121
	DKNIVNALEKEAKE	1600.8308	14	534.6229
	EESGMFPFPF	1202.4954	10	602.2614
	EESGMFPFPFG	1259.5168	11	630.7723
	EESGMFPFPFGSTE	1576.6392	14	789.3348
	EESGMFPFPFGSTESK	1791.7661	16	896.9001
	EKEAKELAF	1045.5443	9	523.7839
	EQEQEQGQE	1085.4261	9	543.7339
	EQGRQQEQGQEQE	1554.6659	13	778.3489
	FPGPRGGHA	894.446	9	448.235
	GDKNIVNALEKEAKE	1657.8522	15	829.943
	GEEGKGGGGQSGEKDE	1641.6155	16	821.8317
	GEGYMEMACPHVS	1441.5312	13	721.7805
	GPAGENPQSF	1002.4406	10	502.2328
	IPAREVDEVF	1173.6029	10	1174.6241
	IPAREVDEVFA	1244.64	11	623.3331
	IPAREVDEVFAK	1372.735	12	687.382
	KGGGGQSGEK	945.4628	10	473.7507
	NALEKEAKE	1031.5134	9	516.7682
	NLLSKEPSISN	1200.635	11	601.3318
	PAGENPQSF	946.4032	9	947.4195

Accession Number	Peptide sequence	Mass	Length	*m/z*
	QEQEQGQEQQEQNPY	1844.7449	15	923.3866
	QEQQEQNPY	1145.4625	9	1146.4867
	QGRQQEQGQEQEQE	1683.7085	14	842.8646
	QNKGVIVKA	938.5549	9	470.2893
	QPFYGPA	761.3384	7	762.3544
	QPVALPDEFQP	1222.5869	11	612.3064
	QPVALPDEFQPF	1369.6554	12	685.8429
	QQNKGVIVKA	1066.6135	10	534.3194
	QQQQGQGQSTGEQR	1541.6819	14	771.8574
	QQQQGQGQSTGEQRRE	1826.8256	16	914.4272
	QQQSASPHY	1027.4359	9	1028.4537
	QQSASPHY	899.3773	8	900.3931
	REESGMFPFPFGST	1603.6976	14	802.8654
	REESGMFPFPFGSTE	1732.7402	15	867.3846
	REVDEVFAK	1091.561	9	546.7924
	SITKGSMEGPFF	1343.6067	12	672.8167
	SNITKGSMEGP	1136.502	11	569.2635
	SNITKGSMEGPF	1283.5703	12	642.7993
	SNITKGSMEGPFF	1430.6388	13	716.3361
	SQREESGMFPFPFGSTE	1947.8308	17	974.9294
	SQREESGMFPFPFGSTESK	2163.9419	19	722.3282
	VDEVFAK	806.4174	7	404.2206
	VNALEKEAKE	1130.5819	10	566.303
	VNALEKEAKEL	1243.6659	11	622.8461
	YSNITKGSMEGPFF	1592.718	14	797.3741
F6I0M9	GGAPVAAEPAEGGGGEVH	1560.7168	18	781.3719
	GGAPVAAEPAEGGGGEVHH	1697.7756	19	849.9037
	ADLRDEHGN	1068.4471	9	535.2353
	ADLRDEHGNP	1164.5159	10	583.2766
	ADLRDEHGNPIQ	1405.6586	12	703.844

Accession Number	Peptide sequence	Mass	Length	*m/z*
	ADLRDEHGNPIQL	1518.7427	13	760.3786
	ADLRDEHGNPIQLT	1619.7903	14	810.9111
	ADLRDEHGNPIQLTD	1734.8173	15	868.428
	QAVHGGAPVAAEPAEGGGGEVH	1978.9132	22	990.4738
D7U302	ANQLDFQPR	1088.525	9	545.2744
	DANQLDFQPR	1203.552	10	602.7885
	DQHQKIREVQEGD	1581.7383	13	791.8848
	ERLAEAFNVD	1204.5724	10	603.2977
	FPQRGQEEQGSEQQE	1775.771	15	888.8986
	FPQRGQEEQGSEQQED	1890.798	16	946.4161
	GDQHQKIREVQEGDVF	1884.8966	16	943.4653
	GNIVRVEGGLQ	1140.6251	11	571.3265
	IDISNDANQLD	1216.5571	11	609.2914
	IREVQEGDVF	1191.5771	10	596.8016
	ISNDANQLDFQPR	1516.7269	13	759.3785
	IVRVEGGLQ	969.5607	9	485.7919
	LPQRGQEEQGSEQQ	1612.7441	14	807.3872
	NDANQLDFQPR	1316.6108	11	659.3192
	NIVRVEGGLQ	1083.6036	10	542.8146
	NIVRVEGGLQA	1154.6407	11	578.3337
	PLGGRIGGITSFDLP	1480.8037	15	741.4124
	QVGEQEEQQGGHQ	1435.5964	13	718.8119
	QVGEQEEQQGGHQF	1582.6648	14	792.3463
	REVQEGDVF	1078.493	9	540.2616
	SLIRAMP	802.4371	7	402.2284
	SNDANQLDFQPR	1405.611	12	703.8202
	SVIDISNDANQLDFQPR	1930.9385	17	966.4851
	VGEQEEQQGGHQ	1324.5643	12	663.2952
	VQEGDVF	793.3494	7	794.3654
A5C7L5	AANPISGETA	929.4454	10	930.4601

Accession Number	Peptide sequence	Mass	Length	*m/z*
	ANPISGETA	858.4083	9	859.4235
	ANPISGETAFGE	1191.5408	12	596.7837
	ANPISGETAFGEL	1326.6067	13	664.3169
	DGGSNPKAPIIL	1180.6451	12	591.3359
	DVLSIPSTDGSSLGQL	1587.7992	16	794.9147
	EAPYRLHPGIDVSHP	1668.8372	15	557.2905
	EAPYRLHPGIDVSHPL	1781.9213	16	594.9871
	ERVVPVNPA	961.5345	9	962.5498
	HPGIDVSH	860.4141	8	431.218
	IPSTDGSSLG	932.4451	10	933.4614
	IPSTDGSSLGQ	1060.5037	11	1061.521
	IPSTDGSSLGQL	1173.5876	12	1174.6063
	NPISGETA	787.3712	8	788.3868
	NPISGETAFGE	1120.5037	11	1121.5227
	NSMVQPRPGVW	1286.6078	11	644.3199
	PGIDVSHP	820.4079	8	411.2192
	PGIDVSHPL	933.4919	9	467.7578
	PISGETAFGEL	1219.5608	11	610.7992
	QDVLSIPSTDGSSLGQ	1585.7471	16	793.8894
	SIPSTDGSSLGQ	1147.5356	12	574.7793
	SIPSTDGSSLGQL	1260.6198	13	631.3235
	SPLNHGVLF	983.5076	9	492.7662
	TDGSSLGQL	858.4083	9	859.426
	VDGGSNPKAP	922.4508	10	923.4646
	VDGGSNPKAPII	1148.6189	12	575.3219
	VDGGSNPKAPIIL	1261.703	13	631.8648
	VPLVLDVNGRS	1168.6451	11	585.3351
Q9M4H7	APPTPEPVA	877.4545	9	878.4702
	APPTPEPVAEEPK	1360.6874	13	681.3569
	APPTPEPVAEEPKE	1489.73	14	745.8777

Accession Number	Peptide sequence	Mass	Length	*m/z*
	APPTPEPVAEEPKEAETA	1861.8944	18	931.9646
	EAPKEEGPAAEEEEKPAE	1920.8588	18	961.4494
	EPAPKPEPAPA	1084.5553	11	1085.5745
	EPAPKPEPAPADEAPK	1624.8096	16	813.4185
	EPAPKPEPAPADEAPKEE	1882.8948	18	942.4625
	QDETKEVVEQVEVETK	1871.9	16	936.9672
	TAEVVSATPALSE	1353.6064	13	677.804
	TPALSEEKTEES	1319.6093	12	660.8185
	TPEPVAEEPKEAETA	1596.7518	15	799.3884
F6H3T7	DGLLPNGNFELGPKPSD	1769.8472	17	885.9401
	DHSPLPGWMVE	1282.5652	11	642.2962
	DHSPLPGWMVES	1369.5973	12	685.8132
	EIVIHNPGVEED	1349.6462	12	675.8362
	IPPNIEDDHSPLPG	1499.7256	14	750.8751
	IVIHNPGVEED	1220.6036	11	611.3192
	LPNGNFELGPKPSD	1484.7147	14	743.371
F6GTY5	EGGGEERQESEGGEHE	1696.656	16	849.3439
	SELEEIMT	966.4216	8	967.4396
	SELEEIMTR	1122.5227	9	562.2738
	NPTATEIGIVLK	1254.7183	12	628.3728
	QPVEPTEPYN	1155.5084	10	578.7671
	QPVEPTEPYNL	1268.5924	11	635.311
F6H0K6	DKYSGVTYADLF	1391.6609	12	696.8435
	LPDAGPP	647.3278	7	648.3519
	PPEGIVIDDGPAEAAPEK	1803.8889	18	902.9586
F6HKF4	FNDVGPLDEEEIAR	1603.7366	14	802.8822
	ASGQEKEERAEP	1371.6266	12	686.8273
	ASGQEKEERAEPA	1442.6637	13	722.3459
D7T0Z5	AQQEEEEGGDKPDLVTG	1800.8013	17	901.4189
	QPKVEDAAGKGGPVFGAA	1698.8577	18	850.4298

	Accession Number	Peptide sequence	Mass	Length	*m/z*
**PA**	**F6HZK2**	AALIKARDSGFE	1276.6775	12	639.3539
		AALIKARDSGFEY	1439.7408	13	720.8853
		AEAFNVDVQLIR	1373.7302	12	687.8784
		AFAVPTGF	808.4119	8	809.4282
		AFAVPTGFG	865.4333	9	866.4502
		AFAVPTGFGH	1002.4923	10	1003.512
		AFAVPTGFGHY	1165.5557	11	1166.5769
		AFAVPTGFGHYIY	1441.703	13	721.8638
		AFAVPTGFGHYIYN	1555.746	14	778.8889
		AFAVPTGFGHYIYNNG	1727.7943	16	864.9142
		AFAVPTGFGHYIYNNGN	1842.8213	17	922.4286
		AFNVDVQLIR	1173.6506	10	587.8396
		AGNLSLMR	876.4487	8	439.2367
		AGNLSLMRAMPVQVIAS	1788.9226	17	895.4778
		AGNPQNEFQQQQQ	1515.6702	13	758.8499
		AGNPQNEFQQQQQQ	1643.7288	14	822.8798
		AMINTLAGNL	1032.5273	10	517.2767
		AMINTLAGNLSL	1232.6434	12	617.3337
		AMPVQVIASAY	1164.5848	11	583.3057
		AMPVQVIASAYQA	1363.6805	13	682.8546
		AMPVQVIASAYQAS	1450.7126	14	726.3707
		AMPVQVIASAYQASN	1565.7395	15	783.886
		AMPVQVIASAYQASN	1564.7555	15	783.3911
		AMPVQVIASAYQASNN	1678.7985	16	840.4138
		AMPVQVIASAYQASNNE	1807.8411	17	904.9345
		ANQLDFQPR	1089.509	9	545.7667
		ANQLDFQPRR	1243.6421	10	622.8342
		AQKPSNRIQSEAGVT	1584.8219	15	793.4263
		AQQLAEAF	898.416	8	450.2205

Accession Number	Peptide sequence	Mass	Length	*m/z*
	AQQLAEAFNVD	1204.5724	11	603.2983
	AQQLAEAFNVDVQ	1453.6813	13	727.8562
	AVPTGFGHY	947.4501	9	474.7377
	CAGVAVVR	798.4171	8	400.22
	DAFAVPTGFGH	1117.5192	11	559.772
	DAFAVPTGFGHY	1280.5825	12	641.304
	DAQQLAEAF	1013.443	9	507.7335
	DAQQLAEAFNVD	1319.5994	12	1320.6228
	DAQQLAEAFNVDVQ	1568.7083	14	785.3668
	DENAMINTLAGNLSLMR	1893.8925	17	947.9661
	DIFSGFDAQQLAEAF	1656.7783	15	829.4086
	DIFSGFDAQQLAEAF	1657.7623	15	829.8976
	DNIFSGFDAQ	1112.4774	10	557.2515
	DNIFSGFDAQQL	1353.6201	12	677.824
	DNIFSGFDAQQLAEAF	1771.8053	16	886.9207
	DQHQKIREVEEGD	1581.7383	13	791.8831
	DSGFEYVAIK	1127.5498	10	564.7879
	DVSNEANQL	988.4462	9	495.2352
	DVSNEANQLD	1103.473	10	552.7487
	DVSNEANQLDF	1250.5415	11	626.2845
	DVSNEANQLDFQPR	1631.7539	14	816.8948
	EANQLDFQPR	1216.5836	10	609.3055
	EGDAFAVPTGF	1091.4923	11	546.7587
	EGDAFAVPTGFGH	1285.5728	13	643.7996
	EGDAFAVPTGFGHY	1448.636	14	1449.6604
	EGGGSEGRGQESSG	1274.5123	14	638.2688
	EGGGSEGRGQESSGDNIFSG	1907.7881	20	954.9105
	EGQQGQQGQQGQQG	1438.6185	14	720.3218
	EGQQQQQEGGGSEGRG	1612.6825	16	807.3608
	EGQQQQQEGGGSEGRGQE	1869.7837	18	935.9081

Accession Number	Peptide sequence	Mass	Length	*m/z*
	EGRGQESSGDNIFSGF	1685.728	16	843.8774
	EGRGQESSGDNIFSGFD	1782.7445	17	892.3871
	EGRGQESSGDNIFSGFDAQ	1981.8402	19	991.9354
	ENAMINTLAGNLSLMR	1778.8655	16	593.9684
	ESSGDNIFSGF	1158.4829	11	580.2535
	ESSGDNIFSGFDAQ	1454.595	14	728.3118
	ESSGDNIFSGFDAQQL	1695.7375	16	848.884
	ESTIGAPGSSRSE	1276.5895	13	639.3082
	ESTIGAPGSSRSE	1258.579	13	630.2929
	EVEEGDAFAVPTGFGH	1660.7368	16	831.3841
	EVEEGDAFAVPTGFGHY	1823.8002	17	912.9165
	FAVPTGF	737.3748	7	738.3895
	FAVPTGFGHY	1094.5186	10	548.2722
	FAVPTGFGHYIY	1370.6659	12	686.3438
	FDAQQLAEAF	1138.5294	10	570.2766
	FNVDVQLIR	1102.6135	9	552.3188
	FNVDVQLIR	1103.5975	9	552.8108
	FYLAGNPQNEF	1298.5931	11	650.3096
	FYLAGNPQNEFQ	1426.6517	12	714.3409
	FYLAGNPQNEFQ	1427.6357	12	714.8315
	FYLAGNPQNEFQQQ	1704.7507	14	853.3939
	FYLAGNPQNEFQQQQ	1810.8274	15	906.4309
	FYLAGNPQNEFQQQQQ	1938.886	16	970.4608
	GFDAQQLAEAF	1195.5509	11	598.7892
	GGSEGRGQESSGDNIFSGF	1886.803	19	944.4203
	GNIVRVEGGLQ	1140.6251	11	571.3252
	GSEGQQQQQEGGGSEGRG	1894.7806	18	948.4084
	GSEGRGQESSGDNIFSGF	1829.7816	18	915.9064
	GSEGRGQESSGDNIFSGFD	1944.8085	19	973.4322
	GYEETICSLRLKQNIGD	1965.9465	17	983.9926

Accession Number	Peptide sequence	Mass	Length	*m/z*
	HNRQESTIGAPGS	1352.6433	13	677.3351
	HNRQESTIGAPGSS	1439.6753	14	720.8508
	HNRQESTIGAPGSSR	1611.7714	15	538.27
	HNRQESTIGAPGSSRSE	1812.8351	17	605.2909
	HRSSVTGYD	1020.4625	9	511.2438
	IFSGFDAQQL	1124.5502	10	563.2878
	IFSGFDAQQLAEAF	1564.7173	14	783.373
	IKARDSGFEY	1184.5825	10	593.303
	IKTDENAMINTLAGNL	1732.8666	16	867.447
	IKTDENAMINTLAGNLSL	1932.9827	18	967.5173
	IQSEAGVTE	932.4451	9	933.4593
	IVRVEGGL	841.5021	8	421.7637
	IVRVEGGLQ	969.5607	9	485.7927
	IYNNGNRQL	1091.536	9	546.7799
	IYNNGNRQLVVVS	1475.7732	13	738.9017
	LAGNLSLMR	989.5328	9	495.7781
	LAGNPQNEF	989.4454	9	990.4647
	LAGNPQNEFQQQQ	1500.6957	13	751.3615
	LAGNPQNEFQQQQQ	1628.7543	14	815.3921
	LAGNPQNEFQQQQQQ	1756.8129	15	879.4221
	LAGNPQNEFQQQQQQQ	1884.8715	16	943.4539
	LDVSNEANQLDFQPR	1745.822	15	873.9283
	LLLPSYVNAPQLL	1439.8387	13	720.9312
	MINTLAGNLSLMR	1464.7429	13	733.3866
	MRAMPVQVIASAY	1467.7214	13	734.8757
	NAMINTLAGNLSLMR	1649.8229	15	825.9281
	NEANQLDFQPR	1330.6266	11	666.3273
	NIFSGFDAQ	997.4505	9	499.7371
	NIFSGFDAQQL	1238.5931	11	620.3098
	NIFSGFDAQQLAEA	1509.71	14	755.871

Accession Number	Peptide sequence	Mass	Length	*m/z*
	NIVRVEGGLQ	1083.6036	10	542.814
	NMINTLAGNLSLMR	1535.78	14	768.9051
	NPQNEFQQQQQ	1387.6117	11	694.8184
	NPQNEFQQQQQQQ	1643.7288	13	822.8789
	NQLDFQPR	1016.5039	8	509.2642
	NRQESTIGAPGSSR	1458.7175	14	730.3734
	NRQESTIGAPGSSRSE	1675.7761	16	838.9023
	NVDVQLIR	956.5291	8	479.2769
	NVDVQLIRKLQG	1381.8041	12	691.9156
	NVDVQLIRKLQGQND	1739.9166	15	870.9753
	PNGLLLPSYVNAPQLL	1690.9293	16	846.4836
	PQNEFQQQQQQ	1402.6113	11	702.318
	PQNEFQQQQQQQ	1529.6859	12	765.857
	PQNEFQQQQQQQQ	1657.7444	13	829.8864
	PQNEFQQQQQQQQE	1786.787	14	894.4083
	PRGQQERGEQQQD	1568.7291	13	785.364
	PSYVNAPQL	987.5025	9	988.5201
	QEGGGSEGRGQE	1172.4694	12	587.2489
	QEGGGSEGRGQES	1259.5013	13	630.7654
	QEGGGSEGRGQESSG	1403.5549	15	702.7936
	QEGGGSEGRGQESSGD	1518.5818	16	760.3067
	QEGGGSEGRGQESSGDN	1632.6248	17	817.3274
	QEGGGSEGRGQESSGDNI	1745.7089	18	873.868
	QEGQQGQQGQQGQQG	1567.6611	15	784.8486
	QEGQQGREQEGQQG	1540.6501	14	771.3392
	QEGQQGREQEGQQGQQG	1853.7888	17	927.9104
	QEQQQGSEGQQQ	1356.5542	12	679.2908
	QESQEGQQGR	1128.4796	10	565.2531
	QESQEGQQGRE	1257.5221	11	629.775
	QESQEGQQGREQE	1514.6233	13	758.3252

Accession Number	Peptide sequence	Mass	Length	*m/z*
	QESQEGQQGREQEG	1571.6448	14	786.8373
	QESQEGQQGREQEGQQ	1827.762	16	914.8962
	QESQEGQQGREQEGQQG	1884.7834	17	943.4034
	QESSGDNI	831.3246	8	832.3392
	QESSGDNIFSGF	1268.5309	12	635.2784
	QESSGDNIFSGFD	1384.5419	13	693.2862
	QESSGDNIFSGFDAQ	1583.6376	15	792.8339
	QESSGDNIFSGFDAQQL	1824.7802	17	913.407
	QESTIGAPGSSRS	1258.579	13	630.3027
	QESTIGAPGSSRSE	1387.6215	14	694.827
	QESTIGAPGSSRSE	1404.6481	14	703.338
	QGREQEGQQGQQ	1354.5862	12	678.3072
	QGREQEGQQGQQGQQ	1667.7247	15	834.878
	QGREQEGQQGQQGQQG	1724.7462	16	863.3908
	QHQKIREVE	1148.5938	9	575.3095
	QHQKIREVEEG	1334.6578	11	668.3425
	QHQKIREVEEGD	1449.6848	12	725.8588
	QHQKIREVEEGDA	1520.7219	13	761.3741
	QHQKIREVEEGDAF	1667.7903	14	834.9125
	QKIREVEEGD	1184.5673	10	593.2968
	QKIREVEEGDAFAVPT	1770.8788	16	886.4557
	QLAEAFNVD	1005.4767	9	1006.4948
	QLDFQPR	885.4344	7	443.7302
	QQEGGGSEGRGQE	1300.528	13	651.2756
	QQEGGGSEGRGQES	1387.5599	14	694.7953
	QQEGGGSEGRGQESS	1474.592	15	738.3078
	QQEGGGSEGRGQESSG	1531.6135	16	766.8219
	QQEGGGSEGRGQESSGD	1646.6404	17	824.3361
	QQEGGGSEGRGQESSGDNI	1873.7675	19	937.8993
	QQERGEQQQD	1227.5116	10	614.7725

Accession Number	Peptide sequence	Mass	Length	*m/z*
	QQFLGDQHQKIR	1479.7582	12	740.892
	QQGQQFLGDQHQK	1523.7117	13	762.8695
	QQGQQFLGDQHQKIR	1792.8969	15	897.4647
	QQGQQGQQFLGD	1315.5792	12	658.806
	QQGQQGQQFLGDQHQK	1836.8503	16	919.4341
	QQGQQGQQGQQFLGD	1628.7179	15	815.3754
	QQGREQEGQQGQQ	1482.6448	13	742.3398
	QQGREQEGQQGQQGQQ	1795.7833	16	898.9075
	QQGREQEGQQGQQGQQG	1852.8048	17	927.4173
	QQQEGGGSEGRG	1171.4854	12	586.7562
	QQQEGGGSEGRGQE	1428.5865	14	715.3077
	QQQEGGGSEGRGQES	1515.6185	15	758.8244
	QQQEGGGSEGRGQESSG	1659.672	17	830.8531
	QQQEGGGSEGRGQESSGD	1774.699	18	888.3657
	QQQQEGGGSEGR	1242.5225	12	622.2749
	QQQQEGGGSEGRGQE	1556.6451	15	779.3378
	QQQQEGGGSEGRGQES	1643.6771	16	822.8529
	QQQQEGGGSEGRGQESSG	1787.7306	18	894.8806
	QQQQEGGGSEGRGQESSGD	1902.7576	19	952.3929
	QQQQQEGGGSEGR	1370.5811	13	686.3057
	QQQQQEGGGSEGRG	1427.6025	14	714.8164
	QQQQQEGGGSEGRGQ	1555.6611	15	778.8478
	QQQQQEGGGSEGRGQE	1684.7037	16	843.3669
	QQQQQEGGGSEGRGQES	1771.7357	17	886.884
	QQQQQEGGGSEGRGQESSG	1915.7892	19	958.9115
	QTVANEEVQQGQVL	1524.7419	14	763.3837
	REQEGQQGQQGQQGQQG	1870.8153	17	936.4338
	RFYLAGNPQN	1178.5833	10	590.3042
	RFYLAGNPQNE	1307.6259	11	654.8259
	RFYLAGNPQNEF	1455.6782	12	728.8551

Accession Number	Peptide sequence	Mass	Length	*m/z*
	RFYLAGNPQNEFQ	1582.7528	13	792.392
	RFYLAGNPQNEFQQ	1711.7954	14	856.9129
	RFYLAGNPQNEFQQQ	1839.854	15	920.9435
	RFYLAGNPQNEFQQQQ	1966.9285	16	984.4823
	RGQESSGDNIF	1208.5421	11	605.2826
	RGQESSGDNIFSG	1352.5956	13	677.3109
	RGQESSGDNIFSGF	1499.6641	14	750.8472
	RGQESSGDNIFSGFD	1614.6909	15	808.3605
	RIQSEAGVT	959.5036	9	480.7648
	RIQSEAGVTEV	1187.6146	11	594.8206
	RIQSEAGVTEVF	1334.683	12	668.3547
	RIQSEAGVTEVFD	1449.71	13	725.8697
	RQESTIGAPGSSR	1344.6746	13	673.3519
	RQESTIGAPGSSRSE	1588.7441	15	795.386
	SAYQASNNEAKQL	1422.6738	13	712.3494
	SAYQASNNEAKQL	1423.658	13	712.8445
	SEGQQQQQEGGGSEGR	1660.7037	16	831.3703
	SEGQQQQQEGGGSEGRG	1717.7251	17	859.8801
	SEGQQQQQEGGGSEGRG	1718.7091	17	860.3715
	SEGQQQQQEGGGSEGRGQ	1846.7677	18	924.4038
	SEGQQQQQEGGGSEGRGQE	1974.8263	19	988.4315
	SEGRGQESSGDNI	1334.5698	13	668.2983
	SEGRGQESSGDNIFSG	1625.6917	16	813.8605
	SEGRGQESSGDNIFSGF	1772.7601	17	887.3965
	SEGRGQESSGDNIFSGFD	1887.7871	18	944.9106
	SGDNIFSGFD	1057.4352	10	1058.4517
	SGDNIFSGFDAQ	1256.5309	12	1257.5485
	SGDNIFSGFDAQQLAEAF	1915.8588	18	958.9465
	SGFDAQQLAEAF	1283.5669	12	642.798
	SGFEYVAIK	1012.5229	9	507.2737

Accession Number	Peptide sequence	Mass	Length	*m/z*
	SGFEYVAIKTD	1228.5975	11	615.3125
	SGFEYVAIKTDEN	1471.6831	13	736.8566
	SQEGQQGREQEGQQG	1645.6927	15	823.8623
	SQEGQQGREQEGQQGQ	1772.7673	16	887.4033
	SQEGQQGREQEGQQGQQ	1900.8259	17	951.4338
	SSGDNIFSGF	1051.4222	10	526.7214
	SSGDNIFSGFD	1144.4673	11	1145.4829
	SSGDNIFSGFDAQ	1343.563	13	1344.5833
	SSGDNIFSGFDAQQLAE	1784.7853	17	893.412
	STIGAPGSSR	931.4723	10	466.7484
	STIGAPGSSRSE	1147.5469	12	574.7878
	SVLDVSNEANQL	1309.6125	12	655.8195
	SVLDVSNEANQLDF	1549.726	14	775.8779
	SVLDVSNEANQLDFQPR	1930.9385	17	966.4875
	TDENAMINTLAGNL	1491.6875	14	746.8583
	TDENAMINTLAGNLSL	1691.8036	16	846.9185
	TIGAPGSSRSE	1060.5149	11	531.2703
	TLAGNLSLMR	1090.5804	10	546.303
	TLAGNLSLMRAMPVQVIA	1916.0223	18	959.0275
	VAIKTDENAMIN	1333.6548	12	667.84
	VAIKTDENAMINTL	1548.7705	14	775.3987
	VAIKTDENAMINTLAGNL	1902.972	18	952.5043
	VEEGDAFAVPTGF	1337.6139	13	1338.6331
	VEEGDAFAVPTGFGH	1531.6942	15	766.8621
	VEEGDAFAVPTGFGHY	1694.7576	16	848.3951
	VEGGLQALLPPRGQQ	1561.8575	15	781.9439
	VEGGLQALLPPRGQQE	1690.9001	16	846.4661
	VLDVSNEANQL	1200.5986	11	601.3127
	VLDVSNEANQLD	1315.6255	12	658.8261
	VLDVSNEANQLDF	1462.694	13	732.3613

Accession Number	Peptide sequence	Mass	Length	*m/z*
	VLDVSNEANQLDFQPR	1844.8904	16	923.4646
	VPTGFGHY	876.413	8	439.2183
	VQLIRKLQGQND	1410.7943	12	706.4105
	VSNEANQLDFQPR	1517.7109	13	759.8699
	VVQQQGQTVANEE	1428.6844	13	715.3573
	VVQQQGQTVANEEVQ	1654.8274	15	828.4263
	VVQQQGQTVANEEVQQ	1782.886	16	892.4584
	VVRYTIEPNGLLLPS	1670.9243	15	836.4768
	VVVSVLDVSNEANQL	1584.8359	15	793.4321
	YEETICSLR	1078.5294	9	540.2777
	YEETICSLRL	1191.6135	10	596.8196
	YEETICSLRLK	1401.6809	11	701.8533
	YEETICSLRLKQNIG	1813.8879	15	907.9604
	YEETICSLRLKQNIGD	1846.9425	16	924.4875
	YFVQGRGLQG	1123.5774	10	562.8007
	YFVQGRGLQGIL	1349.7455	12	675.887
	YIYNNGNR	1013.4567	8	507.7402
	YLAGNPQN	876.3977	8	877.4124
	YLAGNPQNEF	1151.5247	10	576.7748
	YLAGNPQNEFQ	1279.5833	11	640.8058
	YLAGNPQNEFQQQ	1557.6824	13	779.8556
	YLAGNPQNEFQQQQ	1663.759	14	832.8948
	YLAGNPQNEFQQQQQ	1791.8176	15	896.9253
	YLAGNPQNEFQQQQQQ	1919.8762	16	960.9556
	YLAGNPQNEFQQQQQQ	1920.8602	16	961.4474
	YLAGNPQNEFQQQQQQ	1941.8582	16	971.9464
	YLAGNPQNEFQQQQQQ	1920.8602	16	961.4473
	YLAGNPQNEFQQQQQQ	1920.8602	16	961.4467
	YLAGNPQNEFQQQQQQ	1920.8602	16	961.448
	YVAIKTDENAMIN	1496.718	13	749.374

Accession Number	Peptide sequence	Mass	Length	*m/z*
	YVAIKTDENAMINTL	1710.8499	15	856.4391
F6HZK3	AALIKARDSGFE	1276.6775	12	639.3539
	AALIKARDSGFEY	1439.7408	13	720.8853
	AEAFNVDVQLIR	1373.7302	12	687.8784
	AFNVDVQLIR	1173.6506	10	587.8396
	AGNLSLLR	843.4814	8	422.7519
	AGNPQNEFQQQQQ	1515.6702	13	758.8499
	AGNPQNEFQQQQQQ	1643.7288	14	822.8798
	AMINTLAGNL	1032.5273	10	517.2767
	AMINTLAGNLSL	1232.6434	12	617.3337
	AMINTLAGNLSLLR	1501.8286	14	751.9308
	AMPLQVISSA	1031.5321	10	516.7782
	AMPLQVISSAY	1194.5955	11	598.3105
	AMPLQVISSAYQVSN	1622.7974	15	812.413
	AMPLQVISSAYQVSNN	1736.8403	16	869.4332
	ANQLDFQPR	1087.541	9	544.7853
	ANQLDFQPRR	1243.6421	10	622.8342
	APPGSSRSEY	1049.4778	10	525.7508
	AQQLAEAF	898.416	8	450.2205
	AQQLAEAFNVD	1204.5724	11	603.2983
	AQQLAEAFNVDVQ	1453.6813	13	727.8562
	AVLPPRGQQERGE	1435.7532	13	718.8903
	AVPVGTGH	736.3868	8	737.4017
	AVPVGTGHFIY	1159.6025	11	580.8145
	AVPVGTGHFIYNN	1388.6725	13	695.3508
	AVPVGTGHFIYNNG	1444.7098	14	723.3694
	AVPVGTGHFIYNNGD	1560.7208	15	781.3754
	AVPVGTGHFIYNNGDRQL	1957.9646	18	980.0006
	AYQVSNNQARQL	1391.6793	12	696.8651
	CAGVAVVR	798.4171	8	400.22

Accession Number	Peptide sequence	Mass	Length	*m/z*
	DANQLDFQPR	1202.568	10	602.2974
	DAQQLAEAF	1013.443	9	507.7335
	DAQQLAEAFNVD	1319.5994	12	1320.6228
	DAQQLAEAFNVDVQ	1568.7083	14	785.3668
	DIFSGFDAQQLAEAF	1656.7783	15	829.4086
	DNIFSGFDAQ	1112.4774	10	557.2515
	DNIFSGFDAQQL	1353.6201	12	677.824
	DNIFSGFDAQQLAEAF	1771.8053	16	886.9207
	DQHQKIREVEEGD	1581.7383	13	791.8831
	DRQLIVVSVLDTSND	1672.8632	15	837.4492
	DSGFEYVAIK	1127.5498	10	564.7879
	DTSNDANQLDF	1238.5051	11	1239.5193
	DTSNDANQLDFQPR	1621.6855	14	811.8588
	DTSNDANQLDFQPRRF	1922.8871	16	962.4609
	DVFAVPVGTGHF	1244.6189	12	623.322
	DVFAVPVGTGHFIY	1520.7664	14	761.3908
	DVFAVPVGTGHFIYNNGD	1921.8846	18	961.9569
	EGGGSEGRGQESSG	1274.5123	14	638.2688
	EGGGSEGRGQESSGDNIFSG	1907.7881	20	954.9105
	EGQQQQQEGGGSEGRG	1612.6825	16	807.3608
	EGQQQQQEGGGSEGRGQE	1869.7837	18	935.9081
	EGRGQESSGDNIFSGF	1685.728	16	843.8774
	EGRGQESSGDNIFSGFD	1782.7445	17	892.3871
	EGRGQESSGDNIFSGFDAQ	1981.8402	19	991.9354
	EQGQQGQQGEQGQQ	1510.6396	14	756.3381
	EQGQQGQQGQQ	1196.517	11	599.2727
	EQGQQGQQGQQGQ	1381.597	13	691.8142
	EQGQQGQQGQQGQQ	1509.6556	14	755.8405
	EQGQQGQQGQQGQQG	1566.6771	15	784.3538
	EQGQQGQQGQQGQQGQ	1694.7357	16	848.3826

Accession Number	Peptide sequence	Mass	Length	*m/z*
	EQGQQGQQGQQGQQGQQG	1879.8157	18	940.9254
	ESSGDNIFSGF	1140.4723	11	1141.4949
	ESSGDNIFSGF	1158.4829	11	580.2535
	ESSGDNIFSGFDAQ	1454.595	14	728.3118
	ESSGDNIFSGFDAQQL	1695.7375	16	848.884
	ESTIAPPGSSRSE	1298.6102	13	650.3182
	ESTIAPPGSSRSEY	1479.6841	14	740.855
	EVEEGDVFAVPVGTGH	1640.7682	16	821.4003
	EVEEGDVFAVPVGTGHF	1787.8365	17	894.9324
	FDAQQLAEAF	1138.5294	10	570.2766
	FNVDVQLIR	1102.6135	9	552.3188
	FNVDVQLIR	1103.5975	9	552.8108
	FVQGRGLQGIMIT	1434.7653	13	718.3982
	FYLAGNPQNEF	1298.5931	11	650.3096
	FYLAGNPQNEFQ	1426.6517	12	714.3409
	FYLAGNPQNEFQQQ	1704.7507	14	853.3939
	FYLAGNPQNEFQQQQ	1810.8274	15	906.4309
	FYLAGNPQNEFQQQQQ	1938.886	16	970.4608
	GEGQQQQQEGGGSEGRGQE	1974.8263	19	988.4315
	GFDAQQLAEAF	1195.5509	11	598.7892
	GGSEGRGQESSGDNIFSGF	1886.803	19	944.4203
	GNIVRVEGGLQ	1140.6251	11	571.3252
	GSEGQQQQQEGGGSEGRG	1894.7806	18	948.4084
	GSEGRGQESSGDNIFSGF	1829.7816	18	915.9064
	GSEGRGQESSGDNIFSGFD	1944.8085	19	973.4322
	GYEETICSLRLKQNIGD	1965.9465	17	983.9926
	HNRQESTIAPPGSS	1479.7065	14	740.8672
	HNRQESTIAPPGSSRSE	1851.8823	17	926.9557
	HRSSVTGYD	1020.4625	9	511.2438
	IFSGFDAQQL	1124.5502	10	563.2878

Accession Number	Peptide sequence	Mass	Length	*m/z*
	IFSGFDAQQLAEAF	1564.7173	14	783.373
	IKARDSGFEY	1184.5825	10	593.303
	IQSEAGVTE	932.4451	9	933.4593
	IVRVEGGL	841.5021	8	421.7637
	IVRVEGGLQ	969.5607	9	485.7927
	IVVSVLDTSND	1160.5925	11	1161.613
	IYNNGDRQL	1092.52	9	547.2705
	LAGNPQNEF	989.4454	9	990.4647
	LAGNPQNEFQQQQ	1500.6957	13	751.3615
	LAGNPQNEFQQQQQ	1628.7543	14	815.3921
	LAGNPQNEFQQQQQQ	1756.8129	15	879.4221
	LAGNPQNEFQQQQQQQ	1884.8715	16	943.4539
	LDTSNDANQLDFQPR	1732.8016	15	867.4163
	LLLPSYVNAPQLM	1473.7902	13	737.9103
	LPSYVNAPQLM	1247.6219	11	624.8267
	LRAMPLQVISS	1229.6802	11	615.8528
	LRAMPLQVISSA	1300.7173	12	651.3723
	LRAMPLQVISSAY	1463.7806	13	732.9065
	NDANQLDFQPR	1316.6108	11	659.3191
	NIFSGFDAQ	997.4505	9	499.7371
	NIFSGFDAQQL	1238.5931	11	620.3098
	NIFSGFDAQQLAEA	1509.71	14	755.871
	NIFSGFDAQQLAEAF	1678.7603	15	840.3972
	NIVRVEGGLQ	1083.6036	10	542.814
	NPQNEFQQQQ	1387.6117	11	694.8184
	NPQNEFQQQQQQ	1643.7288	13	822.8789
	NQLDFQPR	1016.5039	8	509.2642
	NRQESTIAPPGSSRSE	1742.8184	16	872.4253
	NRQESTIAPPGSSRSEY	1905.8816	17	953.9541
	NVDVQLIR	955.545	8	478.7863

**Accession Number**	**Peptide sequence**	**Mass**	**Length**	***m/z***
	NVDVQLIRKLQG	1381.8041	12	691.9156
	NVDVQLIRKLQGQND	1738.9326	15	870.4822
	PQNEFQQQQQQ	1402.6113	11	702.318
	PRGQQERGEQQQD	1582.7084	13	792.3716
	PSYVNAPQL	987.5025	9	988.5201
	QEGGGSEGRGQE	1172.4694	12	587.2489
	QEGGGSEGRGQES	1259.5013	13	630.7654
	QEGGGSEGRGQESSG	1403.5549	15	702.7936
	QEGGGSEGRGQESSGD	1518.5818	16	760.3067
	QEGGGSEGRGQESSGDN	1632.6248	17	817.3274
	QEGGGSEGRGQESSGDNI	1745.7089	18	873.868
	QESSGDNI	831.3246	8	832.3392
	QESSGDNIFSGF	1268.5309	12	635.2784
	QESSGDNIFSGFD	1384.5419	13	693.2862
	QESSGDNIFSGFDAQ	1583.6376	15	792.8339
	QESSGDNIFSGFDAQQL	1824.7802	17	913.407
	QESTIAPPGSSRSE	1427.6528	14	714.8425
	QESTIAPPGSSRSEY	1590.7162	15	796.3741
	QGQQFRGDQHQ	1310.5752	11	656.3005
	QGQQGEQGQQGQQG	1439.6025	14	720.8165
	QGQQGEQGQQGQQGQQG	1752.7412	17	877.3868
	QGQQGQQGQQFRGD	1543.6764	14	772.8519
	QGQQGQQGQQGQQFRGD	1856.8149	17	929.4255
	QHQKIREVE	1148.5938	9	575.3095
	QHQKIREVEEG	1334.6578	11	668.3425
	QHQKIREVEEGD	1449.6848	12	725.8588
	QHQKIREVEEGDVF	1695.8217	14	848.9283
	QKIREVEEGD	1184.5673	10	593.2968
	QLAEAFNVD	1005.4767	9	1006.4948
	QLDFQPR	885.4344	7	443.7302

**Accession Number**	**Peptide sequence**	**Mass**	**Length**	***m/z***
	QQEGGGSEGRGQE	1300.528	13	651.2756
	QQEGGGSEGRGQES	1387.5599	14	694.7953
	QQEGGGSEGRGQESS	1474.592	15	738.3078
	QQEGGGSEGRGQESSG	1531.6135	16	766.8219
	QQEGGGSEGRGQESSGD	1646.6404	17	824.3361
	QQEGGGSEGRGQESSGDNI	1873.7675	19	937.8993
	QQERGEQQQD	1227.5116	10	614.7725
	QQFRGDQHQK	1253.5901	10	418.875
	QQGEQGQQGQQ	1197.501	11	599.7711
	QQGEQGQQGQQG	1254.5225	12	628.2741
	QQGEQGQQGQQGQ	1382.5811	13	692.3049
	QQGQQFRGD	1045.4576	9	523.7405
	QQGQQFRGDQH	1310.5752	11	656.3005
	QQGQQFRGDQHQ	1438.6338	12	720.3302
	QQGQQGQQFR	1186.548	10	594.2872
	QQGQQGQQFRGD	1358.5963	12	680.3123
	QQGQQGQQFRGDQH	1623.7139	14	812.8681
	QQGQQGQQFRGDQHQ	1751.7723	15	876.9001
	QQGQQGQQGQQFRGD	1671.735	15	836.8829
	QQGQQGQQGQQGQQFR	1812.8252	16	907.4277
	QQQEGGGSEGRG	1171.4854	12	586.7562
	QQQEGGGSEGRGQE	1428.5865	14	715.3077
	QQQEGGGSEGRGQES	1515.6185	15	758.8244
	QQQEGGGSEGRGQESSG	1659.672	17	830.8531
	QQQEGGGSEGRGQESSGD	1774.699	18	888.3657
	QQQQEGGGSEGR	1242.5225	12	622.2749
	QQQQEGGGSEGRGQE	1556.6451	15	779.3378
	QQQQEGGGSEGRGQES	1643.6771	16	822.8529
	QQQQEGGGSEGRGQESSG	1787.7306	18	894.8806
	QQQQEGGGSEGRGQESSGD	1902.7576	19	952.3929

Accession Number	Peptide sequence	Mass	Length	*m/z*
	QQQQQEGGGSEGR	1370.5811	13	686.3057
	QQQQQEGGGSEGRG	1427.6025	14	714.8164
	QQQQQEGGGSEGRGQ	1555.6611	15	778.8478
	QQQQQEGGGSEGRGQE	1684.7037	16	843.3669
	QQQQQEGGGSEGRGQES	1771.7357	17	886.884
	QQQQQEGGGSEGRGQESSG	1915.7892	19	958.9115
	RFYLAGNPQN	1178.5833	10	590.3042
	RFYLAGNPQNE	1307.6259	11	654.8259
	RFYLAGNPQNEF	1455.6782	12	728.8551
	RFYLAGNPQNEFQ	1583.7368	13	792.8829
	RFYLAGNPQNEFQQ	1711.7954	14	856.9129
	RFYLAGNPQNEFQQQ	1839.854	15	920.9435
	RFYLAGNPQNEFQQQQ	1966.9285	16	984.4823
	RGQESSGDNIF	1208.5421	11	605.2826
	RGQESSGDNIFSG	1352.5956	13	677.3109
	RGQESSGDNIFSGF	1499.6641	14	750.8472
	RGQESSGDNIFSGFD	1614.6909	15	808.3605
	RIQSEAGVT	959.5036	9	480.7648
	RIQSEAGVTEV	1187.6146	11	594.8206
	RIQSEAGVTEVF	1334.683	12	668.3547
	RIQSEAGVTEVFD	1449.71	13	725.8697
	RQESTIAPPGSSR	1384.7058	13	693.3662
	RQESTIAPPGSSRS	1471.7379	14	736.8821
	RQESTIAPPGSSRSE	1601.7645	15	801.8959
	RQESTIAPPGSSRSEY	1763.8438	16	882.9363
	RQLIVVSVLDTSND	1557.8362	14	779.932
	SEGQQQQQEGGGSEGR	1660.7037	16	831.3703
	SEGQQQQQEGGGSEGRG	1717.7251	17	859.8801
	SEGQQQQQEGGGSEGRGQ	1846.7677	18	924.4038
	SEGQQQQQEGGGSEGRGQE	1975.8103	19	988.9237

Accession Number	Peptide sequence	Mass	Length	*m/z*
	SEGRGQESSGDNI	1334.5698	13	668.2983
	SEGRGQESSGDNIFSG	1625.6917	16	813.8605
	SEGRGQESSGDNIFSGF	1772.7601	17	887.3965
	SEGRGQESSGDNIFSGFD	1887.7871	18	944.9106
	SGDNIFSGFD	1057.4352	10	1058.4517
	SGDNIFSGFDAQ	1256.5309	12	1257.5485
	SGDNIFSGFDAQQLAEAF	1915.8588	18	958.9465
	SGFDAQQLAEAF	1282.5829	12	1283.6012
	SGFEYVAIK	1012.5229	9	507.2737
	SNDANQLDFQPR	1403.6429	12	702.8353
	SSGDNIFSGF	1051.4222	10	526.7214
	SSGDNIFSGFD	1144.4673	11	1145.4829
	SSGDNIFSGFDAQ	1343.563	13	1344.5833
	SSGDNIFSGFDAQQLAE	1784.7853	17	893.412
	SSVTGYDLPILR	1319.7085	12	660.8659
	STIAPPGSS	815.4025	9	816.4166
	STIAPPGSSRSE	1187.5782	12	594.8035
	STIAPPGSSRSEY	1350.6415	13	676.3353
	SVLDTSNDANQL	1275.5942	12	638.8114
	SVLDTSNDANQLDF	1537.6896	14	769.859
	SVLDTSNDANQLDFQPR	1919.8861	17	960.9592
	SVNAPQLM	950.4531	8	951.472
	THENAMINTLAGNL	1513.7195	14	757.8754
	THENAMINTLAGNLSL	1713.8356	16	857.9336
	TIAPPGSSRSE	1100.5461	11	551.2853
	TLAGNLSLLR	1056.6292	10	529.327
	TSNDANQLDFQPR	1505.6746	13	753.8521
	VAIKTHENAM	1128.5597	10	565.2928
	VAIKTHENAMIN	1355.6868	12	678.8562
	VAIKTHENAMINTL	1569.8185	14	785.9242

Accession Number	Peptide sequence	Mass	Length	*m/z*
	VEEGDVFAVPVGTGH	1511.7256	15	756.8781
	VEEGDVFAVPVGTGHF	1658.7939	16	830.4111
	VEEGDVFAVPVGTGHFIY	1934.9414	18	968.49
	VEGGLQAVLPPRGQQ	1547.8419	15	774.9348
	VEGGLQAVLPPRGQQE	1676.8845	16	839.4578
	VFAVPVGTGH	982.5236	10	492.274
	VFAVPVGTGHFIY	1405.7394	13	703.8855
	VFAVPVGTGHFIYN	1520.7664	14	761.3978
	VFAVPVGTGHFIYNN	1634.8092	15	818.4186
	VFAVPVGTGHFIYNNG	1691.8307	16	846.9319
	VFAVPVGTGHFIYNNGD	1807.8417	17	904.9357
	VISSAYQVSNNQAR	1535.7692	14	768.8993
	VLDTSNDAN	948.4036	9	949.4179
	VLDTSNDANQL	1188.5623	11	595.2943
	VLDTSNDANQLD	1325.5712	12	663.7998
	VLDTSNDANQLDF	1450.6576	13	726.3432
	VLDTSNDANQLDFQPR	1831.87	16	916.9525
	VNAPQLMY	950.4531	8	476.2403
	VPVGTGHF	812.4181	8	407.2212
	VQGRGLQGIMIT	1287.6969	12	644.8618
	VQLIRKLQGQND	1410.7943	12	706.4105
	VVQQQGQNVFNEEVQQ	1871.9126	16	936.9728
	YEETICSLR	1112.5172	9	557.2707
	YEETICSLRL	1191.6135	10	596.8196
	YEETICSLRLK	1401.6809	11	701.8533
	YEETICSLRLKQNIG	1813.8879	15	907.9604
	YEETICSLRLKQNIGD	1928.9149	16	965.4749
	YFVQGRGLQG	1123.5774	10	562.8007
	YFVQGRGLQGIMIT	1597.8286	14	799.9272
	YLAGNPQN	876.3977	8	877.4124

Accession Number	Peptide sequence	Mass	Length	*m/z*
	YLAGNPQNEF	1151.5247	10	576.7748
	YLAGNPQNEFQ	1279.5833	11	640.8058
	YLAGNPQNEFQQQ	1557.6824	13	779.8556
	YLAGNPQNEFQQQQ	1663.759	14	832.8948
	YLAGNPQNEFQQQQQ	1792.8016	15	897.4176
	YLAGNPQNEFQQQQQQ	1919.8762	16	960.9556
	YQVSNNQAR	1078.5155	9	540.2706
A5AEI7	ERYIRQQAEQQQGGQG	1856.8877	16	929.4574
	ERYIRQQAEQQQGGQGD	1971.9147	17	986.9742
	IAQKLPERCGSGQA	1504.7303	14	753.3781
	IRQQAEQQQGGQ	1369.6698	12	685.8503
	IRQQAEQQQGGQG	1427.6753	13	714.8534
	IRQQAEQQQGGQGD	1542.7023	14	772.3669
	IVQRQQGQGQGQ	1326.6641	12	664.3474
	IVQRQQGQGQGQG	1383.6854	13	692.8584
	IVQRQQGQGQGQGQ	1510.76	14	756.3967
	IVQRQQGQGQGQGQG	1568.7655	15	785.3999
	IVQRQQGQGQGQGQGQ	1695.8401	16	848.9377
	IVQRQQGQGQGQGQGQG	1752.8616	17	877.4474
	IVQRQQGQGQGQGQGQGQ	1880.9202	18	941.4788
	IVQRQQGQGQGQGQGQGQG	1937.9415	19	969.9896
	QAEQQQGGQG	1012.421	10	507.2236
	QAEQQQGGQGV	1127.4479	11	564.7378
	QGQGQGQGQGQG	1111.4642	12	556.7456
	QGQGQGQGQGQGQG	1296.5443	14	649.2845
	QGQGQGQGQGQGQGQG	1481.6244	16	741.8253
	QGQGQGQGQGQGQGQGQG	1666.7043	18	834.3776
	QGQGQGQGQGQGQGQGQRE	1894.8267	19	948.4321
	QGQGQGQGQGQGQGQR	1580.704	16	791.3691
	QGQGQGQGQGQGQGQRE	1709.7466	17	855.8893

Accession Number	Peptide sequence	Mass	Length	*m/z*
	QGQGQGQGQGQGQGQREQ	1837.8052	18	919.9198
	QGQGQGQGQGQGQGQREQQ	1965.8638	19	983.9487
	QGQGQGQGQGQGQR	1395.6239	14	698.8274
	QGQGQGQGQGQGQRE	1524.6665	15	763.3495
	QGQGQGQGQGQGQREQ	1652.7251	16	827.3799
	QGQGQGQGQGQGQREQQ	1780.7837	17	891.4086
	QGQGQGQGQGQGQREQQQ	1908.8423	18	955.4395
	QGQGQGQGQGQR	1210.5439	12	606.2869
	QGQGQGQGQGQRE	1339.5864	13	670.8076
	QGQGQGQGQGQREQ	1467.645	14	734.8381
	QGQGQGQGQGQREQQ	1595.7036	15	798.8674
	QGQGQGQGQGQREQQQ	1723.7622	16	862.8976
	QGQGQGQGQGQREQQQE	1852.8048	17	927.4185
	QGQGQGQGQREQ	1282.5651	12	642.2974
	QGQGQGQGQREQQ	1410.6237	13	706.3275
	QGQGQGQGQREQQQE	1667.7247	15	834.8787
	QGQGQGQREQQ	1225.5436	11	613.7872
	QGQGQGQREQQQ	1353.6022	12	677.8149
	QGQGQGQREQQQE	1482.6448	13	742.3372
	QIAQKLPE	908.4967	8	455.2608
	QIAQKLPER	1064.5978	9	533.3103
	QIVQRQQGQ	1066.552	9	534.2874
	QIVQRQQGQGQ	1268.6586	11	635.3444
	QIVQRQQGQGQG	1308.6534	12	655.3391
	QIVQRQQGQGQGQ	1454.7225	13	728.3762
	QIVQRQQGQGQGQG	1510.76	14	756.3954
	QIVQRQQGQGQGQGQ	1621.7921	15	811.9104
	QIVQRQQGQGQGQGQG	1678.8135	16	840.4214
	QIVQRQQGQGQGQGQGQ	1824.8827	17	913.4513
	QIVQRQQGQGQGQGQGQG	1863.8936	18	932.9633

Accession Number	Peptide sequence	Mass	Length	*m/z*
	QQAEQQQGGQG	1140.4796	11	571.2536
	QQAEQQQGGQGD	1255.5065	12	628.7674
	QQIQGQQF	958.4508	8	959.4698
	QQQEQQWLR	1225.584	9	613.8057
	QQQQEQQWLR	1353.6426	10	677.8383
	QQQQEQQWLRQ	1481.7012	11	741.8655
	REQQQEMMQIA	1422.6232	11	712.3252
	RQQGQGQGQGQGQGQGQGQ	1912.8372	19	957.4418
	RYIRQQAEQQQGGQGD	1860.8827	16	931.4576
	YIRQQAEQQ	1162.573	9	582.3006
	YIRQQAEQQQ	1290.6316	10	646.3292
	YIRQQAEQQQG	1348.6371	11	675.3312
	YIRQQAEQQQGG	1404.6746	12	703.3516
	YIRQQAEQQQGGQ	1532.7332	13	767.3807
	YIRQQAEQQQGGQG	1611.7366	14	806.8812
	YIRQQAEQQQGGQGD	1732.7765	15	867.4011
F6HI56	AFSIPAREVD	1103.561	10	552.7928
	AFSIPAREVDEVF	1478.7405	13	740.3848
	ANEFQQLQDMD	1353.5507	11	677.7899
	ANEFQQLQDMDIAVSY	1886.8356	16	944.4352
	ANPQTFVLPAHLDAE	1621.8099	15	811.9166
	ASEQQIQALSQREE	1615.7802	14	808.8993
	ASEQQIQALSQREES	1702.8121	15	852.4215
	ASEQQIQALSQREESG	1759.8336	16	880.9344
	ASEQQIQALSQREESGM	1906.869	17	954.4497
	ATKIAVVVKGEGYME	1609.8385	15	805.9402
	DQGRQQEQGQEQEQ	1682.7244	14	842.3784
	EANPQTFVLPAHL	1417.7354	13	1418.7556
	EANPQTFVLPAHLDAE	1750.8525	16	876.4426
	EFQPFYGPAGEN	1336.5724	12	669.3

Accession Number	Peptide sequence	Mass	Length	*m/z*
	EFQPFYGPAGENP	1433.6251	13	717.8272
	EFQPFYGPAGENPQS	1648.7157	15	825.3741
	EFQPFYGPAGENPQSF	1795.7842	16	898.908
	ELAFSIPAREVD	1345.6877	12	673.8573
	ELAFSIPAREVDEVF	1720.8672	15	861.4516
	ENPQTFVLPAHLD	1421.7302	13	711.8792
	EQGRQQEQGQEQ	1425.6233	12	713.8269
	EQGRQQEQGQEQE	1554.6659	13	778.3495
	EQGRQQEQGQEQEQE	1811.767	15	906.8989
	EQGRQQEQGQEQEQEQ	1939.8256	16	970.9287
	EQQQQSASPHYQRL	1680.7968	14	841.4122
	ETNEGRIK	927.4774	8	464.7511
	FQPFYGPAGENPQSF	1684.7521	15	843.3925
	FSIPAREVDEVF	1407.7034	12	704.8665
	FVLPAHLDAE	1110.5709	10	556.299
	FVVPAGHP	822.4388	8	823.4499
	FVVPAGHPLIV	1147.6753	11	574.851
	HVSQQQQGQGQSTGEQR	1881.8678	17	628.3059
	IAVVVKGEGY	1033.5808	10	517.8023
	IAVVVKGEGYMEM	1456.6942	13	729.3619
	KGVIVKASEQQIQA	1497.8514	14	749.9382
	LEANPQTFVL	1130.5972	10	566.3118
	LEANPQTFVLPAHL	1548.83	14	775.4297
	LEANPQTFVLPAHLD	1663.8569	15	832.9427
	LLSKEPSISN	1086.592	10	544.3073
	LLSSALKVEQD	1201.6554	11	601.8397
	NITKGSMEGP	1049.47	10	525.7468
	PAGENPQSF	945.4192	9	946.4358
	PAGENPQSFYR	1264.5836	11	633.3046
	PDEFQPFYGPA	1266.5557	11	1267.5708

Accession Number	Peptide sequence	Mass	Length	*m/z*
	PDEFQPFYGPAGENP	1663.7153	15	832.8738
	PDEFQPFYGPAGENPQS	1878.806	17	940.4197
	PHVSQQQQGQGQSTGEQR	1978.9205	18	660.6537
	PQTFVLPAHL	1121.6233	10	561.8256
	PQTFVLPAHLDAE	1436.73	13	719.3757
	PYVFQDQHY	1195.5298	9	598.7781
	QGQGQSTGEQR	1157.5061	11	579.7666
	QGRQQEQGQEQ	1297.5647	11	649.797
	QGRQQEQGQEQE	1426.6073	12	714.3192
	QGRQQEQGQEQEQ	1554.6659	13	778.3572
	QGRQQEQGQEQEQE	1683.7085	14	842.8687
	QGRQQEQGQEQEQEQG	1868.7885	16	935.4088
	QNKGVIVKASEQQIQAL	1836.0105	17	919.0192
	QPFYGPAGENPQSF	1520.6571	14	761.3423
	QQGQGQSTGEQR	1285.5647	12	643.7977
	QQGQGQSTGEQRR	1441.6658	13	721.8524
	QQNKGVIVKA	1066.6135	10	534.3187
	QQQQSASPHYQR	1439.6542	12	720.8414
	QQQQSASPHYQRL	1552.7383	13	777.3845
	QQQQSASPHYQRLS	1639.7703	14	820.9
	QQQSASPHY	1027.4359	9	1028.4534
	QQQSASPHYQR	1311.5956	11	656.8138
	QQQSASPHYQRL	1424.6797	12	713.3536
	QQQSASPHYQRLS	1511.7117	13	756.8702
	QQSASPHY	899.3773	8	900.3906
	QQSASPHYQR	1183.537	10	592.7805
	QRGEEGKGGGGQSGE	1414.6073	15	708.3179
	SEELLSSALKVEQD	1546.7726	14	774.4017
	SEQQIQALSQREES	1631.775	14	816.9021
	SEQQIQALSQREESG	1688.7965	15	845.4141

Accession Number	Peptide sequence	Mass	Length	*m/z*
	SEQQIQALSQREESGM	1835.8319	16	918.9309
	SIPAREVDEVF	1260.635	11	631.3317
	SKEPSISN	861.408	8	431.7161
	SLAGDKNIVNAL	1213.6666	12	607.8459
	SMEGPFFNTR	1200.5233	10	601.2745
	SNITKGSMEGP	1136.502	11	569.2635
	SNITKGSMEGPF	1283.5703	12	642.7993
	SNITKGSMEGPFFNT	1645.7294	15	823.8793
	SQREESGMFPFPFG	1630.7085	14	816.3719
	SQREESGMFPFPFGSTE	1947.8308	17	974.9282
	SYSNITKGSMEGP	1386.5973	13	694.314
	TFVLPAHLDAE	1211.6187	11	606.8224
	VASGRGTLSLVSQG	1330.7205	14	666.3735
	VSQQQQGQGQSTGEQR	1744.8088	16	873.4198
	VSYSNITKGSMEG	1388.6129	13	695.3203
	VSYSNITKGSMEGP	1485.6656	14	743.847
	VVVKGEGYM	996.495	9	499.2609
	VVVKGEGYME	1125.5376	10	563.7812
	VVVKGEGYMEM	1272.573	11	637.2995
	VVVKGEGYMEMA	1343.6101	12	672.8182
	VVYVASGRGTLSL	1320.7401	13	661.3814
	YGPAGENPQSF	1165.5039	11	583.7644
	YGPAGENPQSFYR	1484.6683	13	743.3481
	YTASLETNEGR	1239.5731	11	620.799
	YTASLETNEGRIK	1480.7522	13	741.3906
A5C7L5	AANPISGE	757.3606	8	758.3744
	AANPISGETA	929.4454	10	930.4615
	AANPISGETAFG	1133.5353	12	567.78
	AANPISGETAFGE	1262.5779	13	632.3024
	AANPISGETAFGEL	1375.6619	14	688.8433

Accession Number	Peptide sequence	Mass	Length	*m/z*
	AANPISGETAFGELA	1446.699	15	724.3646
	AANPISGETAFGELAQ	1574.7576	16	788.3943
	AANPISGETAFGELAQD	1689.7845	17	845.9081
	AANPISGETAFGELAQDVL	1915.9163	19	958.992
	AQDVLSIPSTD	1166.5431	11	584.2855
	AQDVLSIPSTDGSSLGQL	1808.8768	18	905.4556
	AQKGFPPAVQG	1098.5822	11	550.3036
	ASHFGFQQ	920.4141	8	921.436
	ATKMTATQIGPE	1262.6177	12	632.3212
	ATKMTATQIGPEVA	1432.7231	14	717.3682
	ATKMTATQIGPEVAN	1547.7501	15	774.8867
	ATKMTATQIGPEVANID	1774.8771	17	888.4497
	ATQIGPEVAN	999.4872	10	1000.5074
	ATQIGPEVANID	1226.6143	12	614.3217
	AVQGVVGLGHTS	1123.5985	12	562.812
	DATKMTATQIGPE	1377.6445	13	689.834
	DATKMTATQIGPEVAN	1661.7931	16	831.9081
	DLARSKLGF	1005.5607	9	503.7924
	DLARSKLGFSSSL	1379.7408	13	690.8844
	DSVSSRPNALVLL	1369.7565	13	685.8921
	DVLSIPSTDGSSLGQL	1587.7992	16	794.9158
	DVNGRSLWVD	1160.5461	10	581.2867
	EAPYRLHPGID	1248.6251	11	625.3262
	EAPYRLHPGIDV	1347.6935	12	674.8623
	EAPYRLHPGIDVSH	1571.7844	14	524.9401
	EAPYRLHPGIDVSHP	1668.8372	15	557.2912
	EATNLHVVD	978.4771	9	979.4945
	EHSIYQTF	1005.4556	8	1006.4706
	ERVVPVNPAL	1074.6185	10	1075.6399
	ERVVPVNPALLN	1320.7401	12	661.3829

**Accession Number**	**Peptide sequence**	**Mass**	**Length**	***m/z***
	FFVGSAQTS	942.4447	9	943.4607
	FVDGGSNP	791.345	8	792.3583
	FVDGGSNPKAPII	1313.6979	13	657.8621
	HVVDIQK	837.4708	7	419.7479
	INERVVPVNPALLN	1547.8671	14	774.9467
	IPSTDGSSL	875.4236	9	876.4356
	IPSTDGSSLG	932.4451	10	933.4595
	IPSTDGSSLGQ	1060.5037	11	1061.5217
	IPSTDGSSLGQL	1173.5876	12	1174.6057
	LGEAPYRLHPGID	1436.7412	13	719.3849
	LHPGIDVSHP	1070.5509	10	536.2872
	LTNDSVSSRPN	1189.5575	11	595.7906
	LTSPLNHGVLF	1197.6394	11	599.8328
	LVSKNEATNL	1088.5713	10	545.2965
	LVSKNEATNLHVVD	1537.8101	14	769.9193
	NFFVGSAQTS	1056.4875	10	1057.5063
	NSMVQPRPGV	1099.5444	10	550.7839
	NSMVQPRPGVW	1286.6078	11	644.3169
	PGIDVSHP	820.4079	8	411.2156
	PGIDVSHPLG	990.5134	10	496.2682
	PISGETAFGE	1006.4607	10	1007.4775
	PISGETAFGEL	1119.5448	11	1120.5645
	PISGETAFGELAQ	1318.6405	13	660.3341
	PISGETAFGELAQD	1433.6674	14	717.8481
	PISGETAFGELAQDVL	1645.8198	16	823.925
	PKAPIILGSY	1057.6172	10	529.8204
	PKAPIILGSYQLED	1542.8293	14	772.431
	PTQLASHF	899.4501	8	450.7372
	PTQLASHFGF	1103.5399	10	552.7828
	PTQLASHFGFQQ	1359.6571	12	680.8406

Accession Number	Peptide sequence	Mass	Length	*m/z*
	PTQLASHFGFQQK	1487.7521	13	496.9291
	PVSGETAFGELAQD	1419.6517	14	710.8395
	QKGFPPAVQG	1010.5185	10	1011.5387
	QLEDNLLQFDLAR	1556.7834	13	779.4064
	RLHPGIDVSHP	1226.652	11	409.8954
	SIPSTDGSSLGQ	1147.5356	12	574.782
	SIPSTDGSSLGQL	1242.6091	13	622.3177
	SMVQPRPGVW	1171.5808	10	586.8027
	SNPKAPIILGSYQLED	1743.9043	16	872.9701
	STDGSSLGQ	850.3668	9	426.1957
	TDGSSLGQV	858.4083	9	859.4248
	VDGGSNPKAP	940.4614	10	471.2428
	VDGGSNPKAPII	1166.6295	12	584.3278
	VDGGSNPKAPIIL	1279.7135	13	640.8708
	VDGGSNPKAPIIL	1261.703	13	631.8648
	VDGGSNPKAPIILGS	1423.7671	15	712.8949
	VLLVSKNEATNL	1299.7397	12	650.8834
	VLSIPSTD	830.4385	8	831.455
	VLSIPSTDGSSLGQ	1359.6881	14	680.8581
	VLSIPSTDGSSLGQL	1473.7562	15	737.8932
	VQPIAPFGL	940.5381	9	941.5564
	VSHPLGSTPLSIS	1293.6929	13	647.8609
	VSHPLGSTPLSISRE	1577.8525	15	526.963
	VSHPLGSTPLSISREGEY	1927.9639	18	965.0019
	VSKNEATNL	974.5032	9	488.2641
	VSKNEATNLHVVD	1424.726	13	713.377
	VVGLGHTS	768.413	8	769.4272
	VVGLGHTSI	881.4971	9	441.7598
	VVGLGHTSIA	952.5342	10	477.2816
	VVGLGHTSIAL	1065.6182	11	533.821

Accession Number	Peptide sequence	Mass	Length	*m/z*
	VVGLGHTSIALPTQL	1504.8613	15	753.4465
	VVGLGHTSIALPTQLA	1575.8984	16	788.9644
F6H566	AASALPTKCGV	1016.5325	11	509.2773
	AASALPTKCGVQ	1192.5758	12	597.3007
	AASALPTKCGVQIGIPIS	1690.9617	18	846.4978
	DAASALPTKCGVQ	1225.6302	13	613.8279
	FQNIKED	892.429	7	447.2261
	IGIPISM	745.4044	7	746.4217
	IGIPISMT	846.4521	8	847.468
	IKEDAASALPTK	1242.6819	12	622.3536
	IKEDAASALPTKC	1393.6759	13	697.8508
	IKEDAASALPTKCG	1450.6973	14	726.3597
	IKEDAASALPTKCGV	1549.7657	15	775.8975
	IKEDAASALPTKCGVQ	1595.8518	16	798.9403
	KLLTPTTTD	988.5441	9	495.2846
	LKLLTPTTTD	1101.6282	10	551.8262
	LLTPTTTD	860.4491	8	861.465
	LTGGGNPAAPCCN	1172.4828	13	1173.4773
	LTPTTTDRRD	1174.5942	10	588.313
	NLKLLTPTTTD	1215.671	11	608.8474
	PYLTGGGN	777.3657	8	778.3798
	PYLTGGGNP	874.4185	9	875.4354
	PYLTGGGNPA	945.4556	10	946.4723
	PYLTGGGNPAA	1016.4927	11	509.2574
	PYLTGGGNPAAP	1113.5454	12	1114.5664
	PYLTGGGNPAAPCCN	1432.5989	15	1433.5919
	PYLTGGGNPAAPCCNGVQNL	1943.8744	20	972.9426
	SKFQNIKED	1107.556	9	554.7905
	VKAAASKFQN	1063.5662	10	532.7955
	VQIGIPISM	972.5314	9	973.5499

Accession Number	Peptide sequence	Mass	Length	*m/z*
	VQIGIPISMT	1073.5791	10	1074.6
	YLTGGGNPA	848.4028	9	849.4176
D7U302	AEAFNVDAQLIR	1359.7146	12	680.8716
	ANQLDFQPR	1087.541	9	544.7853
	ANQLDFQPRR	1243.6421	10	622.8342
	AVFPQRGQEEQGSEQQE	1945.8766	17	973.9592
	CAGVAVVR	798.4171	8	400.22
	DANQLDFQPR	1202.568	10	602.2974
	DISNDANQLDFQPR	1631.7539	14	544.93
	DQHQKIREVQEGD	1581.7383	13	791.8831
	GNIVRVEGGLQ	1140.6251	11	571.3252
	IQSEAGVTE	932.4451	9	933.4593
	IVRVEGGL	841.5021	8	421.7637
	IVRVEGGLQ	969.5607	9	485.7927
	NDANQLDFQPR	1316.6108	11	659.3191
	NIVRVEGGLQ	1083.6036	10	542.814
	NQLDFQPR	1016.5039	8	509.2642
	NVDAQLIR	927.5137	8	464.7686
	NVDAQLIRKLQGQND	1725.9009	15	863.9669
	PQRGQEEQGSEQQE	1628.7026	14	815.3704
	PQRGQEEQGSEQQED	1743.7295	15	872.8845
	QLDFQPR	885.4344	7	443.7302
	RIQSEAGVT	959.5036	9	480.7648
	SNDANQLDFQPR	1403.6429	12	702.8353
	SVIDISNDANQLDFQPR	1930.9385	17	966.485
F6HMH7	ATNFVPGK	832.4443	8	417.2346
	AWAYATNFVPGK	1323.6611	12	662.8443
	AYATNFVPGK	1066.5447	10	534.2838
	PLYPGGSF	836.4068	8	837.424
	PLYPGGSFD	951.4338	9	952.4517

Accession Number	Peptide sequence	Mass	Length	*m/z*
	PLYPGGSFDPL	1161.5706	11	1162.5908
	PLYPGGSFDPLG	1218.592	12	1219.613
	PLYPGGSFDPLGLAD	1571.6594	15	786.8449
	PLYPGGSFDPLGLADD	1654.749	16	828.3914
	PPSYLTGEFPGD	1300.5587	12	651.2929
	PPSYLTGEFPGDYGWD	1799.7678	16	900.8993
	PVNNNAWAYATNFVPGK	1863.8792	17	932.953
	RVLYLGPLSGD	1188.6503	11	595.3376
	YATNFVPGK	995.5076	9	498.7657
	YGWDTAGLSAD	1154.488	11	1155.5073
A5AG74	AAEQIGTRGTQG	1187.5895	12	594.8076
	AASGLVASGGFGVSA	1249.6302	15	625.8288
	AEQIGTRGTQG	1116.5524	11	559.2896
	AHQDQPQKTQL	1334.6578	11	668.3433
	AHQDQPQKTQLS	1421.6899	12	711.8583
	AHQDQPQKTQLSY	1584.7532	13	793.3893
	ASGLVASGGFGVSA	1178.5931	14	590.3096
	ERAAEQIGTR	1111.5734	10	556.7981
	ERAAEQIGTRGTQG	1454.7225	14	728.375
	EVKERAAEQIGTR	1467.7793	13	490.2709
	EVKERAAEQIGTRGTQG	1810.9285	17	604.6544
	FLAASGLVASGGFGVSAV	1650.8617	18	826.4457
	LAASGLVASGGFGVS	1291.6771	15	646.8527
	LAASGLVASGGFGVSA	1362.7142	16	682.3712
F6I0M9	ADLRDEHGN	1067.4631	9	534.7437
	ADLRDEHGNPIQL	1518.7427	13	760.3735
	ADLRDEHGNPIQLT	1619.7903	14	810.9095
	ADLRDEHGNPIQLTD	1734.8173	15	868.4384
	AEPAEGGGGEVH	1108.4785	12	555.252
	PVQLTDEHGN	1109.4989	10	555.7619

Accession Number	Peptide sequence	Mass	Length	*m/z*
	QAVHGGAPVA	888.4453	10	889.4607
	QAVHGGAPVAA	959.4824	11	960.4927
	QAVHGGAPVAAEPAEGGGGEVH	1978.9132	22	990.4692
A5ASF5	AATAATAGGSL	889.4505	11	890.4656
	AATAATAGGSLLV	1223.5798	13	612.7956
	AEIQPPHHI	1082.5509	9	542.2887
	AEIQPPHHIL	1195.6349	10	598.8314
	AEIQPPHI	969.4668	8	970.4818
	AEQFGQQHTGQQGT	1515.6702	14	758.8499
	ATAATAGGSL	818.4134	10	819.4283
	AVKAATAATAGGSL	1187.651	14	594.8384
	DRAEQFGQQHTGQQGT	1786.7982	16	894.4142
	QAVKAATAATAGGSL	1298.683	15	650.3558
	RAEQFGQQHTGQQG	1570.7236	14	786.3775
	RAEQFGQQHTGQQGT	1671.7714	15	836.9
	TAATAGGSL	747.3763	9	748.3915
	VKAATAATAGGSL	1116.6139	13	559.3181
A5B9G2	AEHQQRPG	964.4362	8	483.231
	AEHQQRPGF	1110.5206	9	556.2736
	AEHQQRPGFMP	1354.6088	11	678.3177
	AEHQQRPGFMPE	1483.6514	12	742.8403
	AKEVGHGIQGKAQE	1450.7528	14	484.5976
	EVGHGIQGKAQETTRT	1692.8544	16	847.4399
	HGIQGKAQETTRT	1426.7164	13	476.5846
	VGHGIQGKAQE	1122.5781	11	562.3031
	VGHGIQGKAQETTRT	1581.8223	15	791.9271
F6HYK6	AASKAEGKAIGID	1271.6721	13	636.8491
	AASKAEGKAIGIDLG	1441.7776	15	721.9041
	AASKAEGKAIGIDLGTTY	1806.9363	18	904.4855
	AGYGGSTGSGGGAGPK	1279.5792	16	640.8054

Accession Number	Peptide sequence	Mass	Length	*m/z*
	AGYGGSTGSGGGAGPKIE	1521.7059	18	761.8664
	GAGYGGSTGSGGGAGPKIEEVD	1903.8547	22	952.9512
	MPGAGYGGSTGSGGGAGPKIE	1822.8156	21	912.4053
F6GTY5	EERQESEGGEHE	1396.5491	12	699.2887
	EGGGEERQESEGGEHEL	1809.7401	17	905.8855
	ELVEKQLKIGDL	1365.7867	12	683.9069
	FLGGGTHPTSIL	1198.6346	12	600.3306
	IASRTGPFEF	1123.5662	10	562.7963
	IDMSESLKMDTFQSF	1809.78	15	905.9039
	KKQPVEPTEPYNL	1541.809	13	771.917
	KKQPVEPTEPYNLY	1704.8722	14	853.4519
	KKQPVEPTEPYNLYD	1819.8992	15	910.9662
	KQPVEPTEPYNLYD	1691.8042	14	846.9182
	LTAGSMMAPHLNPTATEIG	1942.9128	19	972.4725
	SQREAVILPSADVSPPD	1779.9003	17	890.9678
	SVNLTAGSM	895.3957	9	896.4106
A5ASG6	AIYPGGAFDPLGLAD	1475.7296	15	738.8816
	AIYPGGAFDPLGLADD	1590.7565	16	796.3926
	ATNFVPGK	832.4443	8	417.2346
	AWAYATNFVPGK	1323.6611	12	662.8443
	AYATNFVPGK	1066.5447	10	534.2838
	PVANNAWAYATNFVPGK	1819.8893	17	910.9619
	YATNFVPGK	995.5076	9	498.7657
	YGWDTAGLSAD	1154.488	11	1155.5073
A5C154	AAAALPNKCGVQTD	1323.6782	14	662.8516
	IPISRSTD	887.4712	8	888.4869
	KCGVQTDIPISR	1363.6765	12	682.8497
	KCGVQTDIPISRSTD	1666.7832	15	834.4077
	LKSLSPTTSD	1047.5448	10	524.7837
	LTGGGAPTS	759.3763	9	760.3911

Accession Number	Peptide sequence	Mass	Length	*m/z*
	LTGGGAPTSA	830.4134	10	831.4279
	LTGGGAPTSACCDGVR	1461.634	16	731.83
	LTGGGAPTSACCDGVRTL	1675.7657	18	838.8986
	VQTDIPISRSTD	1330.6729	12	666.3508
D7TWQ4	IVKEGQLASQYQ	1362.7144	12	682.3575
	PELENQIGAKFEED	1617.7522	14	809.8942
	PELENQIGAKFEEDVD	1831.8475	16	916.9368
	YIVKEGQLASQYQ	1525.7776	13	763.9022
F6GV26	AGGSGAGPKIE	942.477	11	472.25
	DEDGPSAGGAGGSGAGPK	1607.61	18	536.8744
	GPSAGGAGGSGAG	915.4046	13	458.7148
	GPSAGGAGGSGAGPKIE	1448.6296	17	725.3325
	PSAGGAGGSGAGPKIEEVD	1654.7798	19	828.4123
P56648	PQTETKASVGFK	1333.6877	12	667.8572
	YTPEYETKPTDILA	1639.7981	14	820.9155
	YYTPEYETKPT	1390.6293	11	696.3276
	YYTPEYETKPTDILA	1802.8615	15	902.4461
D7T227	AAVPSGASTG	816.3977	10	817.415
	AGYTGKVVIGMD	1225.6012	12	613.8151
	AIIAPALIGKD	1080.6543	11	541.3399
	AVENVNAIIAPALIGKD	1706.9567	17	854.4953
	LPVPAFNVINGGSHAG	1549.7888	16	775.9115
F6GVX0	LAATTAASSLGV	1042.5658	12	522.2969
	LTVIAGTAERAPTL	1411.8035	14	706.9163
	TVIAGTAERAPTL	1298.7194	13	650.373
	VIAGTAERAPTL	1197.6718	12	599.8494
F6HNX5	AGGSGAGPKIE	942.477	11	472.25
	AGKGEGPAIG	897.4556	10	898.4717
	DDGPSAGGSGAGPKIE	1413.6371	16	707.8333
	MYQGAGGPDAGAGAMDDDG	1782.6825	19	892.3662

Accession Number	Peptide sequence	Mass	Length	*m/z*
	PSAGGSGAGPKIEEVD	1469.6997	16	735.8637
F6GSG7	EKEYKPE	903.4338	7	452.7289
	IRNPEEIPWGETGAE	1696.8057	15	849.4154
	SNTLLFGEKPVTVF	1550.8344	14	776.4315
	VVSTDFIGDNRSSIFD	1770.8424	16	886.4362
F6HAU0	DPELDVGIGLR	1182.6244	11	592.3246
	PELDVGIGLR	1067.5974	10	534.8109
	VLLEPGSVVPLDIGSASQL	1893.0459	19	947.5399
F6HKH3	AAVPSGASTG	816.3977	10	817.415
	NVNTIIGPALIGKD	1423.8035	14	712.9155
	TIIGPALIGKD	1096.6492	11	549.3376
F6GXE5	YLSSPGPTRDEID	1448.6783	13	725.3538
	YLSSPGPTRDEIDF	1595.7467	14	798.8867
	YLSSPGPTRDEIDFE	1724.7893	15	863.4067
F6GY46	KPVDDAVPYSDTTYSD	1771.7788	16	886.9054
	VAAAGAYALH	942.4923	10	472.2587
F6H766	IPVVSAAGNSGPSAE	1336.6622	15	669.3466
	PFDFGGGIVNPNRAAD	1645.7848	16	823.9082
F6GTT2	EEHYNTARGVQ	1284.5847	11	643.3046
F6HFL0	PAPATTFAHLDATTVL	1624.8461	16	813.4376
Q9FS43	ALATFKAIEAY	1196.644	11	599.335
	AYVLAHPDAY	1118.5397	10	560.2828
	VLAHPDAY	884.4392	8	443.2316
D7TBH4	PKATVGGGVE	913.4869	10	457.7596
	PKATVGGGVEDLYGED	1605.7522	16	803.8912
D7T674	IEAYTPGSKVTYPIAAD	1794.9039	17	898.4679
	PGLTLGDTIPNLEVETTHG	1962.9897	19	982.517
D7TZ79	PHKAAVIGDTIGD	1292.6724	13	647.3487
D7UA22	PRNPAVIADNVGD	1336.6735	13	669.3491
F6HDI1	AADLIEQLISNK	1313.719	12	657.8734

Accession Number	Peptide sequence	Mass	Length	*m/z*
	DAADLIEQLISNK	1428.746	13	715.3892
F6GUW1	ALIPRFFGNPSVSD	1560.7936	14	781.4114
	PLFGSGEASSL	1063.5186	11	1064.5361
	PLFGSGEASSLANL	1361.6826	14	681.8553
F6HI57	ASGEQIQALSGGQHGE	1567.7227	16	784.8782
	FVVPAGHP	822.4388	8	823.4499
D7SW25	PGDSDIIKTLPGD	1326.6666	13	664.3337
	PGDSDIIKTLPGDH	1463.7256	14	732.8774
D7U9K4	ETHPEVPGEPTHTTT	1631.7427	15	816.8855
	ETHPEVPGEPTHTTTA	1702.7798	16	852.4041
E0CRL1	LPEEGGETSKADEPQAES	1872.8224	18	937.4296
F6H3T7	IVIHNPGVEED	1220.6036	11	611.3151
	VIEIVIHNPGVEED	1561.7987	14	781.914
F6I0K4	PLDVTKTWPE	1184.6077	10	593.3173
D7UD99	PLDVTKTWPED	1299.6346	11	650.8308
F6HS53	AGAGAGGGVGGVGGV	1041.5203	15	521.7798
	GGLGGAGGLGGGAGAGAGGG	1326.6276	20	664.3353
	GGLGGAGGVGGLGGAGGLGGLGGAGGLG	1979.0183	28	660.6805
F6HID6	SVTASGSSITAVEKAN	1521.7522	16	761.8894
F6HPN2	AATGKTFETLP	1152.5663	11	577.2941
	PRTGNVIASVAEGD	1384.6946	14	693.3621
A5B118	AAYIGTPGKGILAAD	1416.7612	15	709.3934
	AKLGEGAAESL	1044.5450	11	523.2845
D7T5P6	DESVIVTIVPR	1226.6870	11	614.3561
F6I2B8	DESVIVTIVPRAGGD	1526.7939	15	764.4104
D7T7C6	SINVSGGHVNPAVTF	1497.7576	15	749.8937
	VALAHGLAL	863.5228	9	432.7730
F6HQY1	YDALDVANKIGII	1403.7660	13	702.9004
D7U8L2	PLFPFGFGLTTEPTK	1650.8657	15	826.4536
	PLFPFGFGLTTEPTKA	1721.9028	16	861.9725

Accession Number	Peptide sequence	Mass	Length	*m/z*
D7TLU7	VDGFLVGGASLKPEF	1534.8031	15	768.4148
F6HJA3	PEKDPLGYNLVD	1358.6718	12	680.3500
D7TXR6	LVAGQTFEAAAEEGHTA	1700.8005	17	851.4158
D7TSJ8	SEVILYPQAIFKD	1521.8079	13	761.9188
F6I0Z8	EKEVSEEGQSHGKDYVD	1934.8494	17	968.4387
D7T4I1	PLVAVLPSFHGIE	1377.7656	13	689.9008
D7TXF5	VTNTATGTQATVR	1318.6841	13	660.3552
F6HMA2	PLVTKGPNLVPLLG	1416.8704	14	709.4496
F6HMA2	PDDIDHAVLIVGYGSED	1813.8369	17	907.9402
F6HNP7	PFEGFPFSTTL	1241.5968	11	1242.6119
Q0ZJ20	IIREPEPEVKILVDRD	1920.0680	16	641.0356
F6HKS7	PLHTTIIDNVFYS	1518.7719	13	760.4023
F6HM78	YDIAGTPGVAADVSHIN	1699.8053	17	850.9174
D7SYI1	IVYGGGSERNPGGF	1408.6735	14	705.3502
F6I2F8	AFLKESALPYL	1250.6910	11	626.3593
D7T0Z5	PTQISFATKQVE	1347.7034	12	674.8656

NP, novo-ProD; AL, alcalase; NZ, novozym; PE, pepsin; FZ, flavourzyme; PA, papain.
